# Variation in stable carbon (δ ^13^C) and nitrogen (δ^15^N) isotope compositions along antlers of Qamanirjuaq caribou (*Rangifer tarandus groenlandicus*)

**DOI:** 10.1002/ece3.11006

**Published:** 2024-03-18

**Authors:** Matthew Brenning, Fred J. Longstaffe, Danielle Fraser

**Affiliations:** ^1^ Department of Earth Sciences Carleton University Ottawa Ontario Canada; ^2^ Palaeobiology Canadian Museum of Nature Ottawa Ontario Canada; ^3^ Department of Earth Sciences The University of Western Ontario London Ontario Canada; ^4^ Department of Biology Carleton University Ottawa Ontario Canada; ^5^ Department of Paleobiology Smithsonian National Museum of Natural History Washington DC USA

**Keywords:** antler, Bayesian stable isotope mixing models, carbon, nitrogen, *Rangifer tarandus groenlandicus*, stable isotope

## Abstract

Annual antler growth begins in the spring and is completed by late summer for male caribou (*Rangifer tarandus groenlandicus*) from the Qamanirjuaq herd (Nunavut, Canada), aligned with both the spring migration and a seasonal dietary shift. Antlers may provide a non‐lethal means of studying short‐ and long‐term changes in caribou ecology through incorporated isotopes of carbon (δ^13^C) and nitrogen (δ^15^N). We sampled the antlers of 12 male caribou from the Qamanirjuaq herd culled in September 1967. We predicted that serial sampling of antlers would reflect the known seasonal dietary change from lichen to grass‐like and shrub diet based on rumen contents from individuals culled during the same period. The δ^13^C and δ^15^N were analyzed in food sources and every 3 cm along each antler's length. The carbon isotope compositions of collagen (δ^13^C_col_) varied by ~0.5‰ among individuals and within antlers, while the carbon isotope compositions of antler bioapatite (δ^13^C_CO3_) increased by 1–1.5‰ from pedicle to tip. Values of δ^15^N_col_ increased within antlers by 1–3‰ from pedicle to tip and varied by 3‰ among the individuals sampled. Antler collagen was lower in δ^15^N_col_ by ~1‰ relative to bone collagen. Bayesian mixing models were conducted to test for changes in dietary proportions from antler isotope compositions. Mixing models did not indicate significant dietary shifts for any individual during antler formation, showing consistently mixed diets of fungi, horsetail, lichen, and woody plants. Increases in δ^15^N_col_ in antler tissue could, therefore, correspond to subtle seasonal dietary changes and/or the physiological stress of antler tissue development.

## Graphical abstract

Stable isotopes in antler tissue may provide a non‐lethal means of studying short and long‐term changes in caribou ecology. Carbon and Nitrogen isotopes of antler collagen showed variation from pedicle to tip and varied among the individuals sampled. Bayesian mixing models, however, did not indicate a significant dietary shift for any individuals during antler formation, showing consistently mixed diets of fungi, horsetail, lichen, and woody plants.

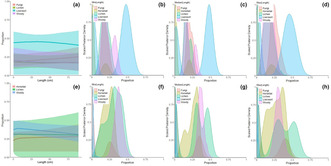

## 1 INTRODUCTION

Caribou (*Rangifer tarandus* Linnaeus, 1758) are a quintessential high‐latitude mammal, being both ecologically and socioeconomically important (Mallory & Boyce, 2018; Wolfe, 2004). They are a keystone species of food webs and an integral food source for carnivores and Indigenous societies (Barber et al., 2018; Latham et al., 2013; Wolfe, 2004). Despite their importance, caribou populations have been on a steady decline, which is linked to both the impacts of anthropogenic modifications of caribou habitat (i.e., forestry, mineral extraction, and petroleum infrastructures) and northern amplification of climate change (i.e., changes in ice sheets, changes in Arctic biota, and seasonal temperature changes) (Anisimov et al., 2007; Mallory & Boyce, 2018; Post et al., 2009). Historically, caribou have adapted to climate change events, including the period of rapid change that occurred at the end of the Pleistocene (20,000–11,700 ybp), which may have contributed to the extinction of the majority of North American megafaunal genera (Parmesan, 2006; Tyler, 2010; Zimov et al., 1995). It is predicted that some caribou herds may similarly adapt to the current environmental changes, through alterations to their migratory behavior and/or changes to their dietary preferences (Parmesan, 2006; Tyler, 2010). In Canada, however, it is projected that many Canadian caribou populations will decrease by as much as 50% in the next 8–15 years (Barber et al., 2018). Herds in decline are unable to adapt to rapid changes from anthropogenic landscape modifications and climate change. The caribou may be unable to adjust the timing of their migrations and experience a phenological mismatch, resulting in an inability to exploit high‐quality foraging periods and, thus, increases in mortality rates (Post & Forchhammer, 2008; Vors & Boyce, 2009). To better understand the effects of climate change on Canadian caribou populations and how they have adapted and might adapt in the future, informative ecological baselines (i.e., the reference state of caribou distributions, abundances, diets, etc.) must first be established (Rodrigues et al., 2019).

Barren‐ground caribou (*Rangifer tarandus groenlandicus*), a subspecies found in northern Canada, Alaska, and Greenland, have been assessed as at risk due to climate change and human landscape modification (Mallory & Boyce, 2018; Parlee et al., 2018). Many Canadian herds of barren‐ground caribou travel long distances (e.g., ~300 km one way) during their migrations, increasing the likelihood that they will traverse regions that are strongly impacted by climate change, whether by increases in wildfires, decreases in ice, or changes to forage availability (Mallory & Boyce, 2018; Post et al., 2009; Post & Forchhammer, 2008; Theoret et al., 2022). Migration is integral to their life history as they move to find better food resources, avoid predation, and reach safe calving grounds (Davidson et al., 2020). In the absence of additional pressures (e.g., no human disturbance, available habitat, and no competition), barren‐ground caribou show substantial flexibility in their migratory patterns to accommodate environmental changes (Mallory & Boyce, 2018). They alter their departure times, avoiding areas affected by wildfires or poor ice conditions/flowing water, and optimizing grazing opportunities (Gunn & Skogland, 1997; Le Corre et al., 2017; Mallory & Boyce, 2018). Historic responses of caribou to long‐term environmental changes (i.e., hundreds to thousands of years), however, are unfortunately inaccessible due to a lack of records from direct observation.

Fortunately, the isotopic compositions of animal tissues can be used to determine dietary and space use changes over long periods of time (Ehleringer & Rundel, 1989; Hobson & Wassenaar, 1999; Koch, 2008). Isotopes are natural tracers that, when consumed, undergo fractionation (i.e., the partitioning of heavy and light isotopes of the same element) during tissue synthesis (e.g., the formation of muscle, bone, and antler tissues) (Ehleringer & Rundel, 1989; Fry, 2006; Hobson & Wassenaar, 1999; Koch, 2008). During tissue synthesis, stable isotopes of carbon (^12^C, ^13^C) and nitrogen (^14^N, ^15^N) are incorporated from consumed food in relative proportions characteristic of its source (Ehleringer & Rundel, 1989; Fry, 2006; Hobson & Wassenaar, 1999; Koch, 2008). Carbon (δ^13^C) and nitrogen (δ^15^N) stable isotope compositions in new tissues therefore reflect the assimilated foods, albeit with an enrichment in the heavy isotopes (^13^C and ^15^N) due to metabolic processes involved in, for example, protein synthesis (Dalerum & Angerbjörn, 2005; Deniro & Epstein, 1978, 1981). Values of δ^13^C and δ^15^N in food sources also vary spatially and temporally due to changes in climate, rainfall, and plant communities (Farquhar et al., 1989; Handley et al., 1999; Kelly, 2000). Thus, if an animal migrates or undergoes seasonal dietary changes during tissue formation, especially during the synthesis of sequentially forming tissues that experience finite growth (e.g., tooth enamel and antler), changes in food source isotopic compositions are potentially detectible in the tissue, providing there is limited remodeling and tissue replacement (Drucker et al., 2010; Gannes et al., 1997; Hedges et al., 2007). Variation in stable isotopes in relation to changes in diet and migration has been demonstrated with tooth enamel, often reflecting the first 6–12 months of an individual's life (Drucker et al., 2012; Fraser et al., 2021; Kohn, 2004; Passey et al., 2002). Antler tissue shows similar finite growth and may therefore record the seasonal dietary changes and migration in a comparable manner (Koch, 2008; Schwartz‐Narbonne et al., 2021).

Antler growth is triggered by a hormonal release, which correlates with the seasonal reproductive cycle (Lincoln, 1992). Both bone and antler tissue are composed of protein collagen and a mineral phase similar to calcium hydroxyapatite [Ca_10_(PO_4_)_6_(OH)_2_] (Chen et al., 2009), commonly known as bioapatite. In contrast to bone tissue, antler does not undergo secondary ossification; instead, tissue growth comprises mineralized cartilage ossifying into a tubular framework, which is then slowly infilled to create a dense antler cortex (Chen et al., 2009; Landete‐Castillejos et al., 2019; Schaefer & Mahoney, 2001; Stevens & O'Connell, 2016). Tissue formation begins on the pedicle bone on the skull. Antler tissue is then formed sequentially, meaning the initial and oldest tissue is laid directly on the pedicle bone with newest growth at the antler tip (Baksi & Newbrey, 1988, 1989; Krauss et al., 2011). Throughout the growth phase, carbon and nitrogen are incorporated into the new tissue with isotopic ratios (^13^C/^12^C) and nitrogen (^15^N/^14^N) characteristic of that period of growth (Stevens & O'Connell, 2016). Thus, any migratory, dietary, and metabolic changes that occur during the tissue growth period may be reflected in stable isotopic variation along the length of antlers. Furthermore, antlers are formed and shed annually among both male and female caribou, providing a non‐lethal means of studying short‐ and long‐term changes in caribou biology using field‐collected or archived specimens. Antlers may therefore represent an untapped source of ecological data for caribou, providing unique and valuable insight that is different from other previously studied tissues like bone.

This paper therefore asks: do variations in stable isotope compositions, specifically δ^15^N and δ^13^C, along the length of caribou antlers change in correlation with known seasonal dietary changes and migration patterns? The herd selected for this study is the Qamanirjuaq herd located in the Keewatin Region of the Canadian Territory Nunavut (Beverly and Qamanirjuaq Caribou Management Board, 2014). Surveys found 496,000 individuals in 1994 but the herd has since declined 2% annually to approximately half that number (Beverly and Qamanirjuaq Caribou Management Board, 2014; Boulanger et al., 2018). First herd measurements occurred in 1955, estimating the herd at 149,000 individuals, a result of excessive hunting allowed during the 1940s (Loughrey, 1955). Today, the Qamanirjuaq herd's range covers a large portion of Nunavut and the Northern portion of Provinces Saskatchewan and Manitoba, including several ecozones (Beverly and Qamanirjuaq Caribou Management Board, 2014; Parker, 1972). The population's decline is not well understood; the relative effects of predation, forage availability, climate, disease, and parasitism remain uncertain (Beverly and Qamanirjuaq Caribou Management Board, 2016; Evans, 2020). The herd is an important socioeconomic and cultural commodity for the Indigenous groups of Inuit, Dene, Métis, and Cree in the region; thus, determining the cause of population decline is critical (Beverly and Qamanirjuaq Caribou Management Board, 2014).

The Qamanirjuaq caribou develop antler tissue during their spring migration, for males, antler growth begins in February and ends before the rut in September. Females grow their antlers from June to October (Parker, 1972). The migration begins with the majority of the herd wintering in the boreal forest of northern Saskatchewan and Manitoba; adult males are scattered throughout as either individuals or in small bands (Parker, 1972). Spring migration begins for the females and young calves in April, as they move north/northeast into the taiga ecozone, while adult males older than 23 months remain in the boreal forest until June (Parker, 1972). Throughout June and July, adult males begin migrating north, while remaining alone or in small bands (Parker, 1972). By late August and mid‐September, all caribou begin to congregate near North Henik Lake in Nunavut in preparation for the rut (i.e., mating season) (Parker, 1972). After the rut in October, the fall migration begins as all caribou head south toward the tree line (Parker, 1972).

Seasonal dietary variation is known to occur among Qamanirjuaq caribou. Arctic seasonal changes drive variations in the availability of certain food sources (Miller, 1976). In addition, the Qamanirjuaq migration route brings individuals between wintering grounds in boreal forest and summer ranges in the Arctic taiga (Parker, 1972). During the winter, lichen is a predominant food source for the caribou, with a small portion of the diet coming from other plants such as birch, willow, and mosses (Miller, 1976). With seasonal change the Qamanirjuaq consumption of lichen slowly decreases, by April the diet is almost 50% grasses and rush‐like plants (i.e., sedges and horsetail) (Miller, 1976). By June, the diet becomes almost entirely grass‐like plants and shrubs (i.e., woody plants) (Miller, 1976). Herein, we evaluate patterns of stable isotope variation to understand how known dietary changes among male caribou during the spring migration may be reflected in their antler tissues.

## 2 METHODS

### 2.1 Archived caribou samples

A Canadian Wildlife Services (CWS) study conducted in the late 1960s culled 999 caribou. Of these animals, 943 were from the Qamanirjuaq population and 56 from the Beverly population. Culling was conducted to better understand the biology of the caribou population with specific interest in determining age, sex, foraging habits, and growth, as well as physical and pathological conditions (Dauphine, 1976; Miller, 1974, 1976; Parker, 1972). During the study rumen contents, skulls, antlers, teeth, and bone were collected (Dauphine, 1976; Miller, 1974, 1976; Parker, 1972). The project collected caribou over 2 years between April 1966 to July 1968 to determine seasonal and annual changes to the biology of the caribou. Culled individuals were sexed, weighed, and total length of their body measured, and their rumen contents were collected (Miller, 1974). Skulls, including teeth and bone, were carefully scrubbed clean with a stiff‐bristle brush after being soaked in hot water for several days (Dauphine, 1976). The age of each specimen was determined through dental measurements of the right mandible, with the left being archived (Parker, 1972). Skulls and antlers are currently housed at the Canadian Museum of Nature (CMN).

From the culled caribou of the CWS study, 12 adult males were selected. All caribou were ~50 months in age, of similar weight and body size (i.e., 122 kg and 180 cm), and were all culled during September 1967, specifically between September 15th and the 21st (Table S1). Selected males were all collected at the same location with the assumption that they were part of the same migrating band, thereby potentially minimizing dietary differences. The CWS report indicated that the antlers with velvet membranes were not cleaned. No antler membrane tissue remained on the samples selected for the present study, indicating completed antler growth. Antler and mandible bone samples were selected from the 12 males for stable isotope analysis.

### 2.2 Archived plant samples

Plant and lichen samples were collected from 78 herbarium specimens (32 different species and 8 different genera) housed in the National Herbarium of Canada at the CMN. Plant and lichen species were selected based on proximity to the Qamanirjuaq habitat range and relative age from 1965 to 1968 (Table S2). Additionally, all sampled plant species had been observed by the CWS study in rumen samples collected from the culled caribou between 1966 and 1968 (Miller, 1974). Fungi data were obtained from the literature. We used isotopic compositions for fungi from Hobbie et al. (2017) because they collected commonly consumed Arctic fungal food sources from multiple sites with differing soil acidities to account for isotopic variability (Table S3) (Hobbie et al., 2017).

### 2.3 Sample preparation for stable isotope analysis

A total of 12 antlers and bones were collected and prepared for collagen carbon (δ^13^C_col_) and nitrogen (δ^15^N_col_) isotope analysis. Both skeletal bone and antler were sampled for comparative purposes; antler has been found to be isotopically distinct from bone in red deer and the diet composition from bone has been thoroughly studied (DeNiro, 1985; Stevens & O'Connell, 2016). Results for bone and antler tissue were thus compared using a Wilcoxon signed‐rank test. The surface of the antler tissue or mandible bone was cleaned through the removal of at least 3 μm of material. The cleaned surface was then drilled using an 8220 Micro 12Vmax High Performance Cordless Dremel© tool with a 3/32‐inch Diamond Wheel Taper Point Rotary Bit to collect antler and bone powder for analysis. For each caribou, antlers were sampled every 3 cm along the beam of the antler, and bones were sampled once on the right mandible. After each use of the Dremel tool, it was cleaned with deionized water.

For the 12 antlers and bones, a total of 334 antler samples and 12 bone samples were collected. For collagen preparation, 2 mg of sample was weighed out and placed into separate plastic microcentrifuge tubes with 1.5 mL of 0.1 M HCl solution (initial procedure suggested 1 M HCl but was reduced to avoid the loss of nitrogen). Tubes were covered with aluminum foil caps and placed into a refrigerator for 30 min to allow the samples to decalcify. Published research procedures suggested a 2‐day decalcification period but this process caused the complete degradation of antler powder material and loss of nitrogen (Trayler et al., 2023). The resulting solution was aspirated and then rinsed using 1 mL of deionized water, which was subsequently agitated, centrifuged, and aspirated again. This whole process was repeated five times before samples were placed in the freeze dryer overnight to remove the remaining water. Since antlers do not contain enough fat to require defatting (Chen et al., 2009), this step was omitted from the usual collagen preparation procedure (most studies typically use a petroleum ether wash to remove fats from bone). Defatting processes have also been shown to affect the nitrogen isotope composition of the remaining collagen (Chen et al., 2009; Elliott & Elliott, 2016; Logan & Lutcavage, 2008).

A subset of the 12 male Qamanirjuaq caribou were then selected for bioapatite carbon (δ^13^C_CO3_) isotope analysis. Differences between δ^13^C_CO3_ and δ^13^C_col_ might allow us to discern dietary changes affecting the whole diet from those affecting the carbohydrate component of the diet. Four of the largest antlers (i.e., most samples per antler) were analyzed for a total of 138 of the 334 antler samples. Antler tissue samples were prepared for δ^13^C_CO3_ isotope analysis following standard methods for isolating structural carbonate (Longin, 1971; O'Connell et al., 2001). One (1) mL of 2%–3% NaOCl (bleach) was added to 3 mg of bone powder to isolate carbonate tissue. Samples were agitated and then left to sit in a refrigerator for 24 h. The solution was then centrifuged, aspirated, and new NaOCl solution was added. After another 24 h of refrigeration, the samples were centrifuged and the NaOCl solution aspirated. Five washes were conducted for each sample, each time adding ~1 mL of deionized water, agitating the sample, centrifuging, and aspirating the water. The standard sampling procedure suggests adding 0.5 mL of 1 M acetic acid buffered with calcium acetate to a pH of 5, but this approach was omitted due to the tendency of the fine antler tissue to dissolve completely (Chen et al., 2009; Koch et al., 1997; Longin, 1971). The omitted acetic acid protocol is a necessary step to eliminate exogenous carbonate contamination resulting from processes occurring after the antler was discarded into the environment (Koch et al., 1997). However, since the current samples are modern, and collected during a cull, exogenous carbonate contamination was not expected.

Samples of likely caribou forage were obtained from the National Herbarium of Canada. Those samples had been previously dehydrated for long‐term storage. Those plant and lichen specimens were first cut and then ground into a fine powder in preparation for isotopic analysis.

### 2.4 Stable isotope analysis

The stable carbon and nitrogen isotope and elemental compositions of the antler and bone collagen and bioapatite structural carbonate were analyzed at the Laboratory for Stable Isotope Science (LSIS), The University of Western Ontario. All isotopic results are reported using the typical delta (δ) notation in parts per thousand (‰) relative to VPDB and AIR, respectively (Craig, 1957; Junk & Svec, 1958). All analytical errors in the present study are reported to one standard deviation (1σ) (Tables S4 and S5).

For analyses of collagen, ~0.3–0.5 mg of each sample and suitable standards were weighed into tin capsules. The capsules were then gently crimped and loaded into an autosampler atop a Costech™ ECS 4010® elemental analyzer (EA) interfaced with a Thermo Scientific™ Delta^plus^ XL® isotope ratio mass spectrometer (IRMS). Gases (CO_2_, N_2_) released by combustion of the samples or standards were swept to the IRMS in continuous‐flow (CF) mode using helium as the carrier gas. Standards were analyzed at the beginning and end of each analytical session (10 in total) and after every five samples; all sessions were free of instrumental drift. The stable carbon and nitrogen isotope compositions, the amount (wt.%) of each element, and the C/N ratio for each sample and standard were collected within the same analytical session.

Calibration of the measured isotopic ratios to VPDB and AIR, respectively, was performed using USGS40 (l‐glutamic acid; *n* = 39; 1σ δ^13^C = 0.05‰, accepted δ^13^C = −26.39‰; 1σ δ^15^N = 0.14‰, accepted δ^15^N = −4.52‰) and USGS41a (l‐glutamic acid; *n* = 40; 1σ δ^13^C = 0.09‰, accepted δ^13^C = +36.55‰; 1σ δ^15^N = 0.24‰, accepted δ^15^N = +47.55‰). The accuracy of the calibration curve was tested using the LSIS internal standard (keratin, MP Biomedicals Inc., Cat No. 90211, Lot No. 9966H) (measured δ^13^C = −24.05 ± 0.09‰, *n* = 53; measured δ^15^N = +6.53 ± 0.15‰; *n* = 51), which compared well with its accepted values (δ^13^C = −24.05‰; δ^15^N = +6.40‰; *n* = 1999). The calibration‐curve accuracy for carbon was also tested using IAEA CH6 (measured δ^13^C = −10.41 ± 0.05‰, *n* = 14), which compared well with its accepted value (δ^13^C = −10.45‰). Duplicate analyses of samples differed by an average of 0.08 ± 0.08‰ for δ^13^C (*n* = 38) and 0.16 ± 0.14‰ for δ^15^N (*n* = 35). Collagen carbon and nitrogen contents averaged 38.8 ± 6.9 wt.% and 14.1 ± 2.9 wt.%, respectively (*n* = 350). The average C/N ratio of samples was 3.25 ± 0.18; the average difference between C/N ratios of duplicate samples was 0.07 ± 0.08 (*n* = 35). The carbon and nitrogen abundances and the C/N ratios all lie well within the range characteristic of fresh/well‐preserved collagen (i.e., 2.9–3.6) (DeNiro, 1985).

The stable carbon isotope composition of bioapatite structural carbonate (δ^13^C_CO3_) was measured using a Micromass™ Multiprep® carbonate preparation system (including a Gilson autosampler) directly attached to a Micromass™ VG Optima® IRMS. To make these measurements, ~0.4–0.6 mg of each treated sample and 0.04–0.06 mg of appropriate carbonate standards were weighed into ultraclean glass vials, together with a small quantity of silver wool (the latter to sequester S‐bearing evolved gases). A septum‐lined cap was then screwed onto the vial, and the vials were then loaded into an isothermal, temperature‐controlled rack. For each analytical session (15 in total), standards were positioned in the rack at the beginning and end of the available slots and after about every sample. Each vial was then evacuated via the Gilson autosampler needle and pumping system, followed by the sequential addition of an excess of H_3_PO_4_ to each sample and standard. The ensuing reaction then proceeded under vacuum at 90°C for 25 min. The evolved gases (H_2_O, CO_2_) were then released, passed through a water trap and the carbon dioxide cryofocused into a cold finger. This CO_2_ was then released and transferred to IRMS for analysis in dual‐inlet mode following Metcalfe et al. (2009).

All analytical sessions were free of instrumental drift. Values of δ^13^C_sc_ were normalized to VPDB using NBS 19 (*n* = 45; 1σ δ^13^C = 0.05‰, accepted δ^13^C = +1.95‰) and LSVEC (*n* = 30; 1σ δ^13^C = 0.21‰, accepted δ^13^C = −46.6‰). NBS 18 (accepted δ^13^C = −5.01‰), WS‐1 (accepted δ^13^C = +0.76‰), and ‘Suprapur' (Merck Suprapur® CaCO_3_; accepted δ^13^C = −35.55‰) were used to assess analytical precision and accuracy. Measured values all compared well with accepted values: NBS 18, δ^13^C = −5.05 ± 0.07‰ (*n* = 39); WS‐1, δ^13^C = +0.80 ± 0.07‰ (*n* = 63), and Suprapur, δ^13^C = −35.69 ± 0.18‰ (*n* = 21). Duplicate analyses of sample δ^13^C differed by an average of 0.18 ± 0.26‰ (*n* = 63).

The stable carbon and nitrogen isotope and elemental compositions of half of the plant and lichen samples were analyzed at LSIS using the same instrumentation and approach as described earlier for collagen. In this case, however, the isotopic composition of carbon and nitrogen was analyzed in separate analytical sessions (four sessions in total), given the samples' generally high C/N ratios. For carbon, 0.35–0.6 mg of sample and standards were used for analysis. For nitrogen, weights of standards used were as for carbon, but sample amounts ranged from 1 to 22 mg, based on a preliminary assessment of nitrogen abundance derived from the carbon isotope analytical sessions.

At LSIS, calibration of the measured isotopic ratios to VPDB (carbon) and AIR (nitrogen) for the plant samples was performed as for collagen: USGS40 (l‐glutamic acid; *n* = 7, 1σ δ^13^C = 0.01‰; *n* = 7, 1σ δ^15^N = 0.04‰) and USGS41a (l‐glutamic acid; *n* = 8, 1σ δ^13^C = 0.03‰; *n* = 5, 1σ δ^15^N = 0.23‰). Accuracy was evaluated using the LSIS internal standard keratin described above (measured δ^13^C = −24.06 ± 0.03‰, *n* = 14; measured δ^15^N = +6.43 ± 0.10‰; *n* = 10), which compared well with its accepted values (see above). The calibration‐curve accuracy for carbon was also tested using IAEA CH6 (measured δ^13^C = −10.51 ± 0.03‰, *n* = 6), which compared well with its accepted value (see above). Sample duplicate analyses differed by an average of 0.04 ± 0.04‰ for δ^13^C (*n* = 7) and 0.10 ± 0.11‰ for δ^15^N (*n* = 5). Elemental contents varied widely across the functional groups, ranging from 28.9 to 48.4 wt.% for carbon and 0.3 to 4.7 wt.% for nitrogen. Duplicate analyses differed by 0.7 ± 0.8 wt.% for average carbon contents of 40.2 wt.% (*n* = 8), and by 0.02 ± 0.03 wt.% for average nitrogen contents of 1.9 wt.% (*n* = 4). The C/N ratio (atomic %) of samples ranged from 10 to 166, depending on the functional group.

The stable carbon and nitrogen isotope and elemental compositions of the remaining plant and lichen samples were analyzed at the Ján Veizer Stable Isotope Laboratory at the University of Ottawa due to time constraints. Depending on sample type, 4–30 mg of powdered sample were weighed into tin capsules, loosely crimped, and then loaded into an autosampler affixed to an Elementar Vario EL Cube interfaced with Thermo Scientific™ Delta Advantage® IRMS. Gases (CO_2_, N_2_) released by combustion were swept by helium in continuous‐flow (CF) mode to the IRMS.

At the Veizer laboratory, normalization of the stable carbon isotope results was performed using three in‐house standards (nicotinamide, ammonium sulfate + sucrose, caffeine), each calibrated to VPDB using international standards IAEA‐CH‐6 (accepted δ^13^C = −10.4‰), NBS‐22 (accepted δ^13^C = −29.91‰), USGS40 (accepted δ^13^C = −26.24‰) and USGS41 (accepted δ^13^C = +37.76‰). Analytical accuracy and precision were evaluated using internal standard C‐55 (glutamic acid), which returned δ^13^C = −28.4 ± 0.18‰ (*n* = 6) and compares well with its accepted value (−28.53‰). Normalization of the nitrogen isotope results was performed using the same three in‐house standards, each calibrated to AIR using international standards IAEA‐N1 (accepted δ^15^N = +0.4‰), IAEA‐N2 (accepted δ^15^N = +20.3‰), USGS40 (accepted δ^15^N = −4.52‰) and USGS41 (accepted δ^15^N = +47.57‰). Analytical accuracy and precision were evaluated using the internal standard C‐55 (glutamic acid), which returned δ^15^N = −3.8 ± 0.17‰ (*n* = 6) and compares well with its accepted value (−3.98‰). Duplicate analyses of samples differed by an average of 0.15 ± 0.21‰ for δ^13^C (*n* = 2) and 1.30 ± 1.4‰ for δ^15^N (*n* = 2). Element abundances varied widely across the functional groups, ranging from 36.2 to 51.9 wt.% for carbon and 0.2 to 3.5 wt.% for nitrogen. Duplicate analyses differed by 0.5 ± 0.1 wt.% on average carbon contents of 42.3 wt.% (*n* = 2), and by 0.05 ± 0.07 wt.% on average nitrogen contents of 0.53 wt.% (*n* = 2). The C/N ratio (atomic %) of samples ranged from 16 to 266, depending on the functional group.

### 2.5 Estimating diet from antler stable isotope compositions

To estimate diet from antler tissue, we used Bayesian stable isotope mixing models (BSIMMs) (Franco‐Trecu et al., 2013; Parnell et al., 2010; Phillips & Gregg, 2003). BSIMMs treat the animal tissue as an isotopic mix from consumed prey or plant species. Model outputs include the relative proportion for each of the food source (Franco‐Trecu et al., 2013; Stock et al., 2018). BSIMMs also allow the incorporation of informative priors, an assumed probability distribution based on prior evidence (Moore & Semmens, 2008). Informative priors add weight to a model's sources, which can help separate between sources that are isotopically similar (Stock et al., 2018). Additionally, elemental concentrations of food sources can be added to BSIMMs to more accurately estimate the source contribution to the overall isotopic mix (Hopkins & Ferguson, 2012; Phillips & Koch, 2002). There are many assumptions made in a BSIMMs; they assume that data for all potential food sources have been included in the model, that all collected isotope compositions for source and consumer are normally distributed around the mean, and that isotopic fractionation between diet and consumers is consistent among species (Bond & Diamond, 2011; Stock & Semmens, 2016). Recent developments in BSIMM models like MixSIAR (from open‐source program R) allow the inclusion of more comprehensive error structures to allow greater precision in diet estimation (R Core Team, 2020; Stock et al., 2018).

Each set of stable isotope data for individual antlers was analyzed using multiple BSIMMs in MixSIAR to estimate dietary inputs. To minimize the number of food sources in the model, we experimented with alternative methods of dividing the food sources into groups, including by ecosystem (i.e., Arctic or Taiga). We found the best isotopic separation of food sources was to separate broadly along taxonomic lines. Analysis of variance was then used to determine the statistical separation among the isotopic signatures of functional categories: fungi, horsetails, lichens, liverworts, and woody plants. For caribou, we used a trophic enrichment factor of 3.76 ± 0.26‰ for δ^13^C_col_ and 4.49 ± 0.33‰ for δ^15^N_col_ based on an experimental feeding study that measured mean C and N isotope trophic shifts in white‐tailed deer antler (*Odocoileus virginianus*) (Darr & Hewitt, 2008). We opted to use an empirically derived trophic enrichment factor from a closely related species over estimating a trophic enrichment factor using SIDER (Healy et al., 2018).

For the mixing model, antler length was set as a continuous covariate affecting antler isotope compositions. Models produced four plots; the first shows how mixture proportions change over the length of the antler and the additional three plots show mixture proportions at the minimum length (i.e., the pedicle), the median length (i.e., the middle), and the maximum length (i.e., the tip). A second set of mixing models was also produced using informative priors based on known prior data from foods found in the rumens of a subset of the culled caribou. Miller (1976) sampled and categorized forage items found in the rumen of culled individuals during the CWS study. The average percentage of each food source was used to create the following informative priors; fungi 0.1, horsetail 0.16, lichen 0.24, liverwort 0.13, and woody plants 0.36 (Miller, 1976).

BSIMM models were also conducted without using antler length as a continuous covariate and thus all possible model factors that could impact antler isotopic compositions were set to null. The antlers of Qamanirjuaq male caribou grow during spring, summer, and fall. Different food sources were available during these times, which was reflected in dietary changes observed for the Qamanirjuaq by Miller (1976) and Parker (1972). To determine if antlers demonstrate forage availability, they were divided into three segments to represent the different seasons. BSIMMs were conducted on a lower, middle, and upper segment of each antler for spring, summer, and fall diets respectively. Dietary proportions were compared among the three segments and to the whole antler. Leave‐one‐out information criterion (LOOIC) was conducted to compare BSIMM models; LOOIC was used to compare models using both whole antler tissue and segmented antler tissue. All reported mixing models were set with the following parameters: number of chains = 3, chain length = 100,000, burn = 50,000, thin = 50 (See Supplementary Code ‘ESMcode.R’ in Appendix S2).

## 3 RESULTS

### 3.1 Food source isotopes

**FIGURE 1:**
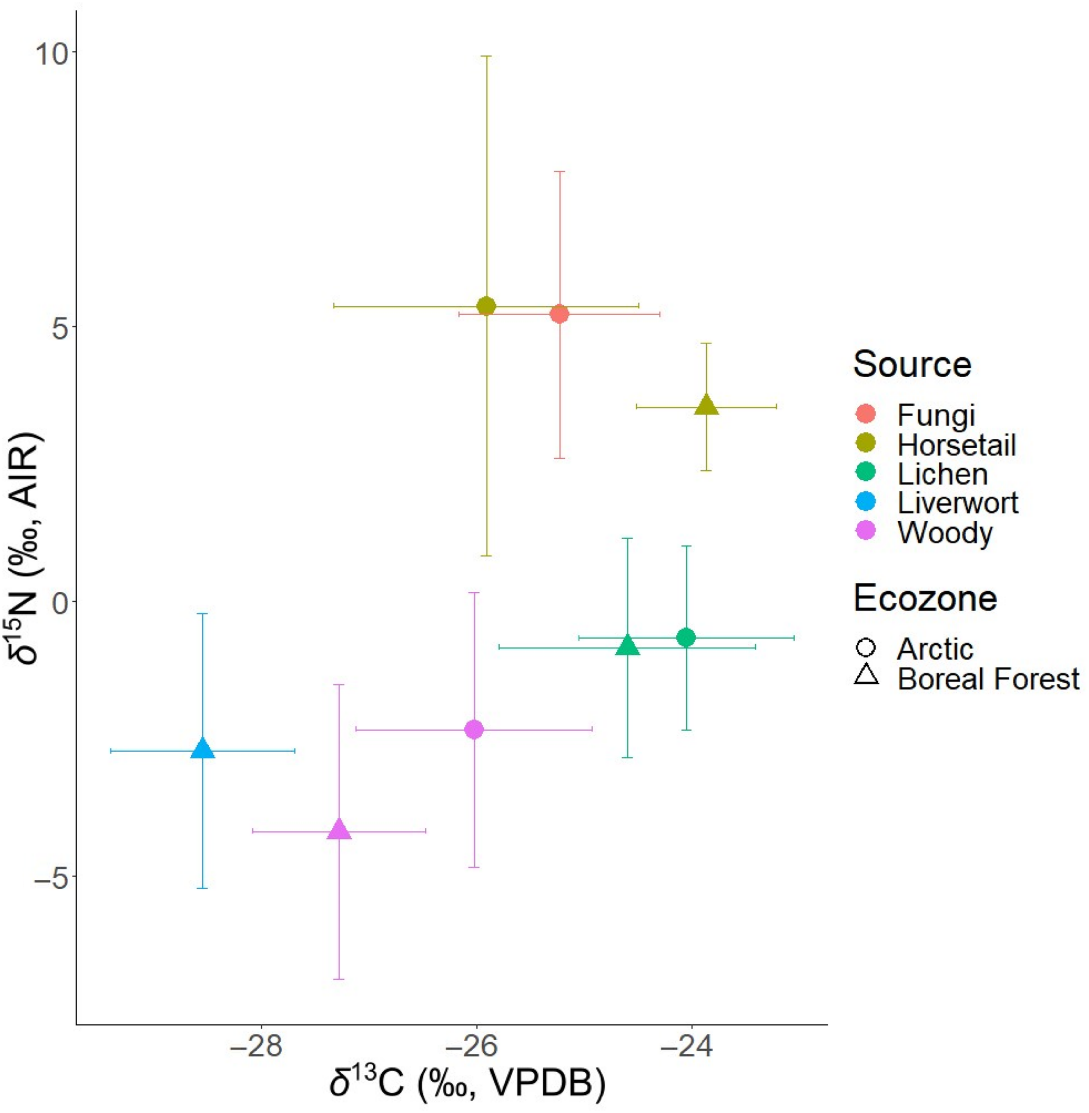
δ^13^C and δ^15^N of sampled food sources by functional group and ecozone.

We found a high degree of overlap in the isotope compositions of δ^13^C and δ^15^N among potential caribou food sources, regardless of how they were categorized. This is partially due to our small sample sizes, which showed a high degree of variation (Table 1; Figure 1; food sources with antler tissue see Figure S1).

**TABLE 1:** Statistical comparison of food source functional groups for δ^13^C and δ^15^N.

Isotope	Functional group	No of samples	Mean (‰)	SE	Univariate significance test	Comparison	Tukey's test
δ^13^C	Fungi	12	−25.23	±0.51	Single‐factor ANOVA, df = 4, *p* < .0001	Horsetail–Fungi	*p* = .99
	Horsetail	9	−25.00	±0.95		Lichen–Fungi	*p* = .37
	Lichen	45	−24.55	±0.35		Liverwort–Fungi	*p* < .0001*
	Liverwort	4	−28.53	±0.73		Woody–Fungi	*p* = .008
	Woody	21	−26.69	±0.48		Lichen–Horsetail	*p* = .82
						Liverwort–Horsetail	*p* < .0001*
						Woody–Horsetail	*p* = .004
						Liverwort–Lichen	*p* < .0001*
						Woody–Lichen	*p* < .0001*
						Woody–Liverwort	*p* = .03
δ^15^N	Fungi	12	+5.22	±1.44	Single‐factor ANOVA, df = 4, *p* < .0001	Horsetail–Fungi	*p* = .97
	Horsetail	9	+4.56	±2.16		Lichen–Fungi	*p* < .0001*
	Lichen	45	−0.82	±0.57		Liverwort–Fungi	*p* < .0001*
	Liverwort	4	−2.73	±2.15		Woody–Fungi	*p* < .0001*
	Woody	21	−3.30	±1.15		Lichen–Horsetail	*p* < .0001*
						Liverwort–Horsetail	*p* < .0001*
						Woody–Horsetail	*p* < .0001*
						Liverwort–Lichen	*p* = .55
						Woody–Lichen	*p* = .002
						Woody–Liverwort	*p* = .99

Abbreviation: ANOVA, analysis of variance.

* Statistically significant results.

Values of δ^15^N showed the greatest differences among potential food sources, with fungi and horsetail results being significantly higher than lichen, liverwort, and woody plants (e.g., single‐factor ANOVA, df = 4, *p* < .01; Figure 1; Table 2). Values of δ^13^C_col_ did not vary to the same degree as δ^15^N_col_; lichen, fungi, and horsetail showed similar overall compositions, with values for woody plants and liverwort being much lower (e.g., single‐factor ANOVA, df = 4, *p* < .01; Figure 1; Table 1). Variation between functional groups was not statistically significant across the two ecosystems of the Arctic and boreal forest for either isotope (e.g., two‐factor ANOVA, df = 4, *p* > .01; Figure 1; Table 2).

**TABLE 2:** Statistical comparison of functional groups between ecosystems for δ^13^C and δ^15^N.

Isotope	Ecosystem	Functional group	No of samples	Mean (‰)	SE	Bivariate significance test	Comparison	Tukey's test
δ^13^C	Arctic	Fungi	12	−25.23	±0.51	Two‐factor ANOVA, df = 4, *p* < .0001	Fungi:Boreal–Fungi:Arctic	*p* = NA
		Horsetail	5	−25.91	±1.13		Horsetail:Boreal–Horsetail:Arctic	*p* = .15
		Lichen	5	−24.05	±0.80		Lichen:Boreal–Lichen:Arctic	*p* = .99
		Liverwort	0	NA	NA		Liverwort:Boreal–Liverwort:Arctic	*p* = NA
		Woody	10	−26.03	±0.66		Woody:Boreal–Woody:Arctic	*p* = .21
	Boreal	Fungi	0	NA	NA			
		Horsetail	4	−23.87	±0.56			
		Lichen	4	−24.61	±0.37			
		Liverwort	40	−28.55	±0.74			
		Woody	11	−27.29	±0.46			
δ^15^N	Arctic	Fungi	12	+5.22	±1.44	Two‐factor ANOVA, df = 4, *p* < .0001	Fungi:Boreal–Fungi:Arctic	NA
		Horsetail	5	+5.37	±3.64		Horsetail:Boreal–Horsetail:Arctic	*p* = .98
		Lichen	5	−0.66	±1.34		Lichen:Boreal–Lichen:Arctic	*p* = 1.0
		Liverwort	0	NA	NA		Liverwort:Boreal–Liverwort:Arctic	NA
		Woody	10	−2.33	±1.50		Woody:Boreal–Woody:Arctic	*p* = .75
	Boreal	Fungi	0	NA	NA			
		Horsetail	4	+3.54	±1.01			
		Lichen	4	−0.84	±0.62			
		Liverwort	40	−2.73	±2.16			
		Woody	11	−4.18	±1.54			

Abbreviation: ANOVA, analysis of variance.

### 3.2 Caribou isotopes

**FIGURE 2:**
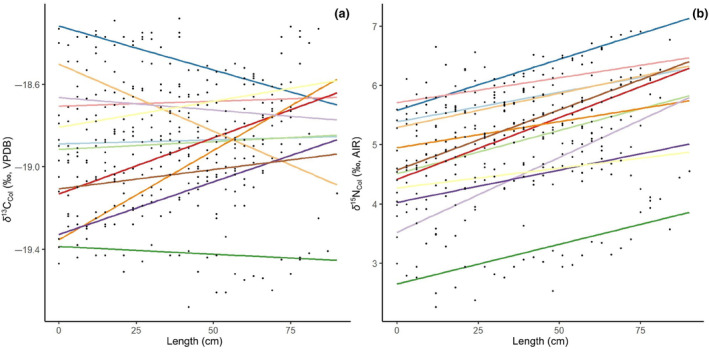
(a) δ^13^C_col_ variation along antler length with linear regressions for each individual; (b) δ^15^N_col_ variation along antler length with linear regressions for each individual.

**FIGURE 3:**
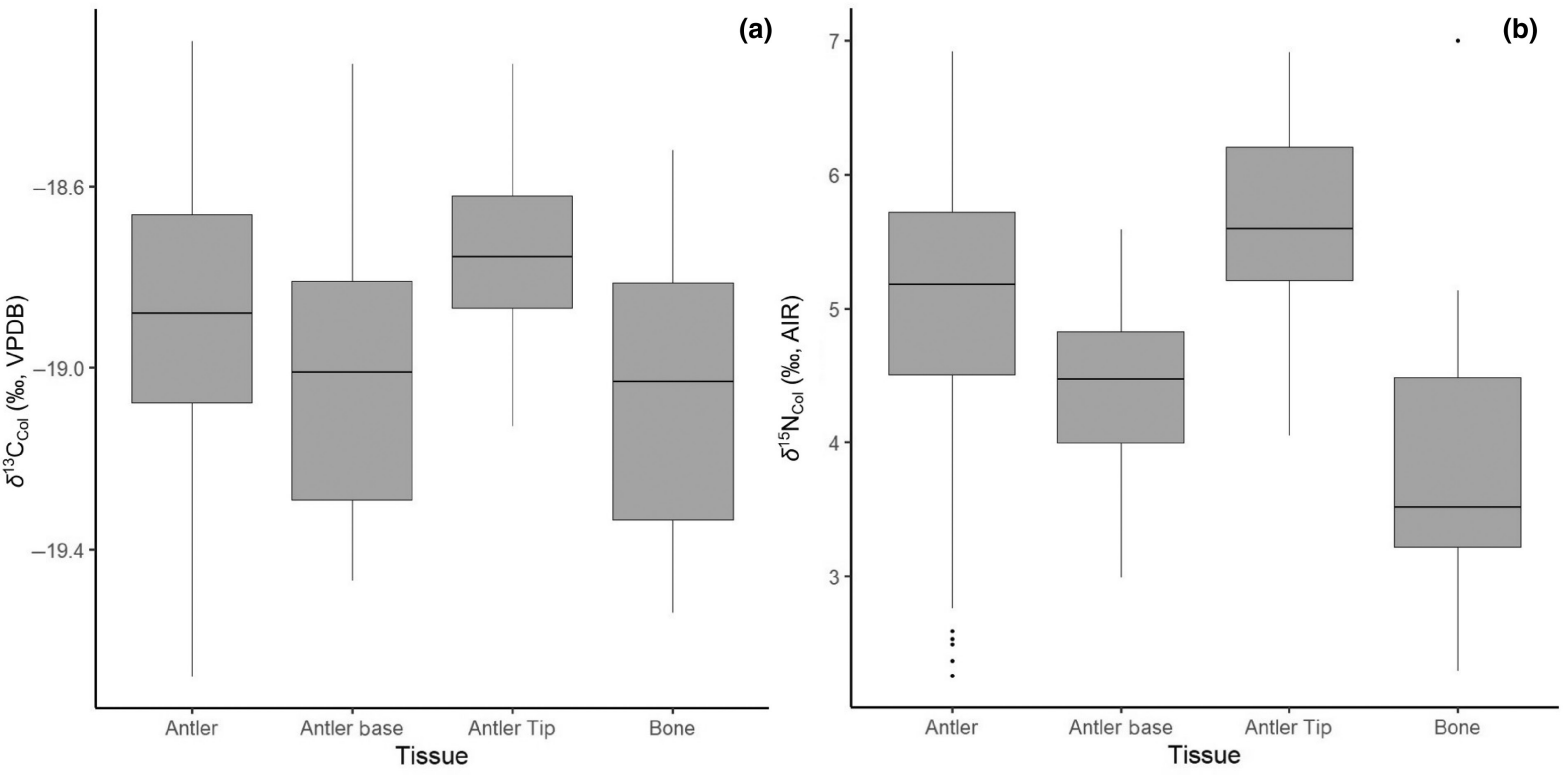
(a) δ^13^C_col_ for all antler samples, antler tips, and antler bases compared to bone samples; (b) δ^15^N_col_ for all antler samples, antler tips, and antler bases compared to bone samples.

Antler collagen δ^13^C_col_ and δ^15^N_col_ for all sampled individuals ranged from −19.7 to −18.3‰ and +2.3 to +6.9‰, respectively. Antler isotope compositions showed a high degree of within and among individual variability (Figure 2). Generally, δ^15^N_col_ increased along the length of antlers (by 1–3‰, with significant linear regressions; Figure 2b; Table S6), while δ^13^C_col_ fluctuated within antlers, some individuals showing increases, some decreases, and still others no trend (only 39090, 39110, 39120, 39132, 39148, and 39151 showed significant linear regressions) (Figure 2a; Table S6). In comparison, average bone δ^13^C_col_ was similar to whole antler tissue, closely resembling antler base tissue (i.e., −19.08 ± 0.34‰ and −18.89 ± 0.31‰, respectively; Wilcoxon signed‐rank test, *p* = .072; Figure 3). Bone had significantly lower δ^15^N_col_ than antler (+3.95 ± 1.29‰ and +5.04 ± 0.97‰; Wilcoxon signed‐rank test, *p* = .00119; Figure 3).

Generally, we found that the sampled antlers could be divided into two groups, those that had a small increase in δ^15^N_col_ and no apparent trend in δ^13^C_col_ from antler base to tip, and those that displayed considerable change during growth in both δ^15^N_col_ and δ^13^C_col_. Antlers CMN39079, CMN39090, CMN39108, CMN39120, CMN39145, and CMN39148, for example, are all from the former group and exhibited variation in δ^15^N_col_ of ~1‰ (i.e., less than one trophic level) and no apparent trend in δ^13^C_col_ (Figure S2).

In contrast, CMN39102, CMN39107, CMN39110, CMN39132, CMN39149, and CMN39151 exhibited considerable change in δ^13^C_col_ and δ^15^N_col_. For example, CMN39110 and CMN39151 both showed increases in δ^15^N_col_ of 2–3‰ (i.e., an entire trophic level) from pedicle to antler tip. Both also showed large, apparent cyclical changes in δ^13^C_col_ of ±1‰ (Figure S2).

**FIGURE 4:**
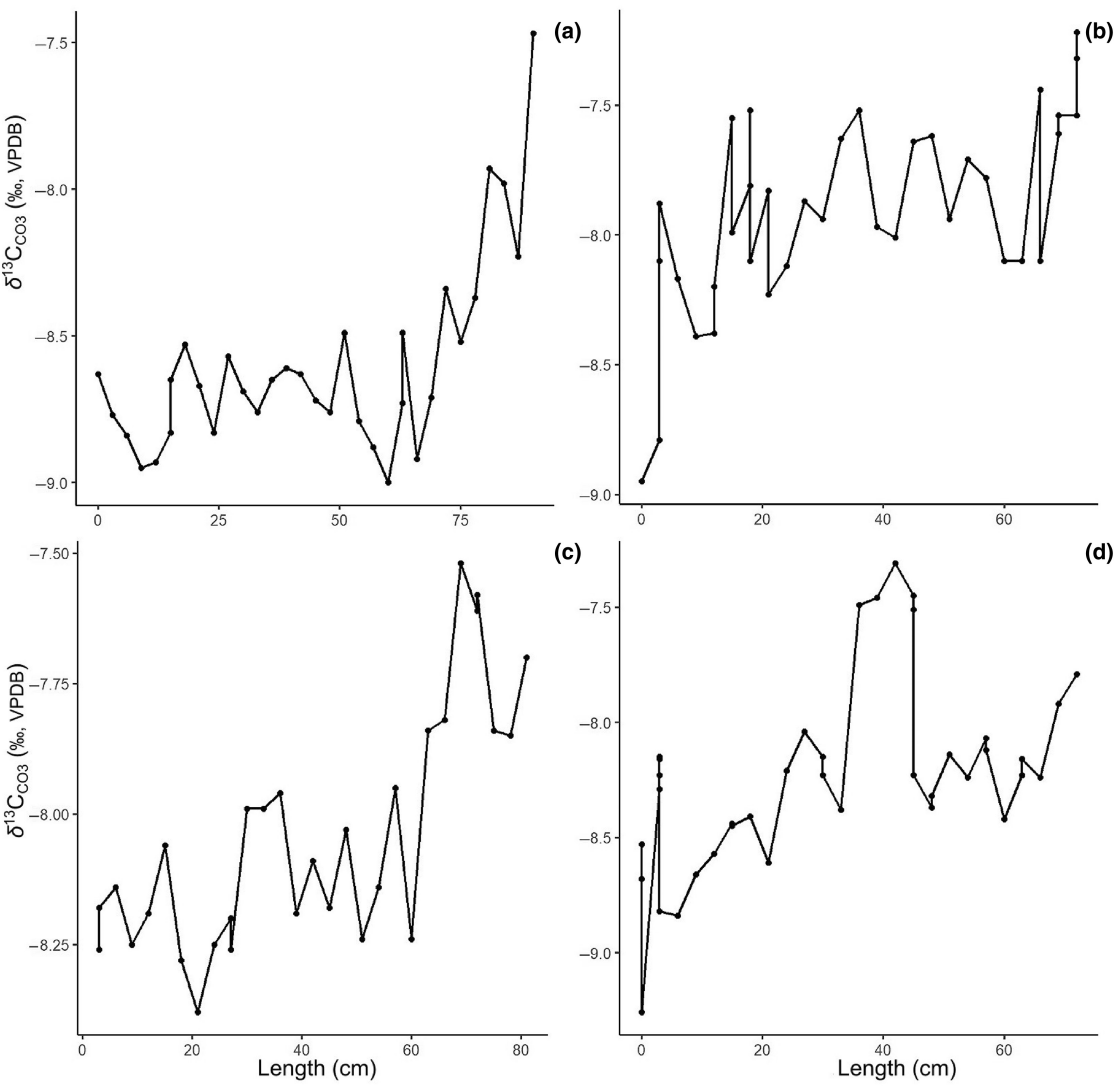
δ^13^C_CO3_ variation along antler length: (a) CMN39107; (b) CMN39108; (c) CMN39145; (d) CMN39148.

The δ^13^C_CO3_ of the analyzed subset of caribou ranged from −9.3 to −7.2‰ with a 1 to ~1.5‰ increase along the length of the antler (Figure 4). Trends within the four antlers sampled were consistent with the pedicle of the antler averaging −8.6 ± 0.2‰ and increasing to −7.6 ± 0.2‰.

### 3.3 Bayesian stable isotope mixing models

**FIGURE 5:**
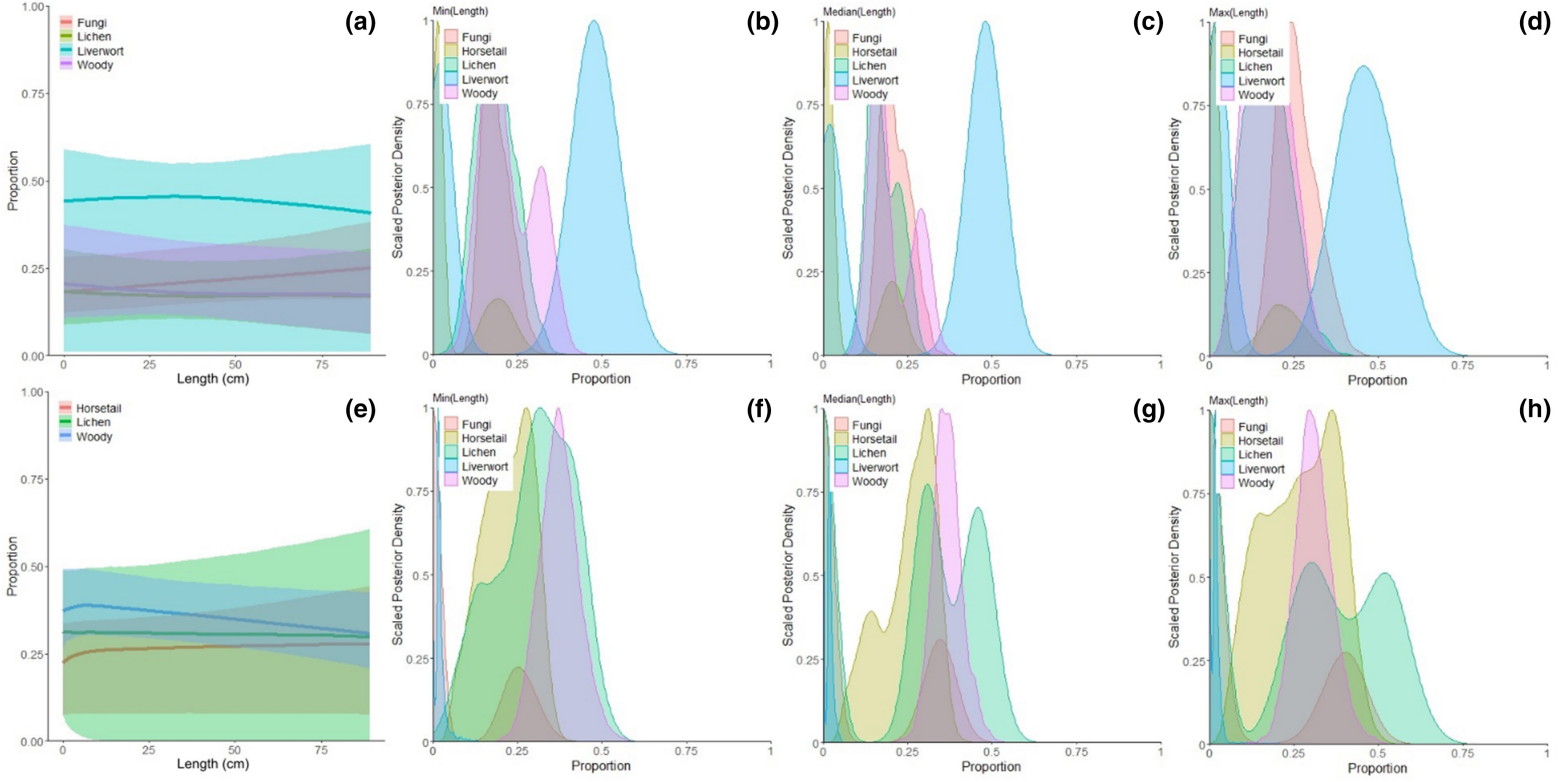
Estimated average diet mixture proportions along all antlers sampled. (a) uninformative priors; (b–d) estimated diet mixture from uninformative priors at specific sub‐samples throughout the antler (left to right): pedicle, middle, and tip, respectively. (e) informative priors; (f–h) estimated diet mixture from informative priors at specific sub‐samples throughout the antler (left to right): pedicle, middle, and tip, respectively.

**FIGURE 6:**
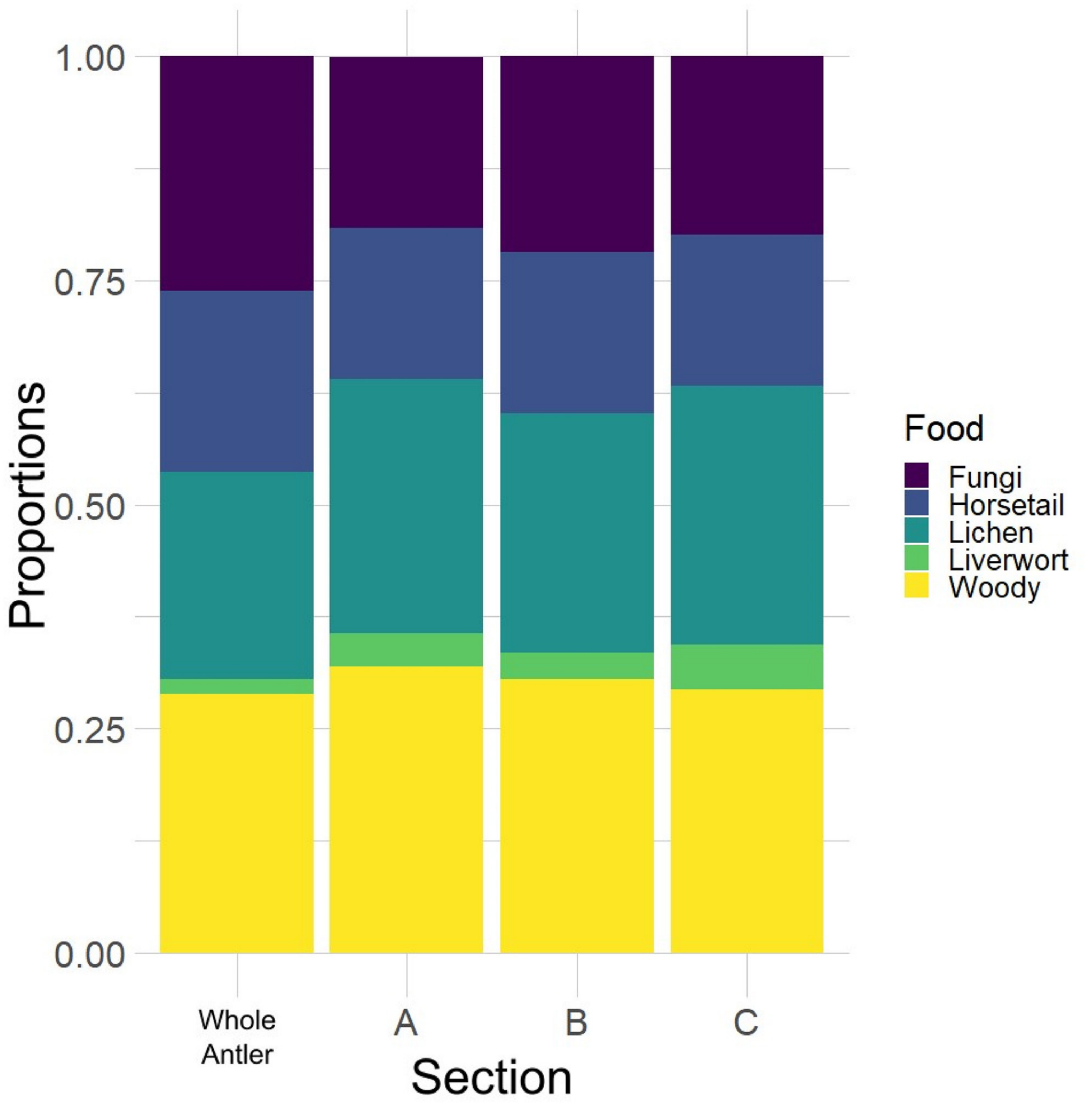
Dietary proportions estimated from all antlers with all effects set to NULL. Diet proportions were computed for whole antler, and the lower (A), the middle (B), and the upper (C) antler segment.

Estimated dietary inputs from antler tissue showed large degrees of uncertainty (i.e., large 95% credible intervals) (Table S7; Figures 5 and 6). Overall, BSIMMs predicted consistent diets throughout the period of antler growth when averaged among antlers, despite observable increases in δ^15^N. Estimated diets given uninformative priors showed a diet consisting of primarily liverwort with a decrease in lichen and increase in fungi from pedicle to tip (Figure 5a,d; Table S7). The addition of informative priors based on rumen contents from culled individuals produces estimates of dietary inputs that, more closely reflect the known diets of Qamanirjuaq caribou (Figure 5e,h). Informed dietary mixing models estimated a dietary mix of horsetail, lichen, and woody plants with a slight increase in horsetail and lichen from pedicle to tip (Figure 5e,h; Table S7). Estimated dietary shifts during antler growth are subtle because they are averaged across all 12 individuals.

Mixing models were compared using LOOIC. Models of diets using whole antler tissue with uninformative priors and antler length as a continuous covariate produced the best fit (Table S8). Despite, differences in model fit, individuals had similar dietary mixes regardless of whether they were modeled using uninformative or informative priors. For example, CMN39090 and CMN39151 showed almost no change in dietary proportion estimates between the uninformed and informed mixing models, with a relatively even mix of horsetail, lichen, and woody plants throughout antler growth (Figure S3).

### 3.4 Antler segments

Male Qamanirjuaq caribou used in this study begin antler growth in February and end antler growth before the rut in September. BSIMMs with uninformative priors for antlers divided into segments to approximate the seasons, on average, produced similar results to BSIMMs using antler length as a continuous effect and informative priors. Models with uninformative priors and no effects (i.e., no random, nested, or continuous effects) provided the best fit (Table S8). Both model types estimated mixed diets of fungi, horsetail, lichen, and woody plants with little to no liverwort (Figure 6). Across all antlers, dietary input estimates differed very little between the whole antler and lower, middle, and upper antler segments – with a large standard deviation for each food source (Figure 6).

While averaging across all antlers did not indicate dietary shifts throughout antler growth, with the three antler segments suggesting no seasonal dietary shifts, some individuals showed slight changes (Figure 4). Estimates for winter and early spring diets using the lower third of antler tissue (antler growth from February to April) generally suggested a diet of more lichen and woody plants (Figure 4). Model estimates for the summer diet using the middle third of antler tissue (antler growth during May to July) predicted diets very similar to the lower antler segment with some increases in fungi. Finally, fall diets modeled using the upper third of antler tissue (antler growth for August and September) were estimated to have contained higher contents of fungi and horsetail (Figure 4). Broadly, the dietary input estimates are consistent with observed increases in δ^15^N_col_.

In summary, changes in isotopic compositions within antlers varied among individuals, with many showing unique trends for δ^13^C_col_ and δ^15^N_col_. Despite antler isotopic variation, predicted diets during antler growth remained consistent. Only when diets were modeled for antlers divided into segments (i.e., lower, middle, and upper segments) did seasonal dietary shifts become observable (Figure S5 for BSIMM matrix plots).

## 4 DISCUSSION

Variations in δ^13^C within and among food source functional groups sampled for this study are aligned with values reported in the literature for similar species (Fletcher et al., 2006; Högberg et al., 1999; Máguas & Brugnoli, 1996; Marshall et al., 2007; Tahmasebi et al., 2017). Woody plants were composed of vascular C_3_ plants such as conifers, birch, and shrubs based on their presence in rumen content samples (Miller, 1976). Values of δ^13^C among woody plants are quite varied but generally lower than the other food sources (Tables 1 and 2; Figure 1). High isotopic variation among woody plant taxa likely results from differences in water‐use efficiency, with more drought‐tolerant plants having higher δ^13^C (Marshall et al., 2007). The degree of stomatal closure also differs among woody plants; drought‐tolerant plants show lower degrees of stomatal closure (Marshall et al., 2007). Previous studies have found that Arctic woody plants undergo partial stomatal closure (e.g., willows, birch, and shrubs), reduced stomatal conductance, and a decline in CO_2_ diffusion across leaves, thus leading to enrichment in ^13^C compared to similar species in less arid environments such as the boreal forest (Aerts et al., 2009; Kristensen et al., 2011; Marshall et al., 2007; Welker et al., 1993). The woody samples from our food sources show a similar trend. The degree of variation within arctic and boreal forest groups, however, made for insignificant differences, and a larger woody plant data set may be required to parse out the subtle variations between the two ecozones (Table 2).

Bryophytes (i.e., liverworts) had the lowest δ^13^C, likely because their lack of stomata allows for greater CO_2_ diffusion, creating an isotopic fractionation without carbon‐concentrating mechanisms (Fletcher et al., 2006; Laws et al., 1995; Popp et al., 1998). The *Cladonia* genus of lichen (commonly referred to as reindeer‐lichen, which made up a bulk of sampled lichen) is part of the photobiont group of lichens, meaning they have a symbiotic algal partnership with phycobiont of green algae (Cardinale et al., 2008; Máguas & Brugnoli, 1996). Photobiont lichens have rapid water vapor uptake, allowing maximal photosynthetic output in shorter periods of time, ideal for the Arctic tundra environment (Lange et al., 1986). The rapid photosynthetic output decreases carbon isotope discrimination, resulting in higher δ^13^C than other food sources such as liverwort and woody plants (Fletcher et al., 2006). Ectomycorrhizal fungi used in this study are 1–3‰ higher in δ^13^C than their host plants (i.e., the woody plants in this study), due to an isotopic fractionation that occurs during the biochemical process of transferring carbon from the host plant to the fungus (Högberg et al., 1999; Pate & Arthur, 1998). Lastly, Equisetaceae (i.e., horsetails in this study) are non‐mycorrhizal plants but have a unique relationship with fungal root endophytes, which leads to an enrichment in ^13^C and similar isotopic compositions to those of fungi (Giesemann et al., 2020).

Caribou bone and antler tissues showed similar δ^13^C_col_ but differed in δ^15^N_col_ (Figure 3). Bone and antler tissue is composed of protein collagen and bioapatite; however, unlike bone tissue, antler does not undergo secondary ossification (Chen et al., 2009; Stevens & O'Connell, 2016). Bone tissue slowly remodels over the course of ~10 years in larger mammals; as a result, the δ^13^C_col_ and δ^15^N_col_ reflect the average ~10‐year diet (Hedges et al., 2007). Thus, offsets in δ^13^C_col_ and δ^15^N_col_ between bone and antler are likely caused by the different metabolic processes that occur during tissue formation as well as seasonal dietary factors not recorded by antlers (Stevens & O'Connell, 2016). While δ^13^C_col_ values are similar between bone and whole antler, bone δ^13^C_col_ appears most similar to the base of the antler (Figure 3), suggesting enhanced bone remodeling during the spring months, rather than simple averaging of the δ^13^C of food resources year‐round. Lower bone δ^15^N_col_ than that of whole antler (Figure 3) also suggests that bone remodeling occurs during seasons where antler growth does not occur. However, the δ^15^N_col_ of bone still bears greatest similarity to the antler base (Figure 3).

Qamanirjuaq caribou are known to change their diets during migration, as indicated by a switch from lichen to rush‐like plants (i.e., Equisetaceae) in the rumens of culled individuals (Miller, 1976). We therefore predicted, based on the isotopic differences among food source functional groups (Table 1; Figure 1), that coordinated seasonal dietary shifts would lead to (i) decreases in δ^13^C_col_ along antler length and (ii) increases in δ^15^N_col_ along antler length.

Seasonal shifts from lichen to *Equisetaceae* should produce a decrease in δ^13^C of ~0.5‰ (Table 1; Figure 1), which was not apparent for all antlers. We found isotopic variation within individual antlers of a similar or smaller magnitude (<0.5 V Figure 2; Figure S2) but there was no consistent decrease in δ^13^C_col_. Such small, individualized variations in δ^13^C_col_ may result from: (i) a lack of coordinated seasonal shifts in diet (i.e., individualized dietary shifts), (ii) little spatiotemporal variation in δ^13^C of Arctic flora along the migratory route, or (iii) concurrent physiological and dietary changes that balance out to produce little change in δ^13^C_col_. Caribou are traditionally considered generalists meaning they demonstrate flexible foraging behaviors and dietary switching, depending on plant biomass availability (Barboza et al., 2018; Denryter et al., 2017; Thompson et al., 2015; Van Der Wal et al., 2000). This behavior is reflected in changes in rumen contents among Qamanirjuaq caribou over the antler growth season (Miller, 1976). Given that the males selected for this study were all culled during September 1967 in similar locations, we initially assumed that they would all have had similar diets and show coordinated seasonal dietary shifts. Instead, we observed individualized patterns of δ^13^C_col_ change among antlers, suggesting that individual caribou may vary their diets in uncoordinated ways. Considered together, the changes in δ^13^C_col_ do not suggest coordinated shifts from lichen to Equisetaceae (Miller, 1976). Caribou are known to consume a wide range of plants and to selectively forage from their surrounding plant community to optimize their nutrient intake (Denryter et al., 2017). The male spring migration route traverses areas comprised of diverse plant communities, including lowland meadows, muskegs, pine‐dominated forests, and mixed forests (Miller, 1976). Thus, individualized average diets and dietary shifts may reflect differences in selectiveness among individuals. The considerable overlap in δ^13^C among lichens, grass‐like plants, and fungi suggests that dietary change may also be difficult to detect based on δ^13^C_col_ and, given the high degree of variation within a plant functional group, may mask coordinated dietary shifts.

Similarly, δ^13^C_col_ does not appear to track the movement of Qamanirjuaq caribou from boreal forest to tundra during their spring migration (Miller, 1976). The sampled individuals, having been collected in similar locations at similar times, should have followed very similar migration tracks (Parker, 1972). Plants typically show considerable spatiotemporal variation in δ^13^C; flora from closed canopy forests show comparatively low δ^13^C, increasing with water availability and temperature (Smedley et al., 1991; Szpak et al., 2010; van der Merwe & Medina, 1991). We find no significant difference in δ^13^C between sampled plants from the two major ecozones traversed by the Qamanirjuaq, however (Figure 1). Taken together, δ^13^C_col_ values from antlers do not appear to reflect climate differences along caribou migration tracks. Decreases in δ^13^C_col_ due to dietary shifts or movement among ecozones (i.e., boreal forest to tundra) may therefore be masked by physiological effects such as stress, which has been shown to increase δ^13^C_col_ (Hatch, 2012). Interestingly, bioapatite from the analyzed subset of four antlers showed a consistent increase of between 0.5 and 1.0‰ in δ^13^C_CO3_ along the antler length. Values of δ^13^C_CO3_ reflect the whole diet as opposed to protein, which is the case for δ^13^C_col_. This result suggests that dietary or nutritional changes may affect the δ^13^C of non‐protein versus protein components of caribou diets differently (Lee‐Thorp et al., 1989).

We also predicted, based on the isotopic differences among plant functional groups (Tables 1 and 2; Figure 1), that coordinated seasonal dietary shifts would lead to increases in δ^15^N, and indeed δ^15^N_col_ increased from the pedicle to tip for all sampled antlers by ~1–3‰ (Figure 2). As for δ^13^C_col_, however, there was a high degree of variation among individuals. Increasing and variable patterns of δ^15^N_col_ change could suggest (i) climatological effects acting on plants from the different ecozones and among seasons, (ii) coordinated, seasonal dietary changes primarily affecting δ^15^N_col_, and/or (iii) responses to physiological stress. Relevant to the known migration patterns of Qamanirjuaq caribou, plants in drier environments tend to show higher foliar δ^15^N because moisture content has a major impact on δ^15^N, causing it to increase with latitude in this setting (Handley et al., 1999). Higher moisture content increases leaching and nitrogen transformation in the soil creating an overall enrichment in soil ^15^N (Handley et al., 1999; Murphy & Bowman, 2006; Schuur & Matson, 2001). Our samples, however, do not show differences in δ^15^N when comparing food sources between the southern (spring) and northern (fall) ranges of male Qamanirjuaq caribou (Tables 1 and 2; Figure 1). Thus, migrating between boreal forest and Arctic tundra may not explain changes in δ^15^N_col_ from antlers in the absence of significant dietary change. Increases in δ^15^N_col_ from antlers are therefore more likely a result of several factors impacting antler tissue during the growth season, including dietary and physiological stress.

Tundra plants tend to have a wider range of δ^15^N, as the nutrient‐poor ecosystem creates competitive partitioning of nitrogen (Nadelhoffer et al., 1996). Different nitrogen uptake methods (i.e., root depth and mycorrhizal association) in tundra plants also create a wide degree of variation in δ^15^N values compared to similar plant species in other terrestrial ecosystems (Aerts et al., 2009; Dalerum & Angerbjörn, 2005; Michelsen et al., 1998; Nadelhoffer et al., 1996). Qamanirjuaq caribou are known to switch from lower δ^15^N sources like woody plants and lichen in the winter and spring, which can persist in colder parts of the year when moisture availability is low, to higher δ^15^N food sources like horsetail and fungi in the summer, which thrive in high moisture soils and moderate weather conditions (Kristensen et al., 2011; Michelsen et al., 1998). Increasing δ^15^N could be partially explained by such a dietary change, though the magnitude of change is far less than expected (Figures 5 and 6; Figures S3 and S4), additionally, results of our mixing models suggest caribou retain a mixed diet throughout antler growth.

An alternative explanation for δ^15^N_col_ increases along antlers is physiological stress, which can drive 1–3‰ increases in δ^15^N_col_ due to metabolization of the body's tissues (Ambrose, 1991; Hobson & Clark, 1992; Kelly, 2000). Enrichment in ^15^N can be particularly strong in ruminants, as they can recycle nitrogen in the rumen using urea in microbial digestion, creating additional separation of ^14^N and ^15^N (Ambrose, 1991; Kelly, 2000; Sealy et al., 1987). Antler growth occurs throughout the spring and summer months when nutritional food resources are more abundant (male's antler tissue development begins in February and ends before the rut in September) (Parker, 1972; Parker et al., 2005). Throughout spring and summer, male caribou can rely on the abundance of nutritional food sources to replenish the 23% loss in body proteins that occurs post‐rut and throughout the winter (Barboza et al., 2004). Antler growth in males, however, is costly, with energy requirements increasing by 8% to 16% during growth (Moen & Pastor, 1998). While the protein requirements for the energy increase are met, resources like calcium and phosphorus are reabsorbed from bone tissue to aid in antler development (Moen & Pastor, 1998). Energy requirements and calcium phosphorus reabsorption reach their peak shortly before antler development ends in September, correlating with the observed δ^15^N_col_ increase (Moen & Pastor, 1998). It is possible that during this period of rapid tissue development and increased energy requirements, there is an increase in nitrogen recycling within the male caribou. Thus, both seasonal dietary change and the stress of antler growth may contribute to increasing antler δ^15^N_col_.

To further test for coordinated seasonal dietary shifts, we used Bayesian Stable Isotope Mixing Models (BSIMMs), with and without informative priors. BSIMMs did not estimate statistically significant dietary shifts for any individual, potentially due to significant compositional overlap of the Arctic flora in nitrogen versus carbon isotope space (Figure 1). BSIMMs for some sampled Qamanirjuaq caribou indicated non‐significant decreases in lower δ^15^N rich plants like liverwort and woody plants and increases in higher δ^15^N food sources like fungi and horsetail (Figures S3 and S4), as seen in rumen contents (Miller, 1976). The majority of BSIMM results, however, suggested relatively consistent diets throughout antler growth (Figures 5 and 6). This implies that Qamanirjuaq male caribou retain their diet possibly based on its nutritional quality (Denryter et al., 2017, 2022; Miller, 1976; Parker, 1972). The non‐significant changes in diet found in some BSIMM models and seen in rumen content could be indicative of an opportunistic feeding strategy, switching to different plant groups based on forage availability (Kaluskar et al., 2020; Miller, 1976; Parker, 1972). Different feeding behaviors may limit the amount of intraspecific resource competition among males during periods of nutrient limitation such as during the summer months (Couturier et al., 2009; Webber et al., 2022).

## 5 CONCLUSIONS

As in previous studies of red deer (*Cervus elaphus*), Qamanirjuaq caribou bone and antler tissue are not isotopically equivalent, which is likely a result of differing tissue formation processes and dietary factors (Stevens & O'Connell, 2016). Furthermore, antler δ^13^C_col_ did not show predicted decreases with continued growth; antler δ^13^C_col_ varied within an antler and among individuals. δ^13^C_CO3_, however, did increase along the antler length in the subset of individuals analyzed, potentially indicating a dietary or nutritional change that affects the δ^13^C of non‐protein versus protein components. Values of δ^15^N_col_ increased as predicted, consistent with either coordinated seasonal dietary changes from lichen to rush‐like plants during spring migration and/or the stress of increased energy requirements during antler tissue development. It remains difficult, however, to accurately model dietary shifts for caribou, given the considerable overlap of Arctic flora in stable nitrogen versus carbon isotope space. Future analysis of antler tissue should incorporate a wider array of isotopes: (i) bioapatite δ^18^O_CO3_ and δ^18^O_phosphate_ can be used to provide information regarding temporal changes during antler tissue development, (ii) bioapatite ^87^Sr/^86^Sr may be used to provide information regarding migration routes, and (iii) collagen δ^2^H and δ^34^S, diet indicators, may provide further isotopic separation among isotopically similar food sources. New investigations of isotopic systems in caribou antlers will further detail how this tissue can be used as an indicator for both seasonal migration and diet. Once the controlling factors are fully understood, antlers will become an important archive of ecological data for studying both short‐ and long‐term changes in caribou biology.

## AUTHOR CONTRIBUTIONS

**Matthew Brenning:** Conceptualization (equal); formal analysis (equal); methodology (equal); software (lead); writing – original draft (lead). **Fred J. Longstaffe:** Methodology (equal); resources (equal); writing – review and editing (equal). **Danielle Fraser:** Conceptualization (equal); funding acquisition (equal); methodology (equal); resources (equal); supervision (equal); writing – review and editing (equal).

## FUNDING INFORMATION

Funding was provided by the Natural Sciences and Engineering Research Council of Canada (NSERC) Grant no: RGPIN‐2018‐05305 given to Danielle Fraser. Funding from NSERC Grant no: (RGPIN 2019‐05904) and Canada Research Chairs Grant no: (X1277C01), both to Fred J Longstaffe, also helped to sustain instrumentation and staff at Western's Laboratory for Stable Isotope Science during this research.

## Supporting information


Appendix S1

Appendix S2

## Data Availability

The datasets used and RStudio code used in this study are available with the corresponding supplementary material.
