# Supplementary material for: Variation in stable carbon (δ 13C) and nitrogen (δ15N) isotope compositions along antlers of Qamanirjuaq caribou (Rangifer tarandus groenlandicus)
Source: Ecol Evol. 2024 Mar 18;14(3):e11006. doi: 10.1002/ece3.11006 (PMC10945312; doi:10.1002/ece3.11006)
Supplement: Supplementary file 1 — Appendix S1 [file ECE3-14-e11006-s002.docx]

Ecology and Evolution

**Variation in stable carbon (*δ*^13^C) and nitrogen (*δ*^15^N) isotope compositions along antlers of Qamanirjuaq caribou (*Rangifer tarandus groenlandicus*)**

Matthew Brenning^1,2*^, Fred J Longstaffe^3^, Danielle Fraser^1,2,4,5^

^1^Department of Earth Sciences, Carleton University, 1125 Colonel By Drive, Ottawa, Ontario K1S 5B6, Canada.

^2^Palaeobiology, Canadian Museum of Nature, PO Box 3443 Stn “D”, Ottawa ON K1P 6P4.

^3^Department of Earth Sciences, The University of Western Ontario, 1151 Richmond St, London, Ontario N6A 3K7

^4^Department of Biology, Carleton University, 1125 Colonel By Drive, Ottawa, Ontario K1S 5B6, Canada.

^5^Department of Paleobiology, Smithsonian National Museum of Natural History, Washington, DC, 20560, USA.

Matthewbrenning@cmail.carleton.ca

**Supplementary Table 1.** Data from the Canadian Wildlife Services study for the 12 male caribou selected for study

| CMN Catalogue Number | Date Collected | Latitude | Longitude | Age (M) | Weight (lbs) | Total Length (cm) | Tail (cm) | Hind Foot (cm) | Ear (cm) |
| --- | --- | --- | --- | --- | --- | --- | --- | --- | --- |
| CMNMA 39079 | 1967-09-17 | 61.15 | -95.4167 | 39 | 218 | 172 | 12 | 49 | 12 |
| CMNMA 39090 | 1967-09-18 | 61.25 | -95.35 | 59 | 283 | 188 | 14.5 | 56 | 12.5 |
| CMNMA 39102 | 1967-09-18 | 61.25 | -95.35 | 51 | 258 | 180 | 14.5 | 53 | 12 |
| CMNMA 39104 | 1967-09-18 | 61.25 | -95.35 | 54 | 277 | 178 | 12.5 | 57 | 11.5 |
| CMNMA 39120 | 1967-09-19 | 61.25 | -95.3167 | 39 | 241 | 168 | 12 | 53 | 12 |
| CMNMA 39121 | 1967-09-19 | 61.25 | -95.3167 | 51 | 221 | 169 | 17 | 55 | 13.5 |
| CMNMA 39132 | 1967-09-19 | 61.25 | -95.3167 | 51 | 260 | 185 | 16 | 54.5 | 11 |
| CMNMA 39145 | 1967-09-21 | 61.2 | -95.25 | 51 | 288 | 187 | 14 | 56.5 | 13 |
| CMNMA 39148 | 1967-09-21 | 61.2 | -95.25 | 51 | 274 | 182.5 | 14 | 56.5 | 12 |
| CMNMA 39149 | 1967-09-21 | 61.2 | -95.25 | 51 | 283 | 175 | 14.5 | 53 | 13 |
| CMNMA 39151 | 1967-09-21 | 61.2 | -95.25 | 51 | 281 | 182 | 16 | 56.5 | 13 |
| CMNMA 39107 | 1967-09-19 | 61.21667 | -95.0833 | 51 | 268 | 176 | 10.5 | 53.5 | 12 |
| CMNMA 39108 | 1967-09-19 | 61.21667 | -95.0833 | 51 | 270 | 180 | 12 | 53 | 12 |
| CMNMA 39110 | 1967-09-19 | 61.21667 | -95.0833 | 39 | 284 | 182 | 10 | 52 | 11 |
| CMNMA 39136 | 1967-09-21 | 61.21667 | -95.0833 | 51 | 342 | 182 | 11 | 54 | 13 |

**Supplementary Table 2.** Stable carbon (*δ*^13^C) and nitrogen (*δ*^15^N) isotope data for source groups (Horsetail, Lichen, liverwort, and woody plants), and location of collection by the National Herbarium of Canada

|  | | ***δ*^13^C** | ***δ*^15^N** |  |  |  |
| --- | --- | --- | --- | --- | --- | --- |
| **Species** | **CMN#** | **(VPDB, ‰)** | **(AIR, ‰)** | **Group** | **Lat** | **Long** |
| *Equisetum arvense* | CAN 10004683 | –24.07 | +3.64 | Horsetail | 63.83342 | -104.084 |
| *Equisetum scirpoides* | CANL 10004537 | –27.8 | +1.8 | Horsetail | 64.15 | -102.533 |
| *Equisetum variegatum* | CANL 10001236 | –25.6 | +1.0 | Horsetail | 64.15 | -103.533 |
| *Equisetum variegatum* | CAN 10001224 | –25.36 | +10.88 | Horsetail | 64.31667 | -96.05 |
| *Equisetum variegatum* | CAN 10001221 | –26.73 | +9.54 | Horsetail | 64.31667 | -96.05 |
| *Equisetum arvense* | CAN 10004684 | –24.81 | +2.33 | Horsetail | 63.65 | -104.5 |
| *Equisetum arvense* | CAN 10004749 | –23.74 | +3.81 | Horsetail | 63.83333 | -104.083 |
| *Equisetum sylvaticum* | CAN 10004832 | –23.57 | +2.99 | Horsetail | 63.83333 | -104.083 |
| *Equisetum sylvaticum* | CAN 10004827 | –23.36 | +5.03 | Horsetail | 63.83333 | -104.083 |
| *Cladonia amaurocraea* | CANL 89317 | –23.7 | –2.9 | Lichen | 59.45 | -97.7167 |
| *Cladonia amaurocraea* | CANL 89230 | –24.39 | +1.81 | Lichen | 62.21667 | -94.9833 |
| *Cladonia pocillum* | CANL 89231 | –25.04 | –0.71 | Lichen | 62.21667 | -94.9833 |
| *Cladonia rangiferina* | CANL 31444 | –24.64 | –0.70 | Lichen | 64.21667 | -102.117 |
| *Cladonia subfurcata* | CANL 89232 | –22.49 | –0.81 | Lichen | 62.21667 | -94.9833 |
| *Cladonia amaurocraea* | CANL 46908 | –24.1 | +0.5 | Lichen | 63.58333 | -110.033 |
| *Cladonia amaurocraea* | CANL 46908 QCD | –23.8 | –1.8 | Lichen | 63.58333 | -110.033 |
| *Cladonia amaurocraea* | CANL 46938 | –27.27 | –2.46 | Lichen | 60.78333 | -107.633 |
| *Cladonia arbuscula* | CANL 89268 | –25.08 | –0.27 | Lichen | 59.08333 | -102.083 |
| *Cladonia coccifera* | CANL 46910 | –24.82 | 0.32 | Lichen | 63.58333 | -110.033 |
| *Cladonia rangiferina* | CANL 31427 | –24.1 | –0.7 | Lichen | 63.7 | -104.417 |
| *Cladonia stellaris* | CANL 46907 | –23.57 | –0.44 | Lichen | 63.58333 | -110.033 |
| *Cladonia amaurocraea* | CANL 46952 | –23.9 | –0.8 | Lichen | 61.51667 | -108.1 |
| *Cladonia amaurocraea* | CANL 46923 | –24.93 | –1.25 | Lichen | 62.58333 | -109.633 |
| *Cladonia amaurocraea* | CANL 46890 | –24.42 | –0.82 | Lichen | 62.53333 | -108.3 |
| *Cladonia cenotea* | CANL 89249 | –27.59 | –1.44 | Lichen | 59.08333 | -102.083 |
| *Cladonia chlorophaea* | CANL 89472 | –26.92 | +1.34 | Lichen | 59.08333 | -102.083 |
| *Cladonia coccifera* | CANL 46953 | –24.39 | +0.15 | Lichen | 61.51667 | -108.1 |
| *Cladonia coccifera* | CANL 89318 | –24.80 | +1.63 | Lichen | 59.45 | -97.7167 |
| *Cladonia coccifera* | CANL 46892 | –24.48 | +0.54 | Lichen | 60.28333 | -109.033 |
| *Cladonia coccifera* | CANL 89238 | –24.51 | +0.52 | Lichen | 60.08333 | -103.333 |
| *Cladonia cornuta* | CANL 46954 | –25.39 | +1.51 | Lichen | 61.51667 | -108.1 |
| *Cladonia crispata* | CANL 46933 | –24.27 |  | Lichen | 60.78333 | -107.633 |
| *Cladonia crispata* | CANL 89808 | –24.30 | –0.46 | Lichen | 59.75417 | -103.333 |
| *Cladonia cristatella* | CANL 89250 | –22.93 | –1.84 | Lichen | 59.08333 | -102.083 |
| *Cladonia deformis* | CANL 46955 | –25.01 | +1.61 | Lichen | 61.51667 | -108.1 |
| *Cladonia deformis* | CANL 46919 | –26.11 | –1.29 | Lichen | 62.58333 | -109.633 |
| *Cladonia gracilis* | CANL 46891 | –23.85 | –2.81 | Lichen | 60.28333 | -109.033 |
| *Cladonia gracilis* | CANL 46927 | –21.31 | –2.76 | Lichen | 62.58333 | -109.633 |
| *Cladonia gracilis* | CANL 46934 | –25.08 | –3.92 | Lichen | 60.78333 | -107.633 |
| *Cladonia macrophylla* | CANL 89297 | –25.17 | +1.89 | Lichen | 59.55 | -97.8 |
| *Cladonia macrophylla* | CANL 89239 | –25.00 | +0.90 | Lichen | 60.08333 | -103.333 |
| *Cladonia phyllophora* | CANL 89807 | –24.05 | +2.43 | Lichen | 59.75417 | -103.333 |
| *Cladonia phyllophora* | CANL 89474 | –22.58 | +1.56 | Lichen | 59.45 | -97.7333 |
| *Cladonia rangiferina* | CANL 46928 | –23.9 | +0.4 | Lichen | 61.51667 | -108.1 |
| *Cladonia rangiferina* | CANL 46940 | –25.03 | –3.62 | Lichen | 60.78333 | -107.633 |
| *Cladonia rangiferina* | CANL 46925 | –24.1 | –6.0 | Lichen | 62.58333 | -109.633 |
| *Cladonia rangiferina* | CANL 89245 | –24.64 | +1.06 | Lichen | 59.08333 | -102.083 |
| *Cladonia rangiferina* | CANL 46924 | –25.13 | –1.32 | Lichen | 62.58333 | -109.633 |
| *Cladonia stellaris* | CANL 89247 | –24.1 | –4.1 | Lichen | 59.08333 | -102.083 |
| *Cladonia stellaris* | CANL 46956 | –24.8 | –3.7 | Lichen | 61.51667 | -108.1 |
| *Cladonia stellaris* | CANL 46926 | –26.31 | –0.95 | Lichen | 62.58333 | -109.633 |
| *Cladonia stellaris* | CANL 46939 | –24.76 | –1.46 | Lichen | 60.78333 | -107.633 |
| *Cladonia subfurcata* | CANL 89319 | –23.3 | –3.5 | Lichen | 59.45 | -97.7167 |
| *Cladonia subfurcata* | CANL 89319 QCD | –23.3 | –3.2 | Lichen | 59.45 | -97.7167 |
| *Cladonia symphycarpa* | CANL 89809 | –25.41 | +1.12 | Lichen | 59.75417 | -103.333 |
| *Ptilidium ciliare* | CANL 13492 | –27.6 | –5.0 | Liverwort | 62.58333 | -109.633 |
| *Ptilidium ciliare* | CANL 21717 | –29.6 | –1.5 | Liverwort | 59.08333 | -102.083 |
| *Ptilidium ciliare* | CANL 25511 | –28.8 | +0.2 | Liverwort | 60.91667 | -110.5 |
| *Ptilidium ciliare* | CANL 13491 | –28.2 | –4.6 | Liverwort | 62.58333 | -109.633 |
| *Larix laricina* | CAN 10005691 | –25.58 | –1.95 | Woody | 64.13333 | -102.533 |
| *Larix laricina* | CANL 10005686 | –28.2 | –3.0 | Woody | 64.13333 | -102.533 |
| *Picea glauca* | CAN 10005822 | –26.12 | –7.79 | Woody | 64.21667 | -102.117 |
| *Picea glauca* | CANL 10005826 | –26.2 | –3.0 | Woody | 63.63333 | -104.65 |
| *Picea glauca* | CAN 10005807 | –27.43 | –3.57 | Woody | 63.95 | -103.883 |
| *Picea mariana* | CANL 10006171 | –26.5 | –6.1 | Woody | 63.63333 | -104.65 |
| *Picea mariana* | CAN 10006162 | –26.74 | –6.17 | Woody | 63.61667 | -104.517 |
| *Picea mariana* | CANL 10006163 | –28.3 | –7.9 | Woody | 63.63333 | -104.65 |
| *Rhododendron groenlandicum* | CAN 10076271 | –25.61 | –1.33 | Woody | 64.13333 | -102.533 |
| *Rhododendron tomentosum* | CANL 10076538 | –27.6 | –5.4 | Woody | 64.21667 | -102.117 |
| *Vaccinium uliginosum* | CAN 10078701 | –26.04 | –1.56 | Woody | 64.18333 | -103.683 |
| *Rhododendron groenlandicum* | CANL 10076270 | –28.6 | –2.4 | Woody | 63.95 | -103.883 |
| *Rhododendron groenlandicum* | CANL 10076268 | –27.2 | –7.6 | Woody | 63.63333 | -104.683 |
| *Rhododendron tomentosum* | CANL 10076539 | –27.9 | –3.0 | Woody | 63.95 | -103.883 |
| *Rhododendron tomentosum* | CANL 10076531 | –27.3 | +1.4 | Woody | 63.61667 | -104.517 |
| *Rhododendron tomentosum* | CANL 10075017 | –27.7 | –3.2 | Woody | 63.63333 | -104.65 |
| *Rhododendron tomentosum* | CAN 10076543 | –26.27 | –4.48 | Woody | 63.63333 | -104.683 |
| *Betula glandulosa* | CAN 10026784 | –24.89 | +0.21 | Woody | 63.65 | -104.5 |
| *Betula glandulosa* | CAN 10026782 | –25.24 | –1.71 | Woody | 63.95 | -103.883 |
| *Betula glandulosa* | CAN 10026785 | –26.12 | –0.81 | Woody | 63.95 | -103.883 |
| *Betula glandulosa* | CAN 10026717 | –24.87 | –0.06 | Woody | 63.65 | -104.5 |

**Supplementary Table 3.** Stable carbon (*δ*^13^C) and nitrogen (*δ*^15^N) isotope data for fungi, as reported by Hobbie *et al.* (2017).

| **Fungi** | | | |
| --- | --- | --- | --- |
|  | | ***δ*^13^C** | ***δ*^15^N** |
| **Location** | **Taxon** | **(VPDB, ‰)** | **(AIR, ‰)** |
| Atigun | *Boletaceae* | –25.3 | +8.7 |
| Atigun | *Boletus* | –24.9 | +9.8 |
| Atigun | *Leccinum* | –25.0 | +6.6 |
| Atigun | *Laccaria* | –25.2 | +2.8 |
| Atigun | *Lactarius* | –25.7 | +4.9 |
| Atigun | *Russula* | –25.6 | +5.8 |
| Toolik Lake | *Boletaceae* | –25.5 | +6.3 |
| Toolik Lake | *Bpletus* | –22.7 | +2.0 |
| Toolik Lake | *Leccinum* | –25.4 | +6.5 |
| Toolik Lake | *Laccaria* | –25.3 | +1.3 |
| Toolik Lake | *Lactarius* | –25.4 | +3.0 |
| Toolik Lake | *Russula* | –26.8 | +4.9 |

**Supplementary Table 4A.** Stable carbon (*δ*^13^C_CO3_) isotope data for structural carbonate obtained from bioapatite for sample CMN39107. The suffix A in the sample name indicates subsamples collected along the length of the antler, B = subsamples collected along the mandible.

|  | | ***δ*^13^C_CO3_** |
| --- | --- | --- |
| **Num** | **Sample** | **(VPDB, ‰)** |
| 1 | 39107-1A | –8.63 |
| 2 | 39107-2A | –8.77 |
| 3 | 39107-3A | –8.84 |
| 4 | 39107-4A | –8.95 |
| 5 | 39107-5A | –8.93 |
| 6 | 39107-6A | –8.83 |
| 7 | 39107-6A dup | –8.65 |
| 8 | 39107-7A | –8.53 |
| 9 | 39107-8A | –8.67 |
| 10 | 39107-9A | –8.83 |
| 11 | 39107-10A | –8.57 |
| 12 | 39107-11A | –8.69 |
| 13 | 39107-12A | –8.76 |
| 14 | 39107-13A | –8.65 |
| 15 | 39107-14A | –8.61 |
| 16 | 39107-15A | –8.63 |
| 17 | 39107-16A | –8.72 |
| 18 | 39107-17A | –8.76 |
| 19 | 39107-18A | –8.49 |
| 20 | 39107-19A | –8.79 |
| 21 | 39107-20A | –8.88 |
| 22 | 39107-21A | –9.00 |
| 23 | 39107-22A | –8.73 |
| 24 | 39107-22A dup | –8.49 |
| 25 | 39107-23A | –8.92 |
| 26 | 39107-24A | –8.71 |
| 27 | 39107-25A | –8.34 |
| 28 | 39107-26A | –8.52 |
| 29 | 39107-27A | –8.37 |
| 30 | 39107-28A | –7.93 |
| 31 | 39107-29A | –7.98 |
| 32 | 39107-30A | –8.23 |
| 33 | 39107-1B | –9.28 |
| 34 | 39107-1B dup | –9.24 |

**Supplementary Table 4B.** Stable carbon (*δ*^13^C_CO3_) isotope data for structural carbonate obtained from bioapatite for sample CMN39108. The suffix A in the sample name indicates subsamples collected along the length of the antler.

|  | | ***δ*^13^C_CO3_** |
| --- | --- | --- |
| **Num** | **Sample** | **(VPDB, ‰)** |
| 35 | 39108-1A | –8.95 |
| 36 | 39108-2A | –7.88 |
| 37 | 39108-2A | –8.79 |
| 38 | 39108-2A dup | –8.10 |
| 39 | 39108-4A | –8.39 |
| 40 | 39108-5A | –8.38 |
| 41 | 39108-5A dup | –8.20 |
| 42 | 39108-6A | –7.55 |
| 43 | 39108-7A | –7.81 |
| 44 | 39108-7A dup | –7.52 |
| 45 | 39108-8A | –7.83 |
| 46 | 39108-23A | –7.44 |
| 47 | 39108-24A | –7.54 |
| 48 | 39108-25A | –7.32 |
| 49 | 39108-25A dup | –7.22 |

**Supplementary Table 4C.** Stable carbon (*δ*^13^C_CO3_) isotope data for structural carbonate obtained from bioapatite for sample CMN39145. The suffix A in the sample name indicates subsamples collected along the length of the antler, B = subsamples collected along the mandible.

|  | | ***δ*^13^C_CO3_** |
| --- | --- | --- |
| **Num** | **Sample** | **(VPDB, ‰)** |
| 50 | 39145-2A | –8.26 |
| 51 | 39145-2A dup | –8.18 |
| 52 | 39145-3A | –8.14 |
| 53 | 39145-4A | –8.25 |
| 54 | 39145-5A | –8.19 |
| 55 | 39145-6A | –8.06 |
| 56 | 39145-7A | –8.28 |
| 57 | 39145-8A | –8.38 |
| 58 | 39145-9A | –8.25 |
| 59 | 39145-10A | –8.20 |
| 60 | 39145-10A dup | –8.26 |
| 61 | 39145-11A | –7.99 |
| 62 | 39145-12A | –7.99 |
| 63 | 39145-13A | –7.96 |
| 64 | 39145-14A | –8.19 |
| 65 | 39145-15A | –8.09 |
| 66 | 39145-15A dup | –8.09 |
| 67 | 39145-16A | –8.18 |
| 68 | 39145-17A | –8.03 |
| 69 | 39145-18A | –8.24 |
| 70 | 39145-19A | –8.14 |
| 71 | 39145-20A | –7.95 |
| 72 | 39145-21A | –8.24 |
| 73 | 39145-22A | –7.84 |
| 74 | 39145-23A | –7.82 |
| 75 | 39145-24A | –7.52 |
| 76 | 39145-25A | –7.61 |
| 77 | 39145-25A dup | –7.58 |
| 78 | 39145-26A | –7.84 |
| 79 | 39145-27A | –7.85 |
| 80 | 39145-28A | –7.70 |
| 81 | 39145-1B | –8.77 |

**Supplementary Table 4D.** Stable carbon (*δ*^13^C_CO3_) isotope data for structural carbonate obtained from bioapatite for sample CMN39148. The suffix A in the sample name indicates subsamples collected along the length of the antler, B = subsamples collected along the mandible.

|  | | ***δ*^13^C_CO3_** |
| --- | --- | --- |
| **Num** | **Sample** | **(VPDB, ‰)** |
| 82 | 39148-1A | –8.68 |
| 83 | 39148-1A dup | –8.53 |
| 84 | 39148-1A rpt | –9.26 |
| 85 | 39148 2A | –8.82 |
| 86 | 39148 2A dup | –8.29 |
| 87 | 39148 2A rpt | –8.23 |
| 88 | 39148 2A rpt dup (in 70^o^c oven ovenight) | –8.16 |
| 89 | 39148 2A rpt dup (in 70^o^c oven overnight) | –8.15 |
| 90 | 39148-3A | –8.84 |
| 91 | 39148-4A | –8.66 |
| 92 | 39148-5A | –8.57 |
| 93 | 39148-6A | –8.45 |
| 94 | 39148-6A dup | –8.44 |
| 95 | 39148-7A | –8.41 |
| 96 | 39148-8A | –8.61 |
| 97 | 39148-9A | –8.21 |
| 98 | 39148-10A | –8.04 |
| 99 | 39148-11A | –8.23 |
| 100 | 39148-11A dup | –8.15 |
| 101 | 39148-12A | –8.38 |
| 102 | 39148-13A | –7.49 |
| 103 | 39148-14A | –7.46 |
| 104 | 39148-15A | –7.31 |
| 105 | 39148-16A | –7.45 |
| 106 | 39148-16A dup | –7.51 |
| 107 | 39148-16A rpt | –8.23 |
| 108 | 39148-17A | –8.37 |
| 109 | 39148-17A dup | –8.32 |
| 110 | 39148-18A | –8.14 |
| 111 | 39148-19A | –8.24 |
| 112 | 39148-20A | –8.07 |
| 113 | 39148-20A dup | –8.12 |
| 114 | 39148-21A | –8.42 |
| 115 | 39148-22A | –8.23 |
| 116 | 39148-22A dup | –8.16 |
| 117 | 39148-23A | –8.24 |
| 118 | 39148-24A | –7.92 |
| 119 | 39148-25A | –7.79 |
| 120 | 39148-1B | –9.27 |

**Supplementary Table 5A.** Stable carbon and nitrogen isotope and elemental data for collagen obtained from sample CMN39079. The suffix A in the sample name indicates subsamples collected along the length of the antler, B = subsamples collected along the mandible.

|  | | ***δ*^13^C_col_** |  | ***δ*^15^N_col_** |  |
| --- | --- | --- | --- | --- | --- |
| **Num** | **Sample** | **(VPDB, ‰)** | **C (wt%)** | **(AIR, ‰)** | **N (wt%)** |
| 121 | 39079-1A | –18.90 | 30.23 | +4.90 | 10.61 |
| 122 | 39079-2A | –18.97 | 43.45 | +5.52 | 15.73 |
| 123 | 39079-3A | –18.91 | 40.22 | +5.53 | 14.43 |
| 124 | 39079-4A | –18.98 | 44.46 | +5.68 | 16.06 |
| 125 | 39079-5A | –19.05 | 39.56 | +5.30 | 14.27 |
| 126 | 39079-6A | –18.93 | 43.64 | +5.67 | 15.79 |
| 127 | 39079-7A | –18.82 | 37.40 | +5.57 | 13.32 |
| 128 | 39079-7A dup | –18.84 | 37.55 | +5.65 | 13.42 |
| 129 | 39079-8A | –18.83 | 35.67 | +5.57 | 12.65 |
| 130 | 39079-9A | –18.83 | 41.90 | +5.88 | 14.99 |
| 131 | 39079-10A | –18.49 | 32.19 | +5.72 | 11.12 |
| 132 | 39079-11A | –18.75 | 36.36 | +5.79 | 12.76 |
| 133 | 39079-12A | –18.90 | 36.06 | +5.71 | 12.70 |
| 134 | 39079-13A | –18.73 | 26.60 | +5.37 | 8.99 |
| 135 | 39079-14A | –18.97 | 36.53 | +5.91 | 12.74 |
| 136 | 39079-15A | –19.01 | 41.15 | +5.97 | 14.55 |
| 137 | 39079-15A dup | –18.91 | 33.45 | +5.74 | 11.52 |
| 138 | 39079-16A | –18.83 | 37.65 | +5.84 | 13.18 |
| 139 | 39079-17A | –19.02 | 44.58 | +6.01 | 15.55 |
| 140 | 39079-18A | –18.94 | 38.93 | +5.86 | 13.26 |
| 141 | 39079-19A | –18.86 | 35.02 | +5.65 | 11.63 |
| 142 | 39079-1B | –18.52 | 22.88 | +3.30 | 7.57 |

**Supplementary Table 5B.** Stable carbon and nitrogen isotope and elemental data for collagen obtained from sample CMN39090. The suffix A in the sample name indicates subsamples collected along the length of the antler, B = subsamples collected along the mandible.

|  | | ***δ*^13^C_col_** |  | ***δ*^15^N_col_** |  |
| --- | --- | --- | --- | --- | --- |
| **Num** | **Sample** | **(VPDB, ‰)** | **C (wt%)** | **(AIR, ‰)** | **N (wt%)** |
| 143 | 39090-1A | –18.40 | 44.16 | +5.28 | 16.05 |
| 144 | 39090-2A | –18.41 | 44.39 | +5.43 | 16.08 |
| 145 | 39090-3A | –18.37 | 40.90 | +5.85 | 14.76 |
| 146 | 39090-4A | –18.37 | 42.15 | +6.05 | 15.35 |
| 147 | 39090-5A | –18.34 | 43.00 | +5.80 | 15.69 |
| 148 | 39090-6A | –18.37 | 42.87 | +5.53 | 15.78 |
| 149 | 39090-7A | –18.41 | 43.19 | +5.60 | 15.84 |
| 150 | 39090-8A | –18.37 | 40.79 | +6.26 | 14.81 |
| 151 | 39090-9A | –18.41 | 43.32 | +6.56 | 15.82 |
| 152 | 39090-10A | –18.38 | 42.83 | +6.31 | 15.59 |
| 153 | 39090-10A dup | –18.37 | 42.55 | +6.31 | 15.50 |
| 154 | 39090-11A | –18.38 | 37.58 | +6.11 | 13.50 |
| 155 | 39090-12A | –18.42 | 37.21 | +6.04 | 13.44 |
| 156 | 39090-13A | –18.50 | 42.80 | +6.06 | 15.46 |
| 157 | 39090-14A | –18.37 | 35.25 | +5.93 | 12.76 |
| 158 | 39090-15A | –18.53 | 42.82 | +6.25 | 15.56 |
| 159 | 39090-16A | –18.53 | 43.60 | +6.18 | 15.82 |
| 160 | 39090-17A | –18.51 | 44.15 | +6.29 | 16.08 |
| 161 | 39090-18A | –18.54 | 42.10 | +6.51 | 15.11 |
| 162 | 39090-18A dup | –18.60 | 42.07 | +6.47 | 15.04 |
| 163 | 39090-19A | –18.53 | 44.16 | +6.43 | 15.97 |
| 164 | 39090-20A | –18.50 | 43.38 | +6.36 | 15.75 |
| 165 | 39090-21A | –18.57 | 42.98 | +6.71 | 15.58 |
| 166 | 39090-22A | –18.57 | 42.57 | +6.62 | 15.46 |
| 167 | 39090-22A dup | –18.60 | 42.79 | +6.61 | 15.48 |
| 168 | 39090-23A | –18.69 | 45.13 | +6.82 | 16.20 |
| 169 | 39090-24A | –18.61 | 44.00 | +6.86 | 15.83 |
| 170 | 39090-25A | –18.60 | 44.32 | +6.92 | 15.83 |
| 171 | 39090-26A | –18.71 | 45.19 | +6.91 | 16.08 |
| 172 | 39090-1B | –18.82 | 43.52 | +4.27 | 15.23 |

**Supplementary Table 5C.** Stable carbon and nitrogen isotope and elemental data for collagen obtained from sample CMN39102. The suffix A in the sample name indicates subsamples collected along the length of the antler, B = subsamples collected along the mandible.

|  | | ***δ*^13^C_col_** |  | ***δ*^15^N_col_** |  |
| --- | --- | --- | --- | --- | --- |
| **Num** | **Sample** | **(VPDB, ‰)** | **C (wt%)** | **(AIR, ‰)** | **N (wt%)** |
| 173 | 39102-1A | –19.12 | 31.89 | +4.19 | 10.99 |
| 174 | 39102-1A dup | –18.97 | 24.33 | +3.79 | 8.20 |
| 175 | 39102-2A | –18.98 | 44.72 | +4.36 | 16.29 |
| 176 | 39102-3A | –19.05 | 44.81 | +4.73 | 16.29 |
| 177 | 39102-4A | –18.97 | 44.15 | +4.83 | 16.10 |
| 178 | 39102-5A | –18.90 | 44.32 | +4.82 | 16.23 |
| 179 | 39102-6A | –18.49 | 20.26 | +4.13 | 6.99 |
| 180 | 39102-7A | –18.82 | 44.33 | +5.20 | 16.22 |
| 181 | 39102-8A | –18.67 | 37.13 | +5.29 | 13.29 |
| 182 | 39102-9A | –18.81 | 44.55 | +5.27 | 16.17 |
| 183 | 39102-10A | –18.88 | 45.24 | +5.19 | 16.43 |
| 184 | 39102-11A | –18.87 | 42.48 | +5.01 | 17.50 |
| 185 | 39102-12A | –18.87 | 39.43 | +5.16 | 16.08 |
| 186 | 39102-13A | –18.87 | 43.94 | +5.23 | 18.15 |
| 187 | 39102-13A dup | –18.94 | 43.82 | +5.03 | 18.04 |
| 188 | 39102-14A | –18.91 | 43.84 | +5.26 | 18.14 |
| 189 | 39102-15A | –18.94 | 44.18 | +5.25 | 18.26 |
| 190 | 39102-16A | –19.00 | 40.48 | +5.21 | 16.49 |
| 191 | 39102-17A | –18.96 | 39.68 | +5.29 | 16.06 |
| 192 | 39102-18A | –19.04 | 44.01 | +5.35 | 18.05 |
| 193 | 39102-19A | –18.84 | 33.48 | +5.06 | 13.30 |
| 194 | 39102-20A | –18.91 | 32.08 | +4.88 | 12.62 |
| 195 | 39102-20A dup | –18.89 | 31.57 | +5.01 | 12.47 |
| 196 | 39102-21A | –18.96 | 42.49 | +5.39 | 17.02 |
| 197 | 39102-22A | –18.66 | 39.73 | +5.29 | 15.74 |
| 198 | 39102-1B | –19.21 | 16.12 | +2.29 | 5.31 |

**Supplementary Table 5D.** Stable carbon and nitrogen isotope and elemental data for collagen obtained from sample CMN39107. The suffix A in the sample name indicates subsamples collected along the length of the antler, B = subsamples collected along the mandible.

|  | | ***δ*^13^C_col_** |  | ***δ*^15^N_col_** |  |
| --- | --- | --- | --- | --- | --- |
| **Num** | **Sample** | **(VPDB, ‰)** | **C (wt%)** | **(AIR, ‰)** | **N (wt%)** |
| 199 | 39107-1A | –19.47 | 35.07 | +2.99 | 12.94 |
| 200 | 39107-2A | –19.29 | 32.33 | +2.79 | 11.89 |
| 201 | 39107-3A | –19.44 | 34.99 | +2.94 | 13.18 |
| 202 | 39107-3A dup | –19.38 | 32.24 | +2.76 | 11.08 |
| 203 | 39107-4A | –19.35 | 43.05 | +3.33 | 16.80 |
| 204 | 39107-5A | –19.43 | 44.01 | +3.48 | 16.99 |
| 205 | 39107-6A | –19.43 | 41.06 | +3.28 | 15.88 |
| 206 | 39107-7A | –19.19 | 33.14 | +2.59 | 11.34 |
| 207 | 39107-8A | –19.43 | 42.61 | +2.94 | 15.14 |
| 208 | 39107-9A | –19.28 | 28.96 | +2.37 | 9.84 |
| 209 | 39107-10A | –19.46 | 44.89 | +2.94 | 15.97 |
| 210 | 39107-11A | –19.38 | 40.98 | +2.81 | 14.47 |
| 211 | 39107-12A | –19.11 | 26.79 | +2.76 | 9.08 |
| 212 | 39107-13A | –19.43 | 45.30 | +2.94 | 16.10 |
| 213 | 39107-14A | –19.43 | 45.15 | +3.06 | 16.02 |
| 214 | 39107-15A | –19.41 | 45.15 | +3.10 | 16.11 |
| 215 | 39107-16A | –19.51 | 44.93 | +3.02 | 16.12 |
| 216 | 39107-17A | –19.44 | 45.02 | +3.21 | 16.16 |
| 217 | 39107-18A | –19.61 | 39.99 | +2.94 | 13.64 |
| 218 | 39107-18A dup | –19.43 | 39.13 | +3.13 | 13.78 |
| 219 | 39107-19A | –19.61 | 39.38 | +2.97 | 13.61 |
| 220 | 39107-20A | –19.52 | 44.54 | +3.13 | 16.07 |
| 221 | 39107-21A | –19.55 | 45.01 | +3.36 | 16.27 |
| 222 | 39107-22A | –19.53 | 44.68 | +3.34 | 16.12 |
| 223 | 39107-23A | –19.54 | 38.05 | +3.14 | 13.42 |
| 224 | 39107-24A | –19.60 | 39.06 | +3.25 | 13.71 |
| 225 | 39107-25A | –19.45 | 31.43 | +3.40 | 10.79 |
| 226 | 39107-26A | –19.52 | 43.98 | +3.86 | 15.77 |
| 227 | 39107-27A | –19.45 | 45.36 | +4.11 | 16.18 |
| 228 | 39107-27A dup | –19.44 | 44.36 | +3.93 | 15.95 |
| 229 | 39107-28A | –19.42 | 43.96 | +4.08 | 15.64 |
| 230 | 39107-29A | –19.25 | 33.06 | +3.57 | 11.16 |
| 231 | 39107-30A | –19.41 | 44.18 | +4.44 | 15.84 |
| 232 | 39107-31A | –19.13 | 40.02 | +4.55 | 14.02 |
| 233 | 39107-1B | –19.28 | 42.75 | +2.67 | 14.72 |

**Supplementary Table 5E.** Stable carbon and nitrogen isotope and elemental data for collagen obtained from sample CMN39108. The suffix A in the sample name indicates subsamples collected along the length of the antler, B = subsamples collected along the mandible.

|  | | ***δ*^13^C_col_** |  | ***δ*^15^N_col_** |  |
| --- | --- | --- | --- | --- | --- |
| **Num** | **Sample** | **(VPDB, ‰)** | **C (wt%)** | **(AIR, ‰)** | **N (wt%)** |
| 234 | 39108-1A | –18.87 | 34.02 | +5.59 | 11.83 |
| 235 | 39108-1A dup | –18.79 | 28.90 | +5.41 | 10.11 |
| 236 | 39108-2A | –18.49 | 20.45 | +5.35 | 6.72 |
| 237 | 39108--2A RPT | –18.90 | 38.21 | +6.11 | 13.54 |
| 238 | 39108-3A | –18.61 | 25.11 | +5.24 | 8.64 |
| 239 | 39108-4A | –18.53 | 25.59 | +5.79 | 8.90 |
| 240 | 39108-5A | –18.75 | 44.38 | +6.65 | 16.01 |
| 241 | 39108-6A | –18.71 | 39.14 | +6.35 | 14.01 |
| 242 | 39108-7A | –18.64 | 30.87 | +5.99 | 10.77 |
| 243 | 39108-8A | –18.77 | 40.64 | +6.28 | 14.60 |
| 244 | 39108-9A | –18.85 | 39.88 | +6.08 | 14.20 |
| 245 | 39108-10A | –18.63 | 25.87 | +5.14 | 8.64 |
| 246 | 39108-11A | –18.50 | 20.59 | +5.72 | 6.84 |
| 247 | 39108--11A RPT | –18.81 | 33.95 | +6.00 | 11.96 |
| 248 | 39108-12A | –18.85 | 33.36 | +5.89 | 11.54 |
| 249 | 39108-12A dup | –18.63 | 26.24 | +5.72 | 8.99 |
| 250 | 39108-13A | –18.50 | 25.57 | +5.69 | 8.73 |
| 251 | 39108-14A | –18.28 | 16.72 | +5.42 | 5.22 |
| 252 | 39108-14A RPT dup | –18.73 | 32.03 | +6.20 | 11.09 |
| 253 | 39108--14A RPT | –18.61 | 27.56 | +6.15 | 9.49 |
| 254 | 39108-15A | –18.66 | 44.66 | +6.52 | 16.11 |
| 255 | 39108-16A | –18.86 | 44.24 | +6.49 | 16.07 |
| 256 | 39108-17A | –18.58 | 31.65 | +6.13 | 10.98 |
| 257 | 39108-18A | –18.88 | 33.68 | +6.20 | 11.85 |
| 258 | 39108-19A | –18.86 | 34.36 | +6.46 | 12.07 |
| 259 | 39108-20A | –18.87 | 39.64 | +6.50 | 14.03 |
| 260 | 39108-21A | –18.79 | 34.63 | +6.39 | 12.26 |
| 261 | 39108-21A dup | –18.52 | 22.98 | +5.58 | 7.84 |
| 262 | 39108--21A RPT | –18.72 | 28.10 | +6.17 | 9.62 |
| 263 | 39108-22A | –18.90 | 41.06 | +6.64 | 14.73 |
| 264 | 39108-23A | –18.58 | 29.66 | +6.09 | 10.16 |
| 265 | 39108-24A | –18.65 | 35.53 | +6.01 | 12.32 |
| 266 | 39108-25A | –18.45 | 39.72 | +6.27 | 13.98 |
| 267 | 39108-1B | –20.47 | 17.99 | +3.62 | 4.93 |
| 268 | 39108-1B dup | –19.01 | 17.66 | +3.70 | 5.48 |
| 269 | 39108--1B RPT | –19.02 | 16.63 | +3.18 | 4.96 |

**Supplementary Table 5F.** Stable carbon and nitrogen isotope and elemental data for collagen obtained from sample CMN39110. The suffix A in the sample name indicates subsamples collected along the length of the antler, B = subsamples collected along the mandible.

|  | | ***δ*^13^C_col_** |  | ***δ*^15^N_col_** |  |
| --- | --- | --- | --- | --- | --- |
| **Num** | **Sample** | **(VPDB, ‰)** | **C (wt%)** | **(AIR, ‰)** | **N (wt%)** |
| 270 | 39110-1A | –19.47 | 35.53 | +3.44 | 12.05 |
| 271 | 39110-2A | –19.23 | 43.75 | +4.73 | 15.12 |
| 272 | 39110-3A | –18.93 | 32.96 | +4.61 | 11.30 |
| 273 | 39110-3A dup | –18.49 | 16.55 | +3.67 | 5.29 |
| 274 | 39110-4A | –18.98 | 35.41 | +4.84 | 12.15 |
| 275 | 39110-5A | –18.87 | 39.13 | +5.32 | 13.55 |
| 276 | 39110-6A | –18.88 | 43.84 | +5.63 | 15.25 |
| 277 | 39110-7A | –19.00 | 33.99 | +4.50 | 11.51 |
| 278 | 39110-8A | –19.19 | 43.79 | +4.80 | 15.19 |
| 279 | 39110-9A | –19.21 | 43.97 | +5.01 | 15.35 |
| 280 | 39110-10A | –19.14 | 44.18 | +4.94 | 15.40 |
| 281 | 39110-11A | –18.91 | 43.74 | +5.89 | 15.16 |
| 282 | 39110-12A | –18.63 | 29.52 | +5.56 | 9.97 |
| 283 | 39110-13A | –18.74 | 43.69 | +6.17 | 15.19 |
| 284 | 39110-14A | –18.83 | 44.22 | +5.95 | 15.95 |
| 285 | 39110-15A | –18.95 | 44.00 | +5.80 | 15.88 |
| 286 | 39110-16A | –19.08 | 33.96 | +5.13 | 12.01 |
| 287 | 39110-17A | –18.78 | 39.61 | +2.53 | 14.01 |
| 288 | 39110-18A | –19.23 | 43.93 | +5.36 | 15.74 |
| 289 | 39110-19A | –19.34 | 41.32 | +5.02 | 14.66 |
| 290 | 39110-20A | –19.12 | 45.15 | +5.31 | 16.30 |
| 291 | 39110-20A dup | –19.19 | 40.35 | +5.28 | 14.40 |
| 292 | 39110-21A | –19.19 | 44.31 | +5.19 | 15.97 |
| 293 | 39110-22A | –18.95 | 35.32 | +5.12 | 12.50 |
| 294 | 39110-23A | –18.87 | 43.94 | +5.93 | 15.85 |
| 295 | 39110-24A | –18.69 | 39.75 | +6.14 | 14.20 |
| 296 | 39110-25A | –18.78 | 44.48 | +6.13 | 15.99 |
| 297 | 39110-26A | –18.73 | 44.35 | +6.29 | 15.92 |
| 298 | 39110-27A | –18.37 | 29.48 | +6.12 | 10.22 |
| 299 | 39110-27A dup | –18.34 | 28.69 | +6.09 | 9.90 |
| 300 | 39110-28A | –18.40 | 31.84 | +6.27 | 11.16 |
| 301 | 39110-29A | –18.33 | 41.16 | +6.77 | 14.54 |
| 302 | 39110-1B | –18.96 | 23.95 | +3.53 | 8.09 |

**Supplementary Table 5G.** Stable carbon and nitrogen isotope and elemental data for collagen obtained from sample CMN39120. The suffix A in the sample name indicates subsamples collected along the length of the antler, B = subsamples collected along the mandible.

|  | | ***δ*^13^C_col_** |  | ***δ*^15^N_col_** |  |
| --- | --- | --- | --- | --- | --- |
| **Num** | **Sample** | **(VPDB, ‰)** | **C (wt%)** | **(AIR, ‰)** | **N (wt%)** |
| 303 | 39120-1A | –18.33 | 17.40 | +4.54 | 5.14 |
| 304 | 39120-2A | –18.71 | 44.26 | +5.65 | 15.26 |
| 305 | 39120-3A | –18.66 | 32.07 | +5.34 | 10.63 |
| 306 | 39120-4A | –18.67 | 38.43 | +5.54 | 12.92 |
| 307 | 39120-5A | –18.78 | 44.75 | +5.55 | 15.37 |
| 308 | 39120-6A | –18.63 | 42.83 | +5.81 | 14.52 |
| 309 | 39120-7A | –18.54 | 35.36 | +5.63 | 11.82 |
| 310 | 39120-7A dup | –18.29 | 23.11 | +5.13 | 7.42 |
| 311 | 39120-8A | –18.59 | 45.16 | +5.90 | 15.32 |
| 312 | 39120-9A | –18.61 | 35.61 | +5.67 | 11.82 |
| 313 | 39120-10A | –18.35 | 23.30 | +5.14 | 7.46 |
| 314 | 39120-11A | –18.75 | 37.46 | +5.84 | 12.41 |
| 315 | 39120-12A | –18.71 | 33.78 | +5.68 | 11.23 |
| 316 | 39120-13A | –18.93 | 37.29 | +5.71 | 12.44 |
| 317 | 39120-14A | –18.88 | 40.12 | +5.89 | 13.39 |
| 318 | 39120-15A | –18.76 | 35.40 | +5.73 | 11.77 |
| 319 | 39120-16A | –18.70 | 43.00 | +5.85 | 14.50 |
| 320 | 39120-17A | –18.92 | 37.04 | +5.54 | 11.82 |
| 321 | 39120-1B | –18.99 | 43.79 | +7.00 | 14.34 |

**Supplementary Table 5H.** Stable carbon and nitrogen isotope and elemental data for collagen obtained from sample CMN39132. The suffix A in the sample name indicates subsamples collected along the length of the antler, B = subsamples collected along the mandible.

|  | | ***δ*^13^C_col_** |  | ***δ*^15^N_col_** |  |
| --- | --- | --- | --- | --- | --- |
| **Num** | **Sample** | **(VPDB, ‰)** | **C (wt%)** | **(AIR, ‰)** | **N (wt%)** |
| 322 | 39132-1A | –19.39 | 28.33 | +4.60 | 10.80 |
| 323 | 39132-2A | –19.29 | 44.00 | +4.65 | 18.19 |
| 324 | 39132-3A | –19.28 | 42.92 | +5.15 | 17.48 |
| 325 | 39132-4A | –19.30 | 44.11 | +5.01 | 18.20 |
| 326 | 39132-5A | –19.25 | 44.35 | +5.08 | 18.28 |
| 327 | 39132-6A | –19.29 | 44.13 | +4.72 | 18.34 |
| 328 | 39132-6A dup | –19.28 | 44.12 | +5.05 | 18.24 |
| 329 | 39132-7A | –19.28 | 44.01 | +4.96 | 18.24 |
| 330 | 39132-8A | –19.03 | 44.04 | +5.54 | 18.00 |
| 331 | 39132-9A | –18.94 | 44.53 | +5.52 | 18.53 |
| 332 | 39132-10A | –19.19 | 44.05 | +5.17 | 17.94 |
| 333 | 39132-11A | –19.01 | 44.33 | +5.31 | 18.41 |
| 334 | 39132-12A | –18.93 | 44.02 | +5.66 | 18.30 |
| 335 | 39132-13A | –18.84 | 44.32 | +5.35 | 18.45 |
| 336 | 39132-14A | –18.88 | 39.78 | +5.29 | 16.47 |
| 337 | 39132-14A dup | –18.81 | 36.02 | +5.37 | 14.72 |
| 338 | 39132-15A | –19.68 | 41.18 | +5.50 | 15.90 |
| 339 | 39132-16A | –19.19 | 42.12 | +5.53 | 16.95 |
| 340 | 39132-17A | –19.07 | 35.03 | +5.25 | 13.93 |
| 341 | 39132-18A | –19.05 | 29.70 | +5.33 | 11.62 |
| 342 | 39132-19A | –19.03 | 35.81 | +5.51 | 14.10 |
| 343 | 39132-20A | –18.91 | 43.04 | +5.75 | 17.27 |
| 344 | 39132-21A | –18.60 | 23.27 | +5.10 | 8.76 |
| 345 | 39132-22A | –18.56 | 23.92 | +4.97 | 8.91 |
| 346 | 39132-23A | –18.75 | 39.29 | +5.47 | 12.64 |
| 347 | 39132-1B | –19.54 | 22.19 | +3.18 | 6.78 |

**Supplementary Table 5I.** Stable carbon and nitrogen isotope and elemental data for collagen obtained from sample CMN39145. The suffix A in the sample name indicates subsamples collected along the length of the antler, B = subsamples collected along the mandible.

|  | | ***δ*^13^C_col_** |  | ***δ*^15^N_col_** |  |
| --- | --- | --- | --- | --- | --- |
| **Num** | **Sample** | **(VPDB, ‰)** | **C (wt%)** | **(AIR, ‰)** | **N (wt%)** |
| 348 | 39145-1A | –18.75 | 39.68 | +3.98 | 14.41 |
| 349 | 39145-2A | –18.78 | 44.44 | +3.86 | 16.32 |
| 350 | 39145-3A | –18.72 | 43.87 | +4.17 | 16.38 |
| 351 | 39145-4A | –18.77 | 44.21 | +4.07 | 16.24 |
| 352 | 39145-5A | –18.65 | 28.18 | +3.58 | 9.80 |
| 353 | 39145-5A dup | –18.45 | 19.48 | +2.49 | 6.39 |
| 354 | 39145-6A | –18.87 | 44.61 | +4.27 | 16.27 |
| 355 | 39145-7A | –18.70 | 28.47 | +3.46 | 9.80 |
| 356 | 39145-8A | –18.71 | 31.19 | +3.72 | 10.81 |
| 357 | 39145-9A | –18.39 | 34.09 | +4.44 | 12.00 |
| 358 | 39145-10A | –18.77 | 40.00 | +4.62 | 14.38 |
| 359 | 39145-11A | –18.35 | 28.68 | +4.46 | 9.80 |
| 360 | 39145-12A | –18.71 | 28.20 | +4.25 | 9.73 |
| 361 | 39145-13A | –18.78 | 40.18 | +4.39 | 14.43 |
| 362 | 39145-14A | –18.66 | 44.55 | +4.66 | 16.04 |
| 363 | 39145-15A | –18.74 | 33.21 | +4.39 | 11.67 |
| 364 | 39145-16A | –18.65 | 42.19 | +4.67 | 15.34 |
| 365 | 39145-16A dup | –18.81 | 40.32 | +4.62 | 14.70 |
| 366 | 39145-17A | –18.70 | 29.75 | +4.33 | 10.28 |
| 367 | 39145-18A | –18.85 | 40.54 | +4.84 | 14.44 |
| 368 | 39145-19A | –18.82 | 33.33 | +4.79 | 11.83 |
| 369 | 39145-20A | –18.78 | 28.51 | +4.38 | 9.62 |
| 370 | 39145-21A | –18.90 | 37.09 | +5.20 | 13.33 |
| 371 | 39145-22A | –18.55 | 26.95 | +4.90 | 9.40 |
| 372 | 39145-23A | –18.76 | 32.95 | +5.27 | 11.85 |
| 373 | 39145-24A dup | –18.76 | 38.18 | +5.42 | 13.72 |
| 374 | 39145-25A | –18.68 | 41.87 | +5.48 | 13.92 |
| 375 | 39145-26A | –18.59 | 32.88 | +5.32 | 11.52 |
| 376 | 39145-27A | –18.89 | 42.72 | +5.85 | 15.47 |
| 377 | 39145-28A | –18.84 | 37.28 | +5.79 | 13.07 |
| 378 | 39145-1B | –18.64 | 18.99 | +3.22 | 6.29 |
| 379 | 39145-1B RPT | –18.78 | 19.74 | +3.79 | 6.43 |
| 380 | 39145-5A RPT | –18.62 | 21.66 | +3.47 | 7.32 |

**Supplementary Table 5J.** Stable carbon and nitrogen isotope and elemental data for collagen obtained from sample CMN39148. The suffix A in the sample name indicates subsamples collected along the length of the antler, B = subsamples collected along the mandible.

|  | | ***δ*^13^C_col_** |  | ***δ*^15^N_col_** |  |
| --- | --- | --- | --- | --- | --- |
| **Num** | **Sample** | **(VPDB, ‰)** | **C (wt%)** | **(AIR, ‰)** | **N (wt%)** |
| 381 | 39148-1A | –19.35 | 44.43 | +4.04 | 16.49 |
| 382 | 39148-2A | –19.24 | 43.72 | +3.99 | 16.60 |
| 383 | 39148-3A | –19.24 | 42.94 | +3.91 | 16.36 |
| 384 | 39148-4A | –19.29 | 43.48 | +3.91 | 16.57 |
| 385 | 39148-5A | –19.22 | 43.91 | +4.31 | 16.59 |
| 386 | 39148-6A | –19.22 | 43.09 | +4.40 | 16.32 |
| 387 | 39148-7A | –19.07 | 44.08 | +3.98 | 16.17 |
| 388 | 39148-8A | –19.13 | 44.19 | +4.25 | 16.30 |
| 389 | 39148-8A dup | –19.51 | 44.78 | +4.27 | 15.94 |
| 390 | 39148-9A | –19.20 | 44.22 | +4.37 | 16.18 |
| 391 | 39148-10A | –19.24 | 45.33 | +4.40 | 16.30 |
| 392 | 39148-11A | –19.14 | 45.28 | +4.58 | 16.45 |
| 393 | 39148-12A | –19.24 | 44.17 | +4.68 | 15.99 |
| 394 | 39148-13A | –19.22 | 44.36 | +4.39 | 16.08 |
| 395 | 39148-14A | –19.16 | 44.84 | +4.46 | 16.26 |
| 396 | 39148-15A | –19.13 | 44.74 | +4.32 | 16.37 |
| 397 | 39148-16A | –19.11 | 44.70 | +4.48 | 16.40 |
| 398 | 39148-17A | –19.02 | 44.79 | +4.62 | 16.56 |
| 399 | 39148-18A | –19.25 | 44.80 | +4.64 | 15.96 |
| 400 | 39148-19A | –19.11 | 44.79 | +4.55 | 16.45 |
| 401 | 39148-20A | –19.04 | 44.88 | +4.53 | 16.48 |
| 402 | 39148-20A dup | –19.01 | 45.02 | +4.52 | 16.57 |
| 403 | 39148-21A | –19.06 | 44.38 | +4.60 | 16.23 |
| 404 | 39148-22A | –18.98 | 45.05 | +4.76 | 16.47 |
| 405 | 39148-23A | –19.02 | 44.56 | +4.68 | 16.02 |
| 406 | 39148-24A | –18.80 | 44.54 | +4.73 | 16.10 |
| 407 | 39148-25A | –18.89 | 44.88 | +4.96 | 15.96 |
| 408 | 39148-1B | –19.07 | 44.85 | +3.23 | 15.75 |

**Supplementary Table 5K.** Stable carbon and nitrogen isotope and elemental data for collagen obtained from sample CMN39149. The suffix A in the sample name indicates subsamples collected along the length of the antler, B = subsamples collected along the mandible.

|  | | ***δ*^13^C_col_** |  | ***δ*^15^N_col_** |  |
| --- | --- | --- | --- | --- | --- |
| **Num** | **Sample** | **(VPDB, ‰)** | **C (wt%)** | **(AIR, ‰)** | **N (wt%)** |
| 409 | 39149-1A | –19.05 | 43.29 | +4.60 | 15.48 |
| 410 | 39149-2A | –18.94 | 42.86 | +4.61 | 15.75 |
| 411 | 39149-3A | –18.95 | 45.37 | +4.58 | 16.75 |
| 412 | 39149-4A | –18.82 | 38.17 | +4.47 | 13.82 |
| 413 | 39149-5A | –18.36 | 17.12 | +2.26 | 5.01 |
| 414 | 39149-5A dup | –18.42 | 19.99 | +3.49 | 6.61 |
| 415 | 39149-6A | –18.98 | 41.94 | +4.91 | 15.11 |
| 416 | 39149-7A | –18.83 | 37.71 | +4.66 | 13.68 |
| 417 | 39149-8A | –18.87 | 44.98 | +4.80 | 16.59 |
| 418 | 39149-9A | –18.66 | 28.54 | +3.95 | 9.94 |
| 419 | 39149-9A dup | –18.62 | 27.18 | +3.90 | 9.35 |
| 420 | 39149-10A | –18.62 | 33.75 | +4.68 | 12.03 |
| 421 | 39149-11A | –18.87 | 45.75 | +5.01 | 16.71 |
| 422 | 39149-12A | –18.60 | 29.08 | +4.27 | 9.95 |
| 423 | 39149-13A | –18.64 | 41.41 | +5.04 | 15.10 |
| 424 | 39149-14A | –18.35 | 23.05 | +3.63 | 7.61 |
| 425 | 39149-14A dup | –18.60 | 32.83 | +4.69 | 11.53 |
| 426 | 39149-15A | –18.65 | 35.08 | +4.79 | 12.56 |
| 427 | 39149-16A | –19.05 | 45.23 | +5.10 | 16.11 |
| 428 | 39149-17A | –18.64 | 45.08 | +5.19 | 16.62 |
| 429 | 39149-18A | –18.69 | 45.24 | +5.02 | 16.69 |
| 430 | 39149-19A | –18.73 | 45.26 | +5.37 | 16.58 |
| 431 | 39149-20A | –18.86 | 35.95 | +4.67 | 12.75 |
| 432 | 39149-21A | –18.87 | 43.88 | +5.06 | 15.99 |
| 433 | 39149-22A | –18.85 | 32.97 | +4.58 | 11.47 |
| 434 | 39149-23A | –18.89 | 36.02 | +4.50 | 12.58 |
| 435 | 39149-24A | –18.73 | 38.73 | +4.86 | 13.86 |
| 436 | 39149-24A dup | –18.72 | 37.02 | +4.86 | 13.26 |
| 437 | 39149-25A | –18.37 | 35.40 | +4.48 | 12.52 |
| 438 | 39149-26A | –18.32 | 34.10 | +4.25 | 11.92 |
| 439 | 39149-27A | –18.50 | 32.13 | +4.05 | 10.60 |
| 440 | 39149-1B | –19.52 | 43.71 | +4.31 | 13.96 |

**Supplementary Table 5L.** Stable carbon and nitrogen isotope and elemental data for collagen obtained from sample CMN39151. The suffix A in the sample name indicates subsamples collected along the length of the antler, B = subsamples collected along the mandible.

|  | | ***δ*^13^C_col_** |  | ***δ*^15^N_col_** |  |
| --- | --- | --- | --- | --- | --- |
| **Num** | **Sample** | **(VPDB, ‰)** | **C (wt%)** | **(AIR, ‰)** | **N (wt%)** |
| 441 | 39151-1A | –19.05 | 45.32 | +4.40 | 16.76 |
| 442 | 39151-2A | –19.14 | 45.55 | +4.57 | 16.96 |
| 443 | 39151-3A | –19.10 | 45.59 | +4.65 | 16.93 |
| 444 | 39151-4A | –19.07 | 45.08 | +4.70 | 16.78 |
| 445 | 39151-5A | –19.06 | 44.91 | +4.77 | 16.78 |
| 446 | 39151-6A | –19.02 | 45.94 | +4.89 | 17.18 |
| 447 | 39151-7A | –19.06 | 45.26 | +5.21 | 16.92 |
| 448 | 39151-7A dup | –19.04 | 45.03 | +4.79 | 16.89 |
| 449 | 39151-8A | –19.08 | 45.15 | +5.13 | 16.81 |
| 450 | 39151-9A | –19.01 | 45.36 | +5.32 | 16.98 |
| 451 | 39151-10A | –19.04 | 45.07 | +5.11 | 16.67 |
| 452 | 39151-11A | –19.10 | 45.59 | +5.09 | 16.97 |
| 453 | 39151-11A dup | –19.17 | 45.26 | +5.07 | 16.92 |
| 454 | 39151-12A | –19.00 | 45.51 | +5.58 | 16.86 |
| 455 | 39151-13A | –19.04 | 45.26 | +5.35 | 16.91 |
| 456 | 39151-14A | –19.15 | 44.41 | +5.13 | 16.59 |
| 457 | 39151-15A | –19.08 | 44.97 | +5.40 | 16.86 |
| 458 | 39151-16A | –19.01 | 45.06 | +5.51 | 16.95 |
| 459 | 39151-17A | –19.05 | 44.77 | +5.67 | 16.93 |
| 460 | 39151-18A | –18.98 | 44.59 | +5.63 | 16.09 |
| 461 | 39151-19A | –19.00 | 44.11 | +5.64 | 15.84 |
| 462 | 39151-20A | –18.96 | 44.63 | +5.72 | 16.06 |
| 463 | 39151-20A dup | –19.02 | 44.59 | +5.79 | 16.01 |
| 464 | 39151-21A | –19.09 | 44.70 | +5.60 | 16.02 |
| 465 | 39151-22A | –19.10 | 44.43 | +5.72 | 16.00 |
| 466 | 39151-23A | –19.03 | 44.71 | +5.80 | 16.09 |
| 467 | 39151-24A | –18.96 | 44.94 | +6.02 | 16.06 |
| 468 | 39151-25A | –18.93 | 44.23 | +6.06 | 15.75 |
| 469 | 39151-26A | –18.76 | 46.00 | +6.18 | 16.30 |
| 470 | 39151-1B | –18.79 | 45.14 | +4.99 | 15.60 |

**Supplementary Table 6.** Linear regressions for *δ*^13^C_col_ and *δ*^15^N_col_ variation along antler length for each individual caribou.

| **Specimen** | **Isotope** |  | **Coefficients** | **Standard Error** | **T Stat** | **P-value** | **Lower 95%** | **Upper 95%** |
| --- | --- | --- | --- | --- | --- | --- | --- | --- |
| CMN39079 | *δ*^13^C_col_ | Intercept | –18.890 | 0.0540 | –349.903 | < 0.0001 | –19.003 | –18.777 |
|  |  | X Variable | 0.000 | 0.0017 | 0.242 | 0.811 | –0.003 | 0.004 |
|  | *δ*^15^N_col_ | Intercept | 5.383 | 0.0843 | 63.847 | < 0.0001 | 5.207 | 5.560 |
|  |  | X Variable | 0.010 | 0.0027 | 3.793 | 0.001 | 0.005 | 0.016 |
| CMN39090 | *δ*^13^C_col_ | Intercept | –18.317 | 0.0186 | –986.810 | < 0.0001 | –18.356 | –18.279 |
|  |  | X Variable | –0.004 | 0.0004 | –10.172 | < 0.0001 | –0.005 | –0.003 |
|  | *δ*^15^N_col_ | Intercept | 5.577 | 0.0811 | 68.765 | < 0.0001 | 5.4111 | 5.743 |
|  |  | X Variable | 0.017 | 0.0018 | 9.464 | < 0.0001 | 0.014 | 0.021 |
| CMN39102 | *δ*^13^C_col_ | Intercept | –18.916 | 0.0505 | –374.338 | < 0.0001 | -19.021 | –18.812 |
|  |  | X Variable | 0.001 | 0.0014 | 0.550 | 0.588 | –0.002 | 0.004 |
|  | *δ*^15^N_col_ | Intercept | 4.510 | 0.1187 | 37.995 | < 0.0001 | 4.264 | 4.755 |
|  |  | X Variable | 0.015 | 0.0032 | 4.568 | 0.0001 | 0.008 | 0.021 |
| CMN39107 | *δ*^13^C_col_ | Intercept | –19.389 | 0.0410 | –478.281 | < 0.0001 | –19.472 | –19.307 |
|  |  | X Variable | -0.001 | 0.0010 | –0.931 | 0.359 | –0.002 | 0.001 |
|  | *δ*^15^N_col_ | Intercept | 2.645 | 0.1170 | 22.613 | < 0.0001 | 2.407 | 2.884 |
|  |  | X Variable | 0.014 | 0.0022 | 6.043 | < 0.0001 | 0.009 | 0.018 |
| CMN39108 | *δ*^13^C_col_ | Intercept | –18.705 | 0.0531 | –352.112 | < 0.0001 | –18.812 | –18.597 |
|  |  | X Variable | 0.000 | 0.0013 | 0.341 | 0.735 | -0.0022 | 0.003 |
|  | *δ*^15^N_col_ | Intercept | 5.711 | 0.1259 | 45.367 | < 0.0001 | 5.454 | 5.967 |
|  |  | X Variable | 0.008 | 0.0030 | 2.760 | 0.009 | 0.002 | 0.015 |
| CMN39110 | *δ*^13^C_col_ | Intercept | –19.133 | 0.0917 | –208.712 | < 0.0001 | –19.320 | –18.945 |
|  |  | X Variable | 0.005 | 0.0018 | 2.952 | 0.006 | 0.002 | 0.009 |
|  | *δ*^15^N_col_ | Intercept | 4.411 | 0.2497 | 17.662 | < 0.0001 | 3.901 | 4.921 |
|  |  | X Variable | 0.021 | 0.0050 | 4.149 | 0.0003 | 0.011 | 0.031 |
| CMN39120 | *δ*^13^C_col_ | Intercept | –18.503 | 0.0750 | –246.859 | < 0.0001 | –18.661 | –18.344 |
|  |  | X Variable | –0.007 | 0.0027 | –2.382 | 0.0300 | –0.012 | –0.001 |
|  | *δ*^15^N_col_ | Intercept | 5.292 | 0.1390 | 38.071 | < 0.0001 | 4.998 | 5.587 |
|  |  | X Variable | 0.012 | 0.0050 | 2.291 | 0.0359 | 0.000 | 0.022 |
| CMN39132 | *δ*^13^C_col_ | Intercept | –19.356 | 0.0777 | –248.992 | < 0.0001 | –19.517 | –19.195 |
|  |  | X Variable | 0.009 | 0.0021 | 4.217 | 0.0003 | 0.004 | 0.013 |
|  | *δ*^15^N_col_ | Intercept | 4.947 | 0.1000 | 49.452 | < 0.0001 | 4.740 | 5.154 |
|  |  | X Variable | 0.009 | 0.0026 | 3.346 | 0.0028 | 0.003 | 0.014 |
| CMN39145 | *δ*^13^C_col_ | Intercept | –18.664 | 0.0474 | –393.958 | < 0.0001 | –18.761 | –18.570 |
|  |  | X Variable | –0.001 | 0.0010 | –1.188 | 0.245 | –0.003 | 0.001 |
|  | *δ*^15^N_col_ | Intercept | 3.518 | 0.1367 | 25.742 | < 0.0001 | 3.238 | 3.798 |
|  |  | X Variable | 0.025 | 0.0029 | 8.587 | < 0.0001 | 0.0193 | 0.031 |
| CMN39148 | *δ*^13^C_col_ | Intercept | –19.327 | 0.0360 | –534.338 | < 0.0001 | –19.402 | –19.253 |
|  |  | X Variable | 0.005 | 0.0010 | 5.918 | < 0.0001 | 0.003 | 0.007 |
|  | *δ*^15^N_col_ | Intercept | 4.025 | 0.0520 | 77.761 | < 0.0001 | 3.918 | 4.132 |
|  |  | X Variable | 0.011 | 0.0010 | 8.874 | < 0.0001 | 0.008 | 0.013 |
| CMN39149 | *δ*^13^C_col_ | Intercept | –18.808 | 0.0710 | –264.483 | < 0.0001 | –18.954 | –18.663 |
|  |  | X Variable | 0.002 | 0.0020 | 1.577 | 0.126 | –0.001 | 0.006 |
|  | *δ*^15^N_col_ | Intercept | 4.270 | 0.2105 | 20.284 | < 0.0001 | 3.840 | 4.701 |
|  |  | X Variable | 0.007 | 0.0047 | 1.425 | 0.1648 | –0.0029 | 0.016 |
| CMN39151 | *δ*^13^C_col_ | Intercept | –19.106 | 0.0250 | –778.441 | < 0.0001 | –19.157 | –19.056 |
|  |  | X Variable | 0.002 | 0.0010 | 3.237 | 0.003 | 0.001 | 0.003 |
|  | *δ*^15^N_col_ | Intercept | 4.569 | 0.0500 | 90.858 | < 0.0001 | 4.466 | 4.672 |
|  |  | X Variable | 0.020 | 0.0010 | 17.517 | < 0.0001 | 0.018 | 0.023 |

**Supplementary Table 7.** Bayesian stable isotope mixing model mean probabilities with 95% credible intervals.

|  | **Mean - 95% Credible Interval** | | | | |  |
| --- | --- | --- | --- | --- | --- | --- |
| **Model** | **Fungi** | **Horsetail** | **Lichen** | **Liverwort** | **Woody** |  |
| Uninformative Priors |  |  |  |  |  |  |
| No Effects | 0.263 – 0.318 | 0.202 – 0.249 | 0.231 – 0.277 | 0.017 – 0.028 | 0.288 – 0.339 |  |
| - Lower Antler Segment | 0.190 – 0.260 | 0.169 – 0.237 | 0.284 – 0.355 | 0.037 – 0.071 | 0.319 – 0.392 |  |
| - Middle Antler Segment | 0.218 – 0.281 | 0.180 – 0.242 | 0.267 – 0.327 | 0.030 – 0.057 | 0.305 – 0.368 |  |
| - Upper Antler Segment | 0.199 – 0.274 | 0.169 – 0.244 | 0.288 – 0.370 | 0.051 – 0.101 | 0.293 – 0.364 |  |
| Antler as a Continuous Covariate | 0.216 – 0.294 | 0.078 – 0.238 | 0.178 – 0.260 | 0.326 – 0.537 | 0.202 – 0.315 |  |
| - Lower Antler Segment | 0.067 – 0.245 | 0.133 – 0.303 | 0.421 – 0.574 | 0.046 – 0.156 | 0.333 – 0.449 |  |
| - Middle Antler Segment | 0.000 – 0.000 | 0.342 – 0.481 | 0.231 – 0.442 | 0.051 – 0.163 | 0.375 – 0.520 |  |
| - Upper Antler Segment | 0.208 – 0.295 | 0.154 – 0.242 | 0.291 – 0.384 | 0.048 – 0.103 | 0.299 – 0.381 |  |
| Informative Priors |  |  |  |  |  |  |
| No Effects Antler | 0.068 – 0.238 | 0.211 – 0.365 | 0.268 – 0.441 | 0.177 – 0.571 | 0.276 – 0.397 |  |
| - Lower Antler Segment | | 0.000 – 0.000 | 0.187 – 0.317 | 0.322 – 0.418 | 0.199 – 0.574 | 0.292 – 0.404 |
| - Middle Antler Segment | | 0.074 – 0.260 | 0.259 – 0.348 | 0.310 – 0.393 | 0.030 – 0.062 | 0.327 – 0.391 |
| - Upper Antler Segment | | 0.277 – 0.324 | 0.089 – 0.221 | 0.291 – 0.389 | 0.109 – 0.362 | 0.284 – 0.380 |
| Antler as a Continuous Covariate | 0.116 – 0.389 | 0.250 – 0.354 | 0.256 – 0.501 | 0.014 – 0.034 | 0.363 – 0.441 |  |
| - Lower Antler Segment | | 0.000 – 0.000 | 0.229 – 0.324 | 0.405 – 0.541 | 0.044 – 0.127 | 0.322 – 0.418 |
| - Middle Antler Segment | | 0.077 – 0.284 | 0.261 – 0.348 | 0.299 – 0.393 | 0.038 – 0.084 | 0.326 – 0.399 |
| - Upper Antler Segment | | 0.254 – 0.366 | 0.114 – 0.295 | 0.081 – 0.307 | 0.266 – 0.522 | 0.285 – 0.460 |

**Supplementary Table 8.** Bayesian stable isotope mixing models compared using Leave-One-Out Information Criterion (LOOIC), the difference between each model and the model with the lowest LOOIC, and calculated Akaike weights (estimated probability that the model will make the best prediction on new data).

| **Models** | |  |  |  |  |  |
| --- | --- | --- | --- | --- | --- | --- |
| **Priors** | **Antler Length** | **LOOIC** | **SE** | **dLOOIC** | **SE dLOOIC** | **Akaike Weight** |
| **All Antler Tissue** | |  |  |  |  |  |
| Uninformative | Continuous Covariate | 19.7 | 20.0 | 0.0 | NA | 0.634 |
| Informative | No Effect | 20.8 | 21.2 | 1.1 | 5.8 | 0.366 |
| Uninformative | No Effect | 53.4 | 18.6 | 33.7 | 5.2 | 0.000 |
| Informative | Continuous Covariate | 95.3 | 17.1 | 75.6 | 3.0 | 0.000 |
| **Lower Antler Segment** | |  |  |  |  |  |
| Uninformative | No Effect | –12.1 | 4.1 | 0.0 | NA | 0.643 |
| Informative | No Effect | –10.7 | 4.1 | 1.4 | 0.5 | 0.319 |
| Uninformative | Continuous Covariate | –6.4 | 3.8 | 5.7 | 1.0 | 0.037 |
| Informative | Continuous Covariate | 35.8 | 2.9 | 47.9 | 1.5 | 0.000 |
| **Middle Antler Segment** | |  |  |  |  |  |
| Uninformative | Continuous Covariate | –15.9 | 9.7 | 0.0 | NA | 0.574 |
| Uninformative | No Effect | –15.3 | 10.3 | 0.6 | 2.6 | 0.426 |
| Informative | Continuous Covariate | 57.2 | 7.2 | 73.1 | 2.5 | 0.000 |
| Informative | No Effect | 67.2 | 7.3 | 83.1 | 3.1 | 0.000 |
| **Upper Antler Segment** | |  |  |  |  |  |
| Uninformative | No Effect | –4.8 | 7.8 | 0.0 | NA | 1 |
| Uninformative | Continuous Covariate | 11.1 | 7.2 | 15.9 | 0.9 | 0 |
| Informative | No Effect | 39.2 | 6.1 | 44.0 | 2.0 | 0 |
| Informative | Continuous Covariate | 78.7 | 4.8 | 83.5 | 3.3 | 0 |


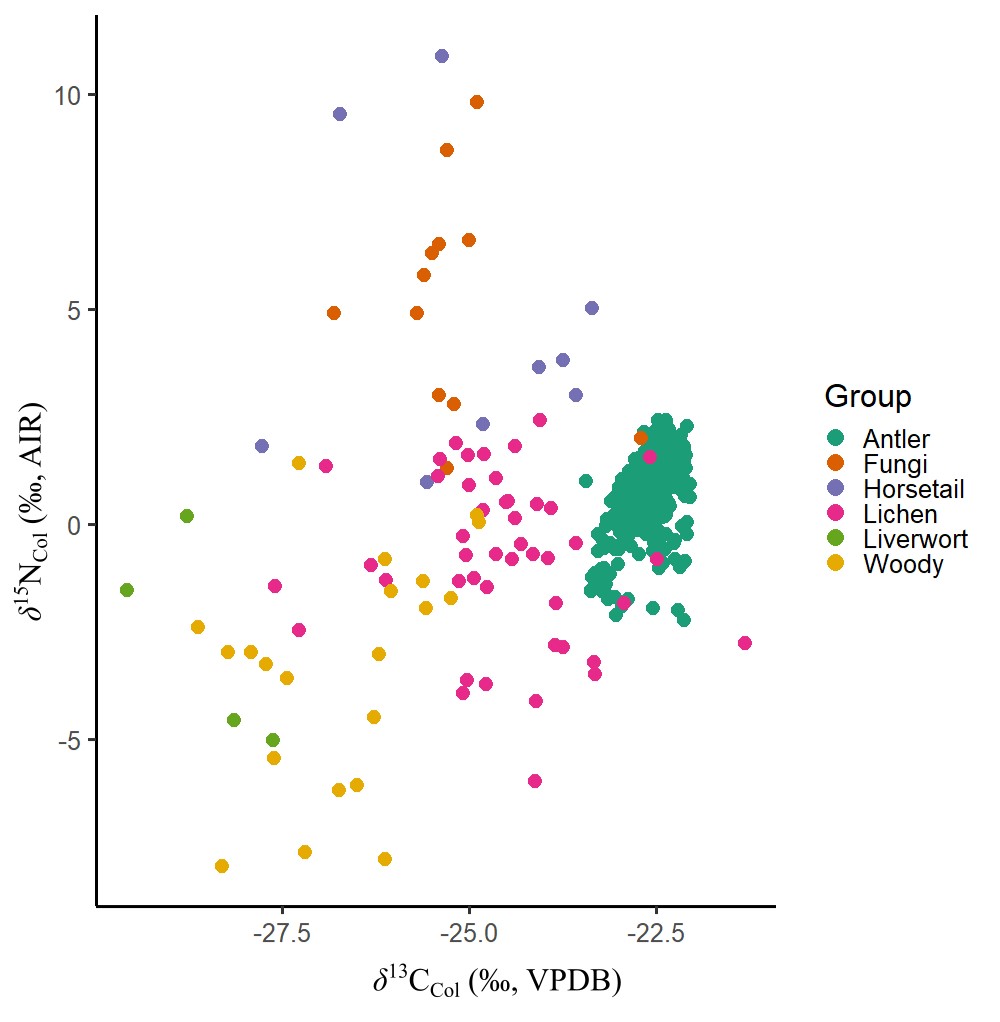


**Supplementary Figure 1.** *δ*^13^C_col_ and *δ*^15^N_col_ antler values for all male Qamanirjuaq caribou adjusted for trophic enrichment factor compared to *δ*^13^C and *δ*^15^N isotope mix for food sources.


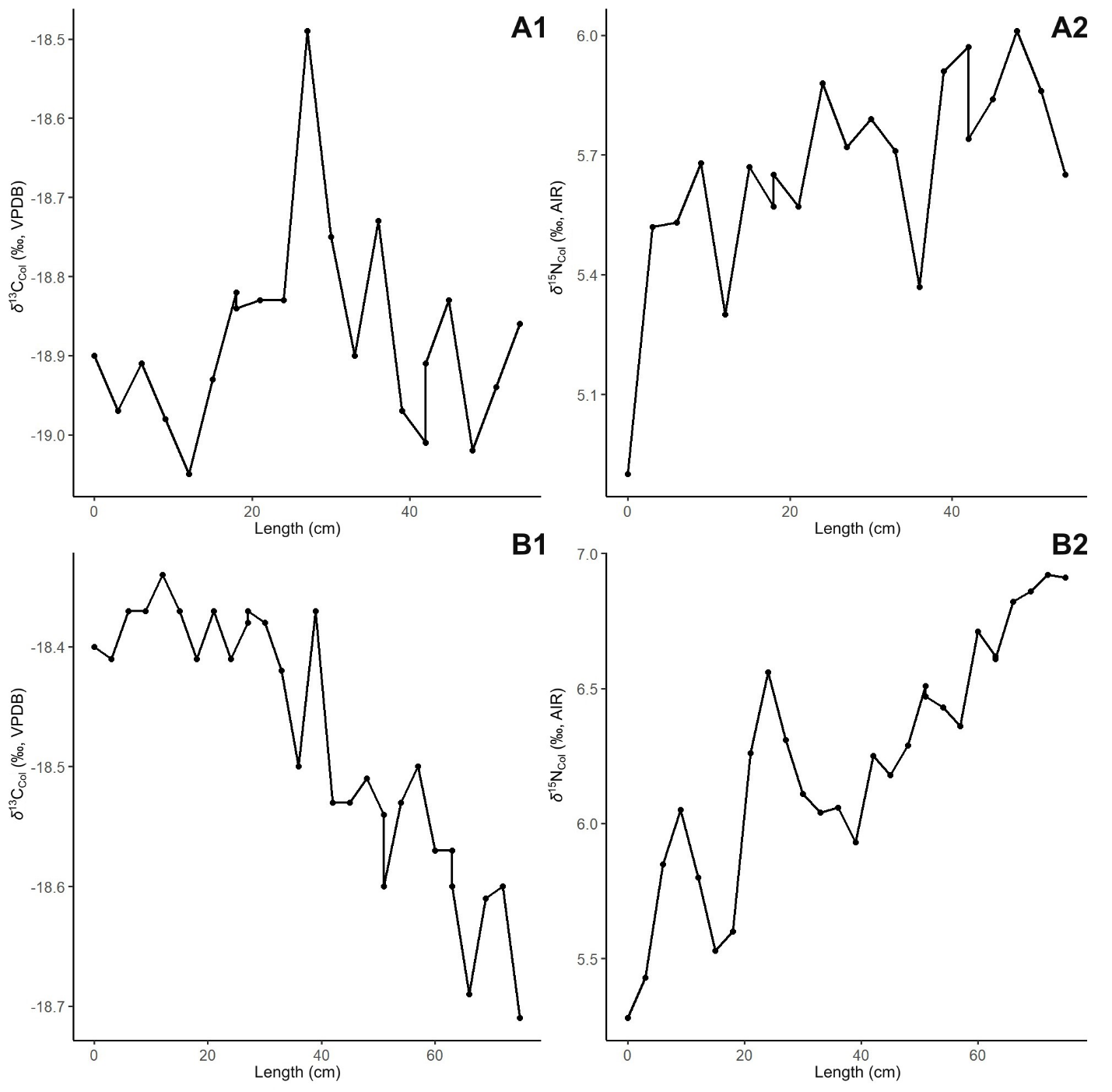
**Supplementary Figure 2A & B: (A1)** *δ*^13^C_col_ variation along antler length for CMN39079; **(A2)** *δ*^15^N_col_ variation along antler length for CMN39079; **(B1)** *δ*^13^C_col_ variation along antler length for CMN39090; **(B2)** *δ*^15^N_col_ variation along antler length for CMN39090.


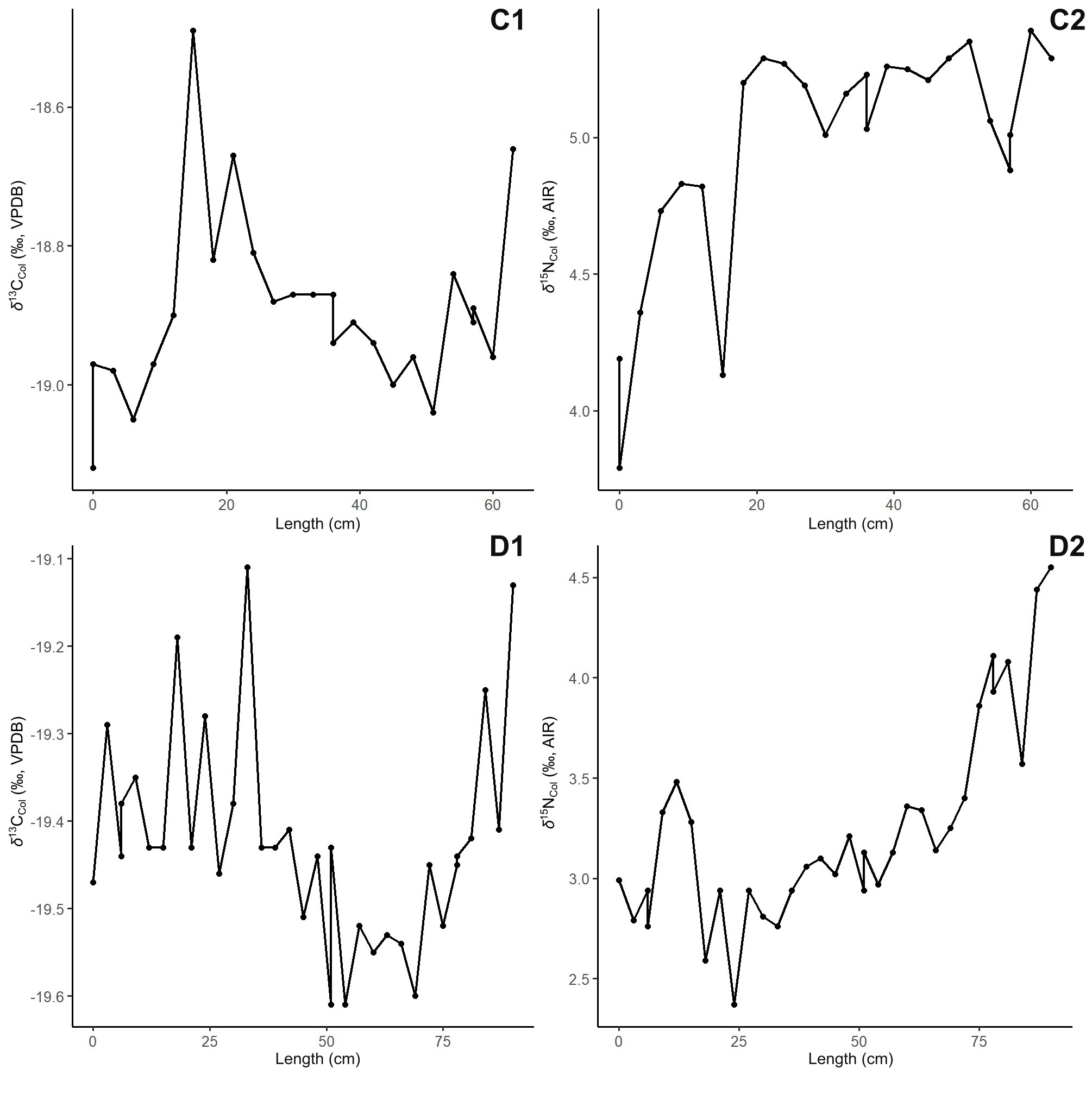
**Supplementary Figure 2C & D: (C1)** *δ*^13^C_col_ variation along antler length for CMN39102; **(C2)** *δ*^15^N_col_ variation along antler length for CMN39102; **(D1)** *δ*^13^C_col_ variation along antler length for CMN39107; **(D2)** *δ*^15^N_col_ variation along antler length for CMN39107.
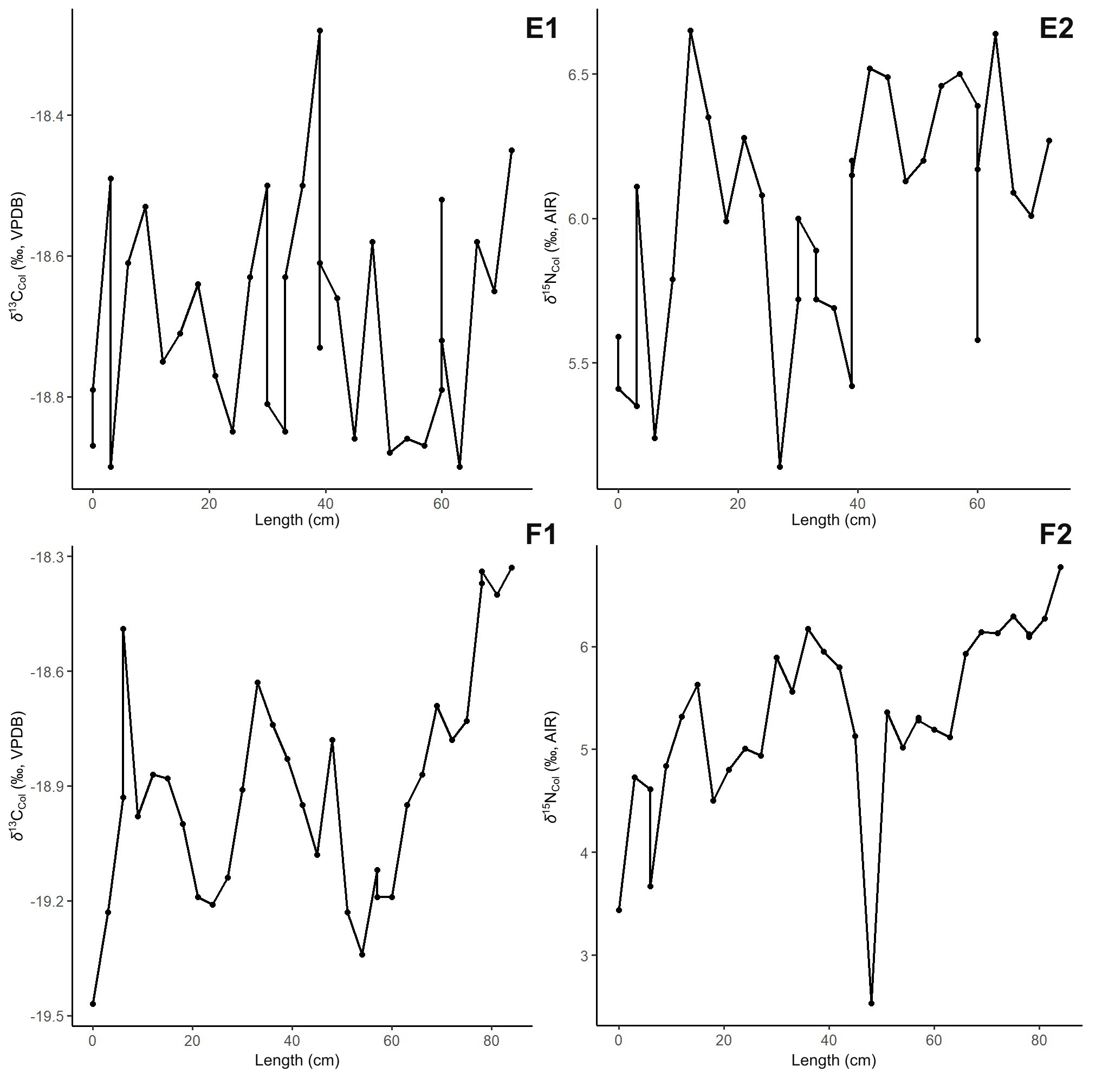
 **Supplementary Figure 2E & F: (E1)** *δ*^13^C_col_ variation along antler length for CMN39108; **(E2)** *δ*^15^N_col_ variation along antler length for CMN39108; **(F1)** *δ*^13^C_col_ variation along antler length for CMN39110; **(F2)** *δ*^15^N_col_ variation along antler length for CMN39110.
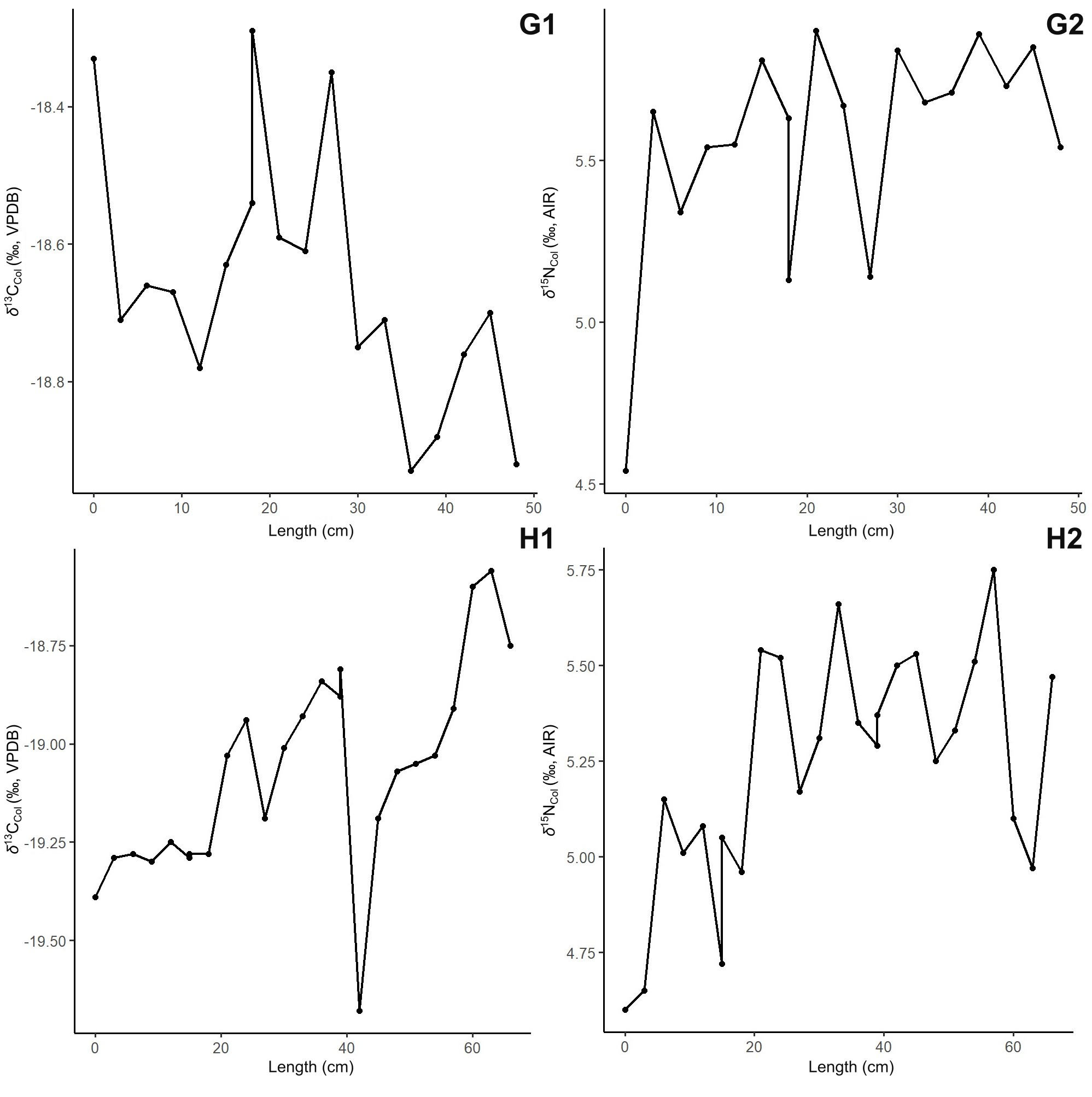
 **Supplementary Figure 2G & H: (G1)** *δ*^13^C_col_ variation along antler length for CMN39120; **(G2)** *δ*^15^N_col_ variation along antler length for CMN39120; **(H1)** *δ*^13^C_col_ variation along antler length for CMN39132; **(H2)** *δ*^15^N_col_ variation along antler length for CMN39132.


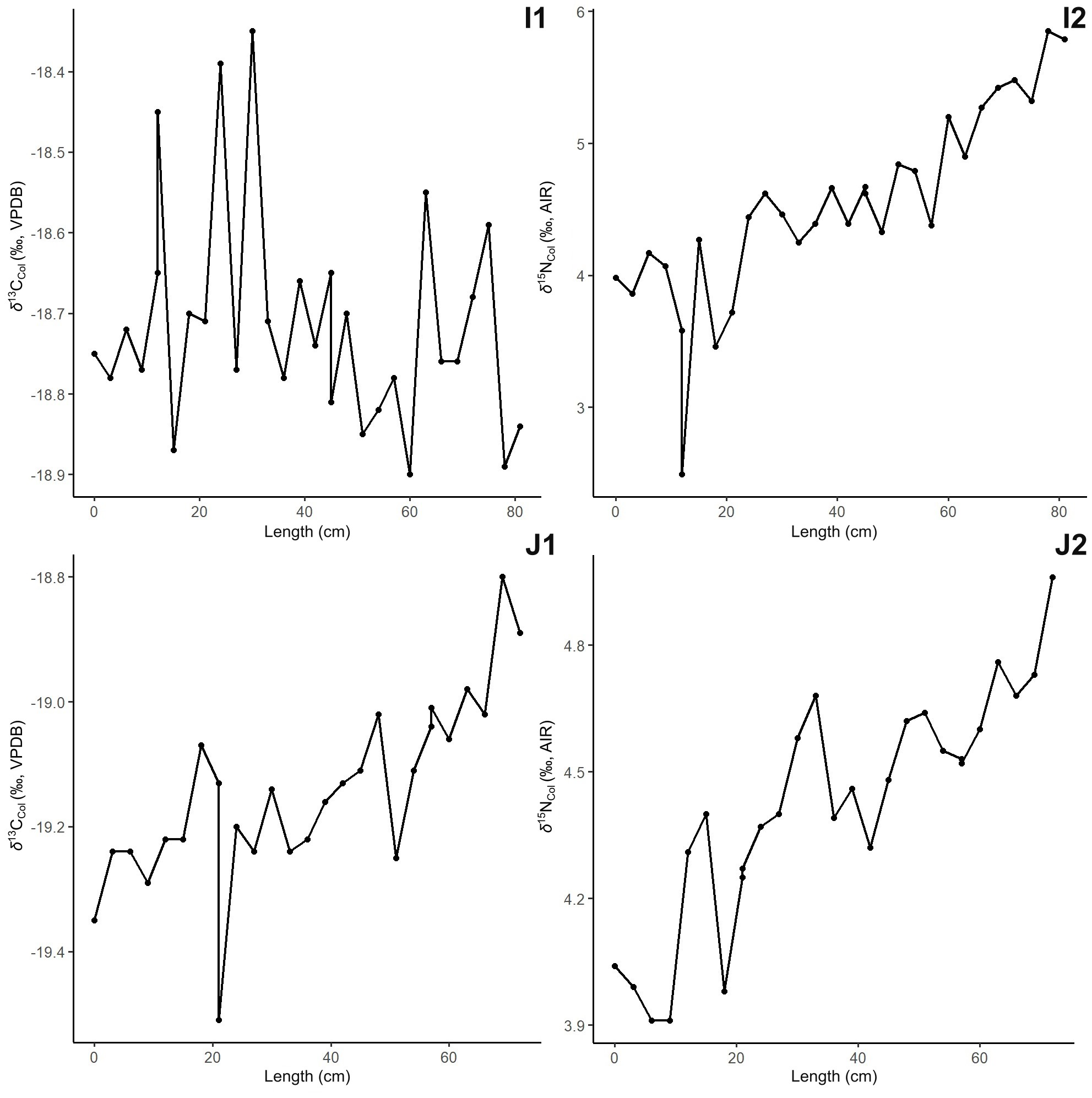
 **Supplementary Figure 2I & J: (I1)** *δ*^13^C_col_ variation along antler length for CMN39145; **(I2)** *δ*^15^N_col_ variation along antler length for CMN39145; **(J1)** *δ*^13^C_col_ variation along antler length for CMN39148; **(J2)** *δ*^15^N_col_ variation along antler length for CMN39148.

**Supplementary Figure 2K & L: (K1)** *δ*^13^C_col_ variation along antler length for CMN39149; **(K2)** *δ*^15^N_col_ variation along antler length for CMN39149; **(L1)** *δ*^13^C_col_ variation along antler length for CMN39151; **(L2)** *δ*^15^N_col_ variation along antler length for CMN39151
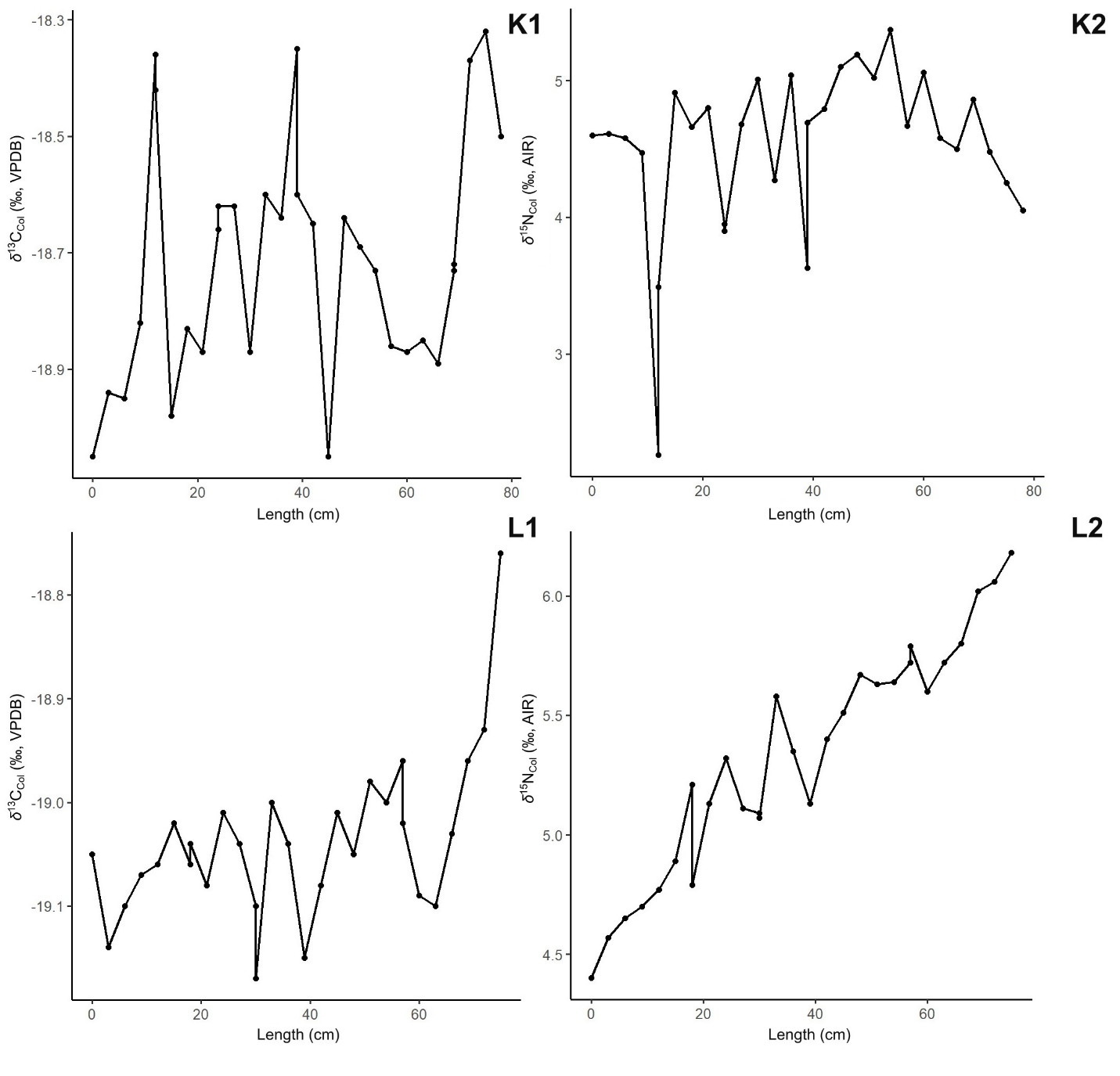
.


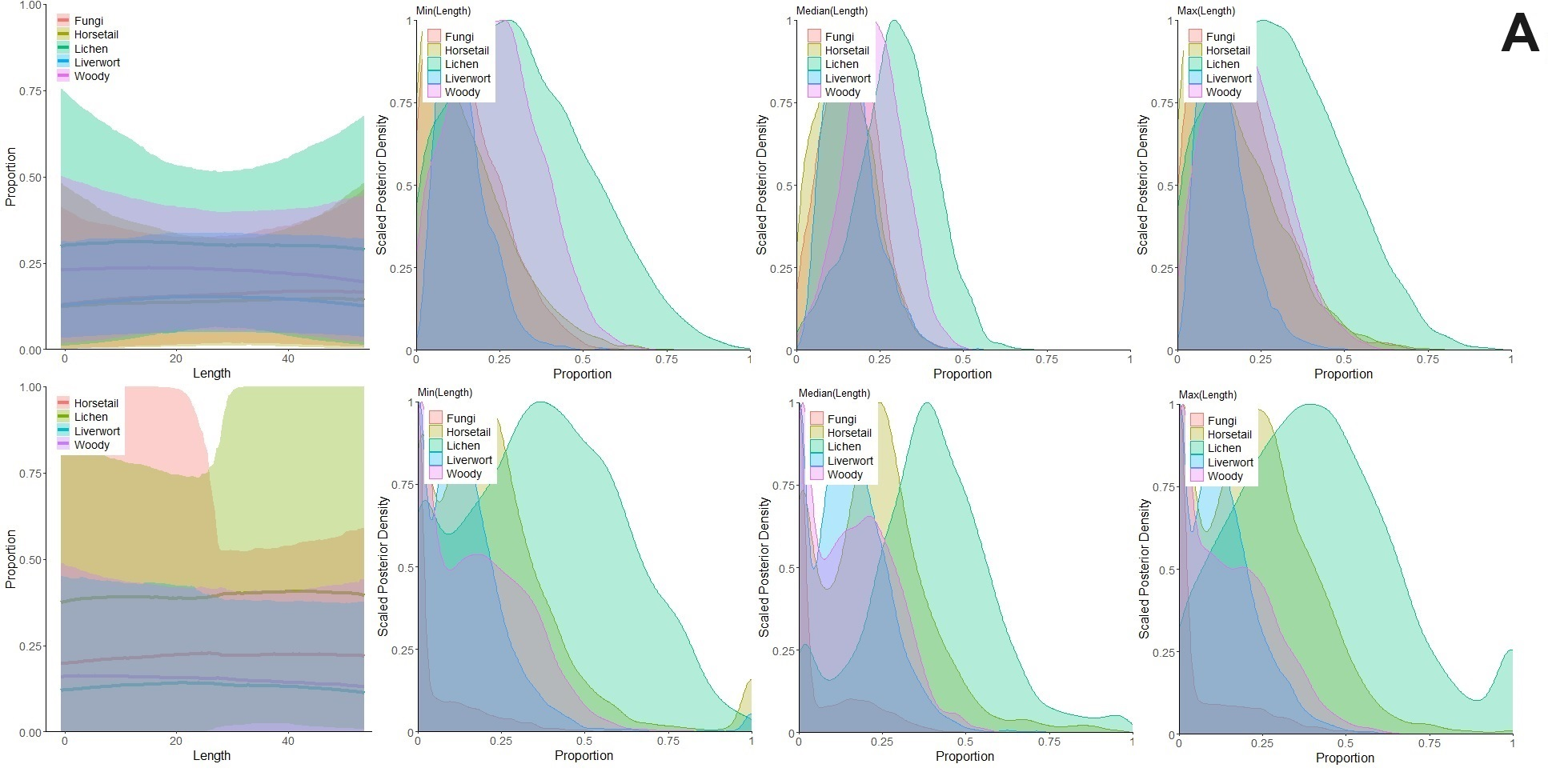


**Supplementary Figure 3A.** *Top row (left to right)*: Estimated diet mixture proportions throughout the antler’s length for sample CMN39079 using uninformative priors, followed by the scaled posterior density versus proportion for each food source measured at three different intervals throughout the antler (the minimum length of the antler (i.e., the base), the middle length of the antler (i.e., the middle), and the maximum length of the antler (i.e., the tip). *Bottom row (left to right)*: Estimated diet mixture proportions throughout the antler’s length for sample CMN39079 using informative priors, followed by the scaled posterior density versus proportion for each food source measured at three different intervals throughout the antler (the minimum length of the antler (i.e., the base), the middle length of the antler (i.e., the middle), and the maximum length of the antler (i.e., the tip).


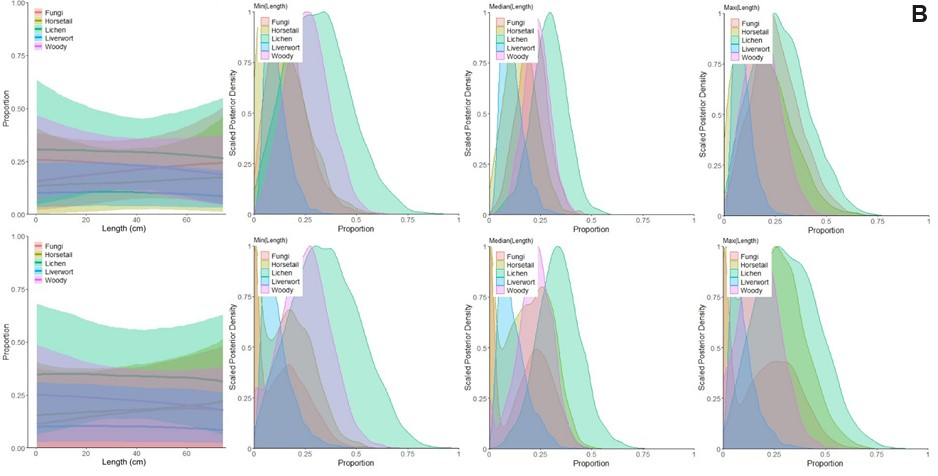
 **Supplementary Figure 3B.** *Top row (left to right)*: Estimated diet mixture proportions throughout the antler’s length for sample CMN39090 using uninformative priors, followed by the scaled posterior density versus proportion for each food source measured at three different intervals throughout the antler (the minimum length of the antler (i.e., the base), the middle length of the antler (i.e., the middle), and the maximum length of the antler (i.e., the tip). *Bottom row (left to right)*: Estimated diet mixture proportions throughout the antler’s length for sample CMN39090 using informative priors, followed by the scaled posterior density versus proportion for each food source measured at three different intervals throughout the antler (the minimum length of the antler (i.e., the base), the middle length of the antler (i.e., the middle), and the maximum length of the antler (i.e., the tip).


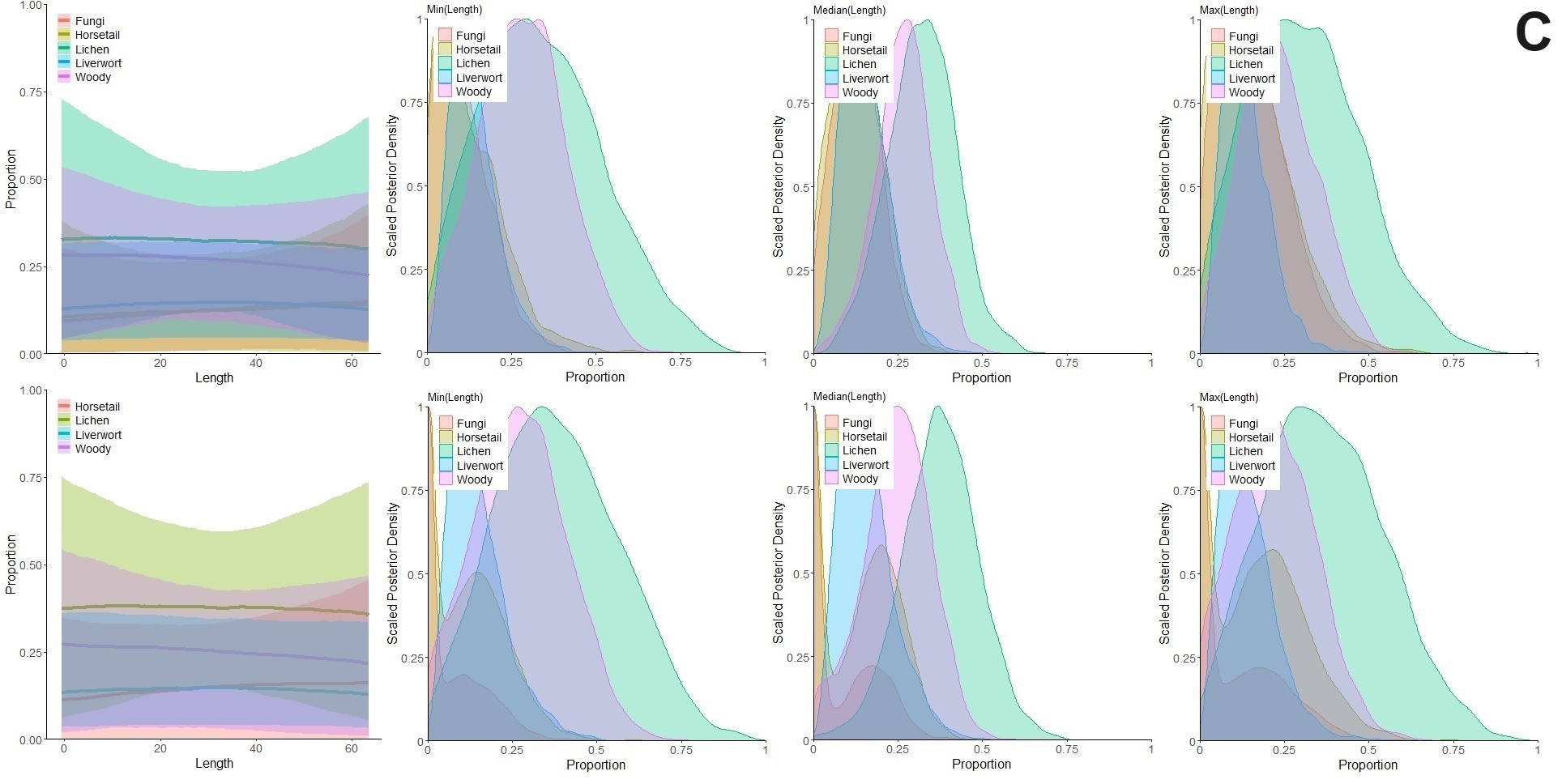


**Supplementary Figure 3C.** *Top row (left to right)*: Estimated diet mixture proportions throughout the antler’s length for sample CMN39102 using uninformative priors, followed by the scaled posterior density versus proportion for each food source measured at three different intervals throughout the antler (the minimum length of the antler (i.e., the base), the middle length of the antler (i.e., the middle), and the maximum length of the antler (i.e., the tip). *Bottom row (left to right)*: Estimated diet mixture proportions throughout the antler’s length for sample CMN39102 using informative priors, followed by the scaled posterior density versus proportion for each food source measured at three different intervals throughout the antler (the minimum length of the antler (i.e., the base), the middle length of the antler (i.e., the middle), and the maximum length of the antler (i.e., the tip).


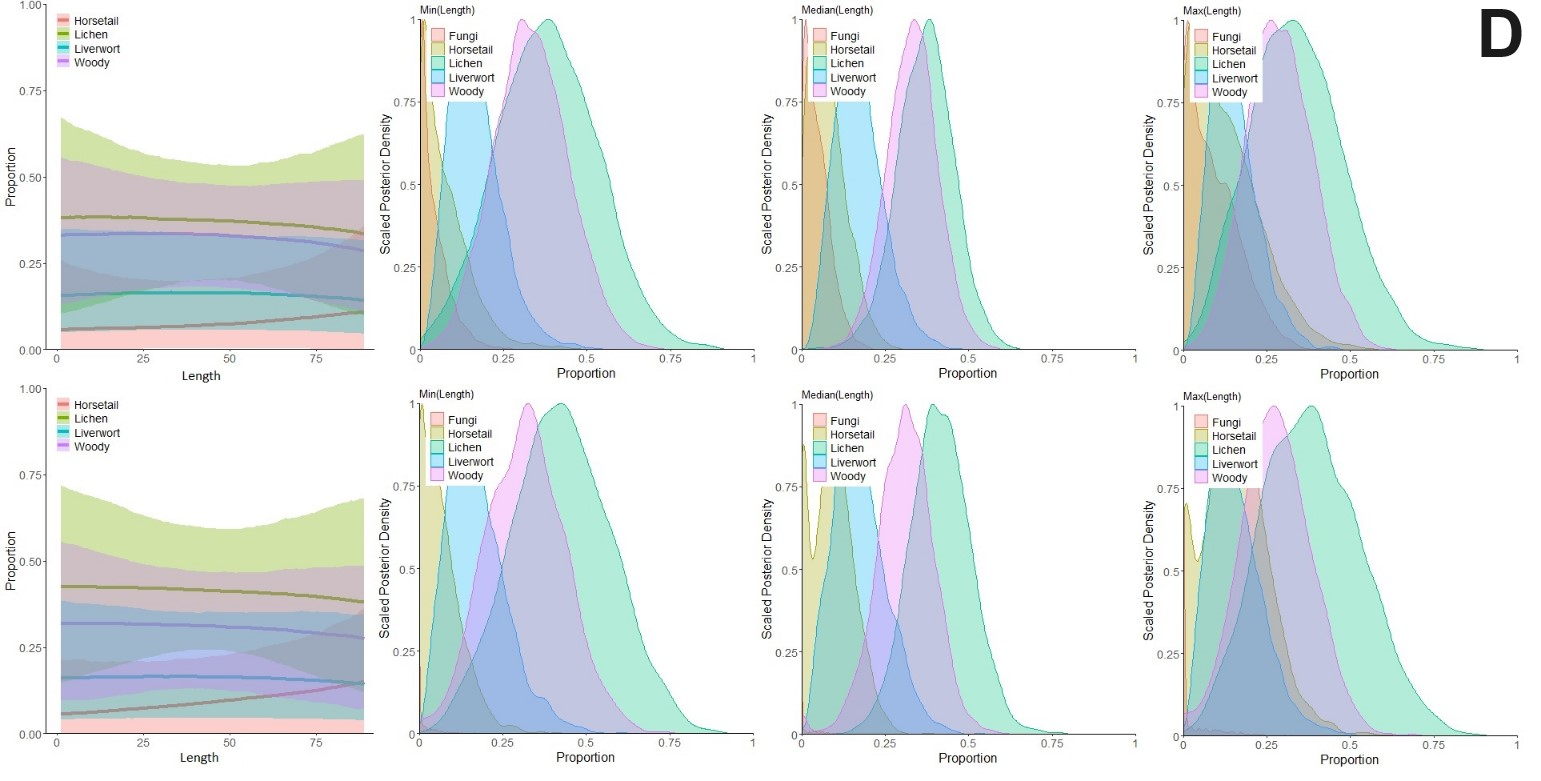
 **Supplementary Figure 3D.** *Top row (left to right)*: Estimated diet mixture proportions throughout the antler’s length for sample CMN39107 using uninformative priors, followed by the scaled posterior density versus proportion for each food source measured at three different intervals throughout the antler (the minimum length of the antler (i.e., the base), the middle length of the antler (i.e., the middle), and the maximum length of the antler (i.e., the tip). *Bottom row (left to right)*: Estimated diet mixture proportions throughout the antler’s length for sample CMN39107 using informative priors, followed by the scaled posterior density versus proportion for each food source measured at three different intervals throughout the antler (the minimum length of the antler (i.e., the base), the middle length of the antler (i.e., the middle), and the maximum length of the antler (i.e., the tip).


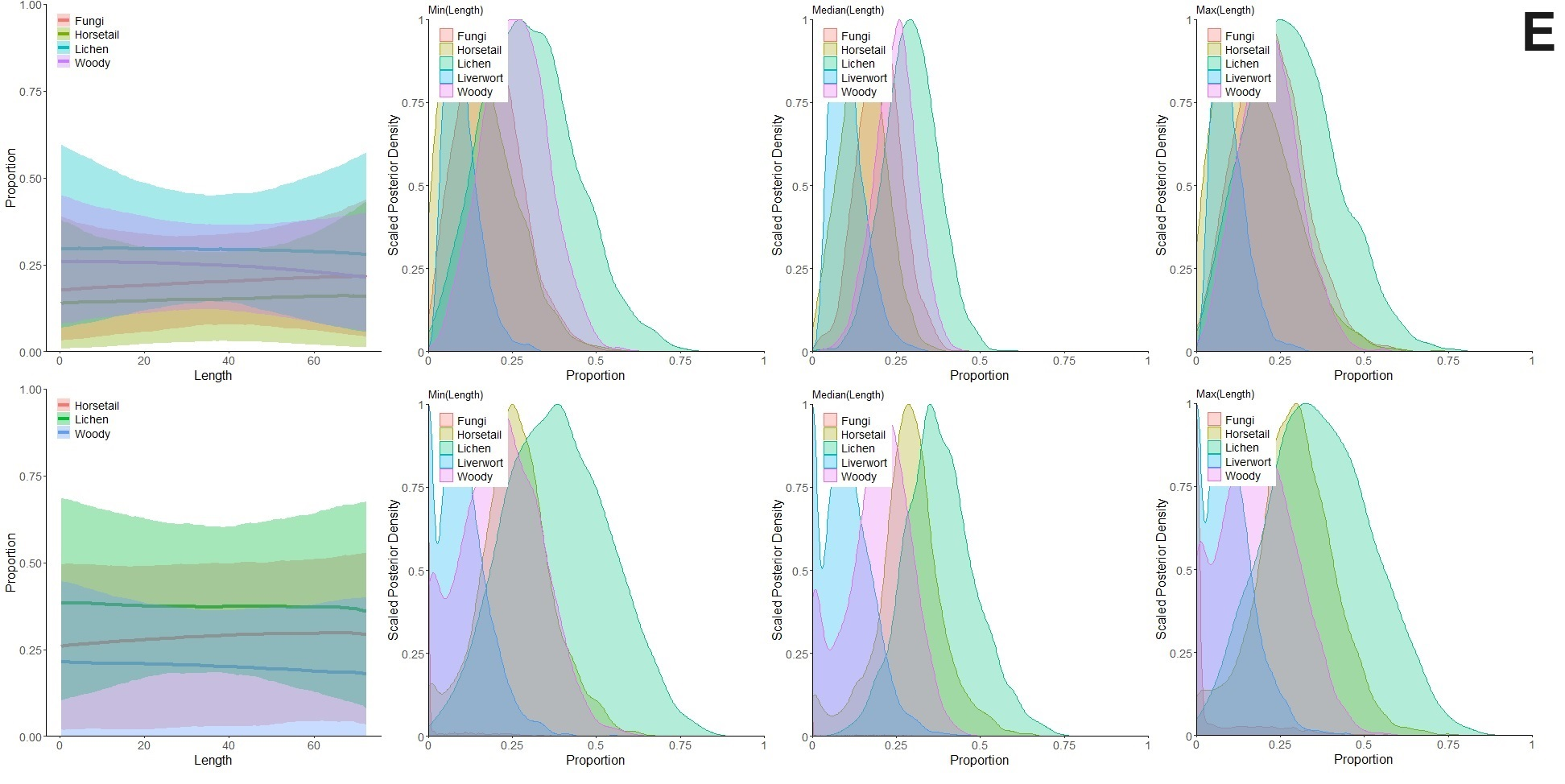


**Supplementary Figure 3E.** *Top row (left to right)*: Estimated diet mixture proportions throughout the antler’s length for sample CMN39108 using uninformative priors, followed by the scaled posterior density versus proportion for each food source measured at three different intervals throughout the antler (the minimum length of the antler (i.e., the base), the middle length of the antler (i.e., the middle), and the maximum length of the antler (i.e., the tip). *Bottom row (left to right)*: Estimated diet mixture proportions throughout the antler’s length for sample CMN39108 using informative priors, followed by the scaled posterior density versus proportion for each food source measured at three different intervals throughout the antler (the minimum length of the antler (i.e., the base), the middle length of the antler (i.e., the middle), and the maximum length of the antler (i.e., the tip).


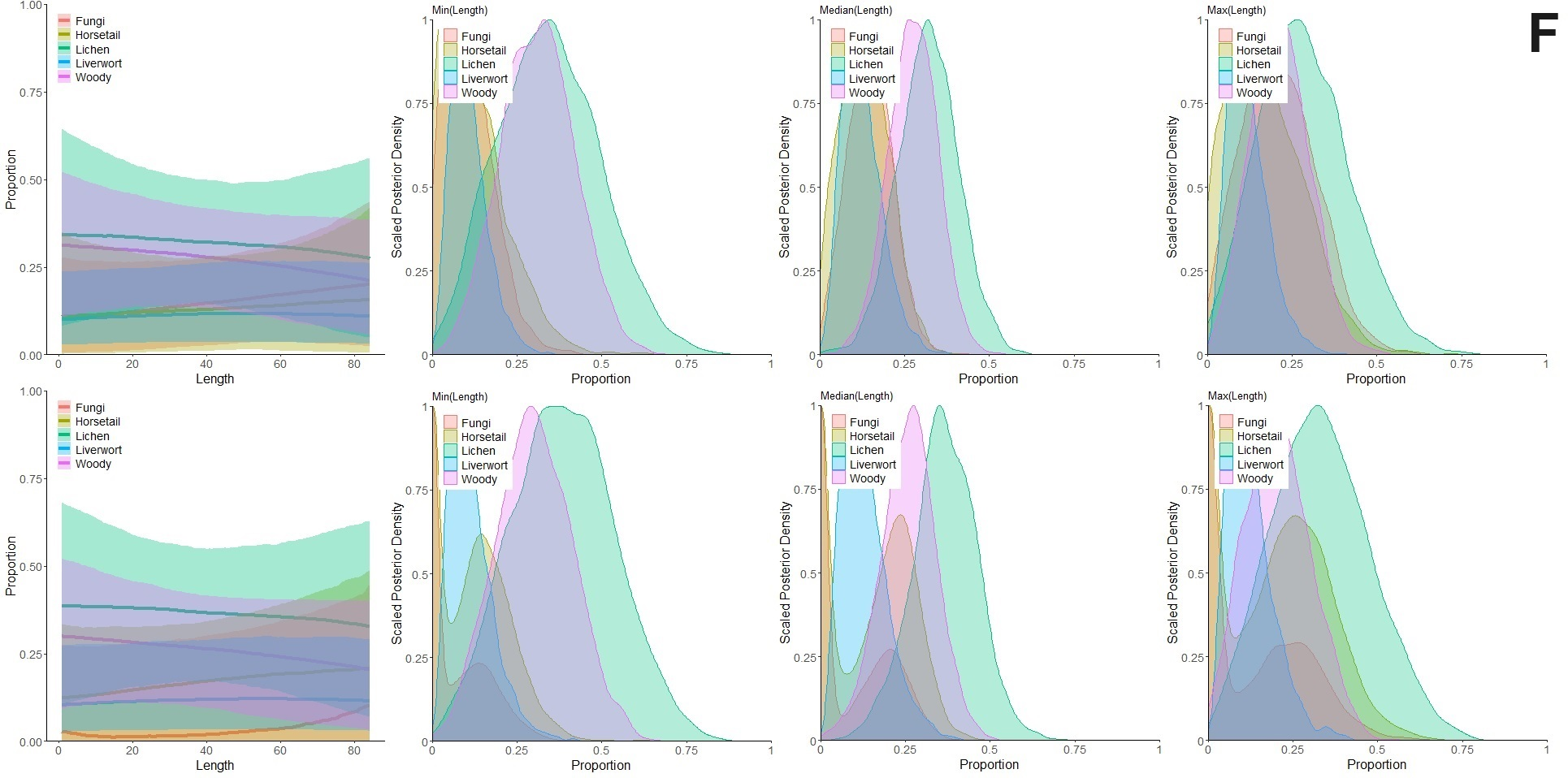


**Supplementary Figure 3F.** *Top row (left to right)*: Estimated diet mixture proportions throughout the antler’s length for sample CMN39110 using uninformative priors followed by the scaled posterior density versus proportion for each food source measured at three different intervals throughout the antler (the minimum length of the antler (i.e., the base), the middle length of the antler (i.e., the middle), and the maximum length of the antler (i.e., the tip). *Bottom row (left to right)*: Estimated diet mixture proportions throughout the antler’s length for sample CMN39110 using informative priors, followed by the scaled posterior density versus proportion for each food source measured at three different intervals throughout the antler (the minimum length of the antler (i.e., the base), the middle length of the antler (i.e., the middle), and the maximum length of the antler (i.e., the tip).


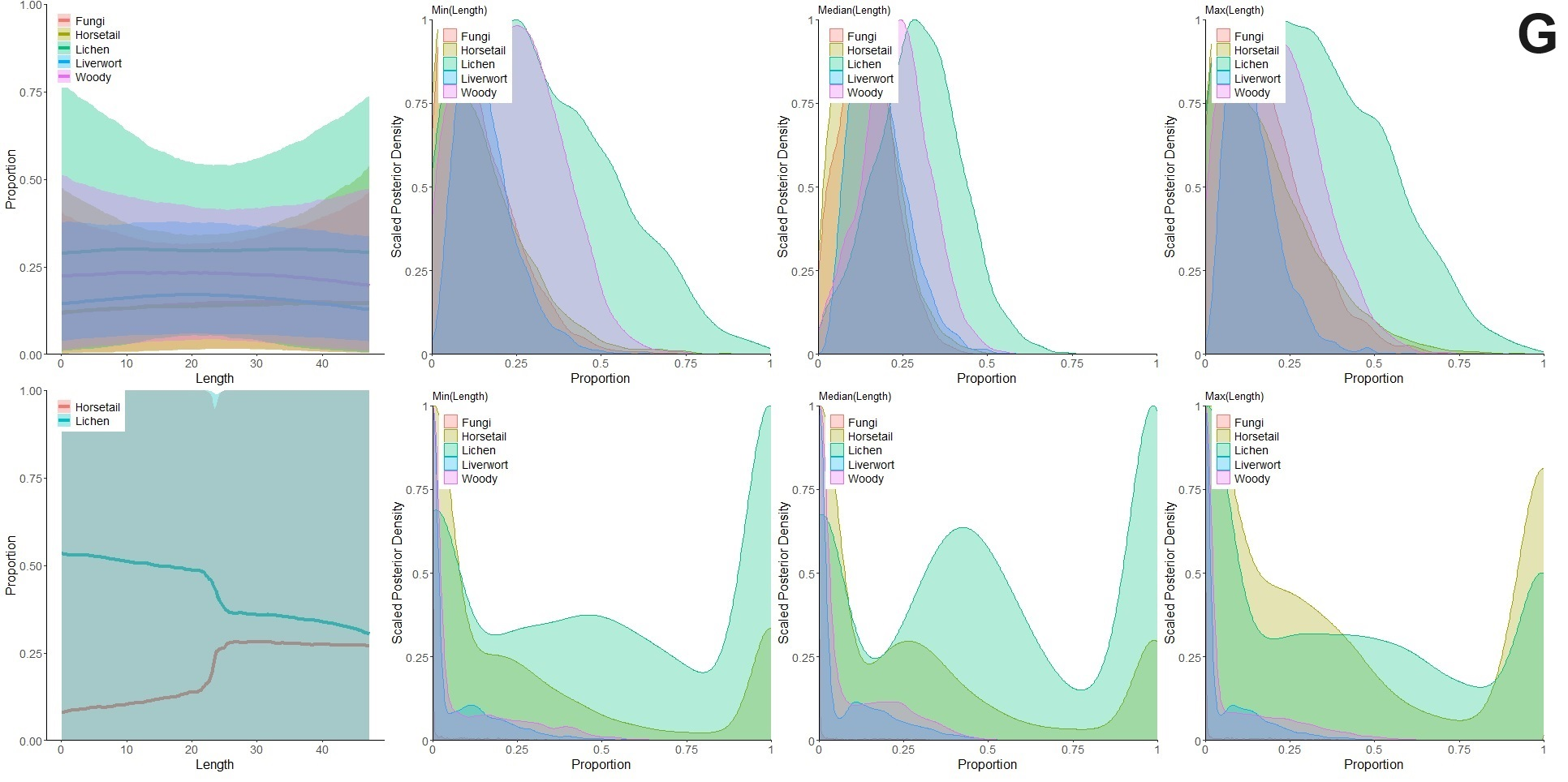


**Supplementary Figure 3G.** *Top row (left to right)*: Estimated diet mixture proportions throughout the antler’s length for sample CMN39120 using uninformative priors, followed by the scaled posterior density versus proportion for each food source measured at three different intervals throughout the antler (the minimum length of the antler (i.e., the base), the middle length of the antler (i.e., the middle), and the maximum length of the antler (i.e., the tip). *Bottom row (left to right)*: Estimated diet mixture proportions throughout the antler’s length for sample CMN39120 using informative priors, followed by the scaled posterior density versus proportion for each food source measured at three different intervals throughout the antler (the minimum length of the antler (i.e., the base), the middle length of the antler (i.e., the middle), and the maximum length of the antler (i.e., the tip).


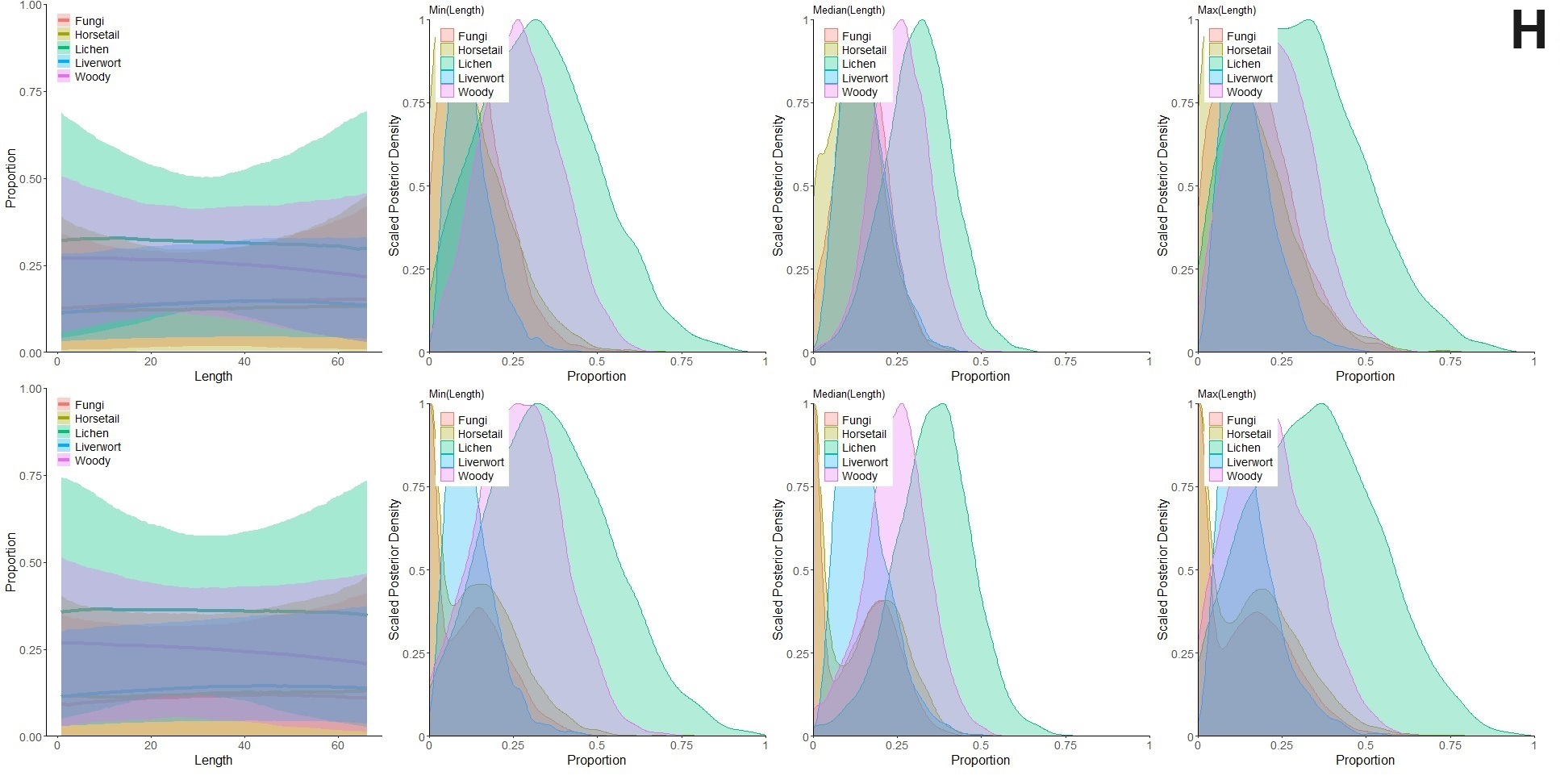


**Supplementary Figure 3H.** *Top row (left to right)*: Estimated diet mixture proportions throughout the antler’s length for sample CMN39132 using uninformative priors, followed by the scaled posterior density versus proportion for each food source measured at three different intervals throughout the antler (the minimum length of the antler (i.e., the base), the middle length of the antler (i.e., the middle), and the maximum length of the antler (i.e., the tip). *Bottom row (left to right)*: Estimated diet mixture proportions throughout the antler’s length for sample CMN39132 using informative priors, followed by the scaled posterior density versus proportion for each food source measured at three different intervals throughout the antler (the minimum length of the antler (i.e., the base), the middle length of the antler (i.e., the middle), and the maximum length of the antler (i.e., the tip).


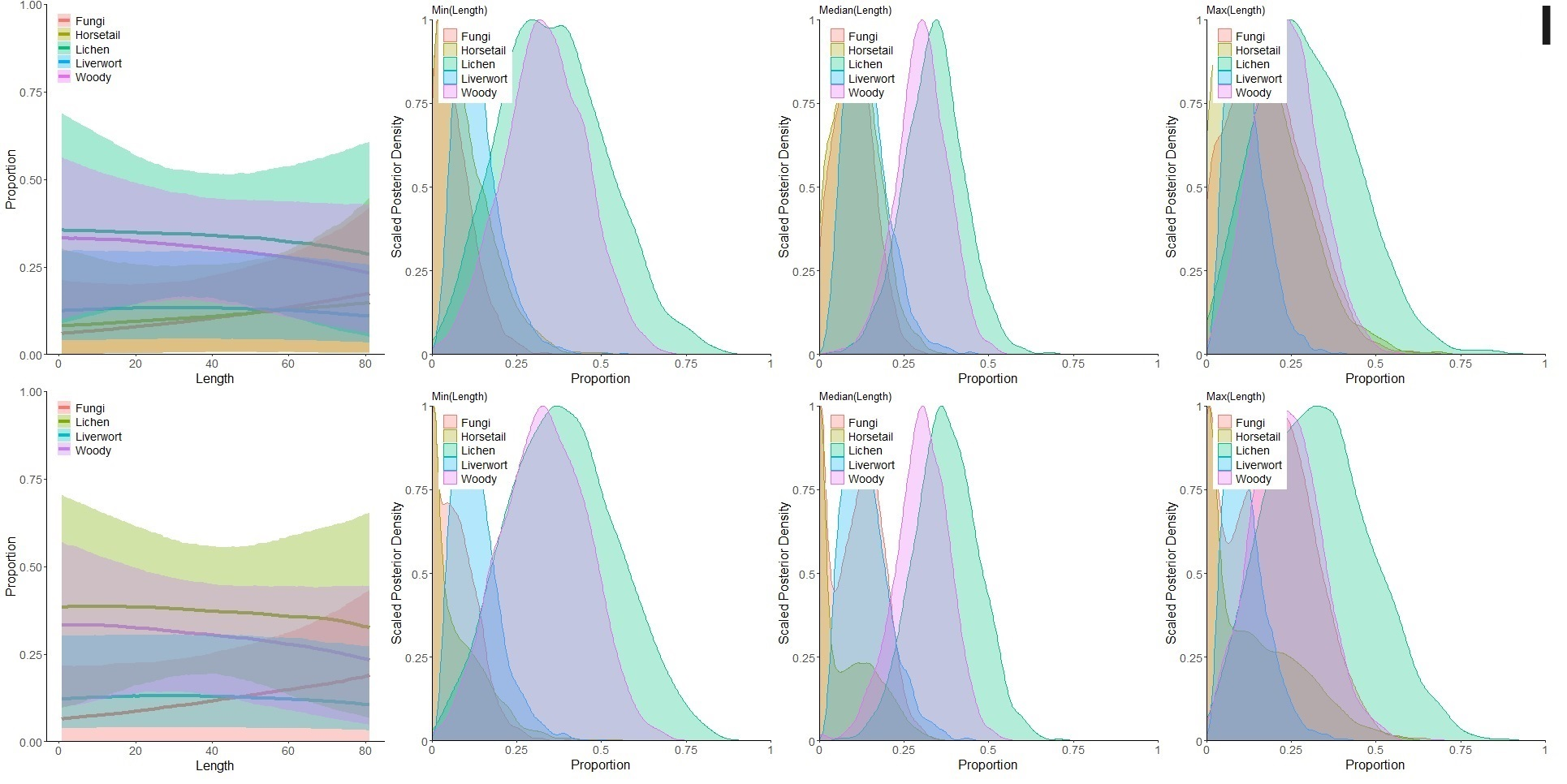


**Supplementary Figure 3I.** *Top row (left to right)*: Estimated diet mixture proportions throughout the antler’s length for sample CMN39145 using uninformative priors, followed by the scaled posterior density versus proportion for each food source measured at three different intervals throughout the antler (the minimum length of the antler (i.e., the base), the middle length of the antler (i.e., the middle), and the maximum length of the antler (i.e., the tip). *Bottom row (left to right)*: Estimated diet mixture proportions throughout the antler’s length for sample CMN39145 using informative priors, followed by the scaled posterior density versus proportion for each food source measured at three different intervals throughout the antler (the minimum length of the antler (i.e., the base), the middle length of the antler (i.e., the middle), and the maximum length of the antler (i.e., the tip).


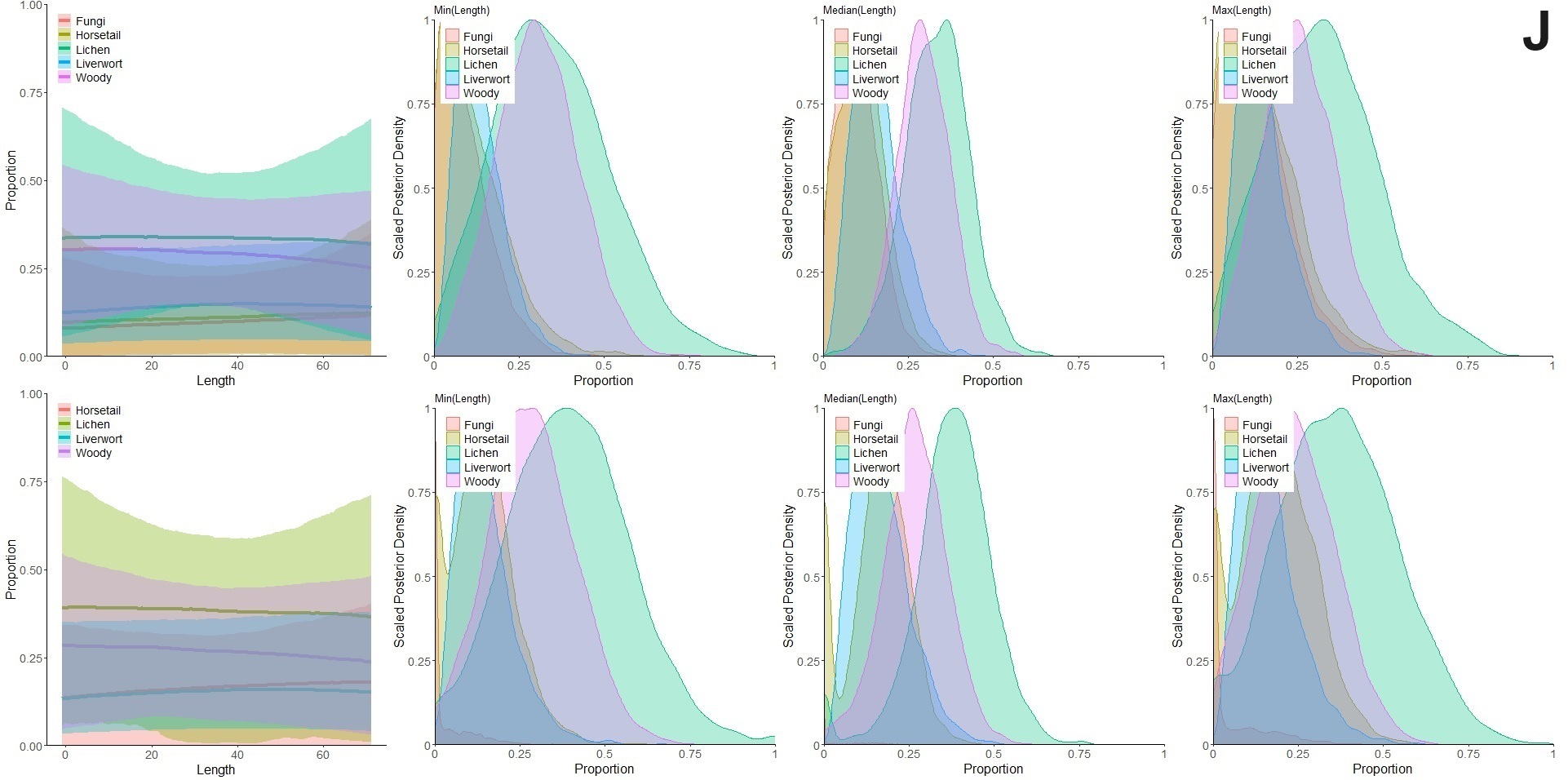


**Supplementary Figure 3J.** *Top row (left to right)*: Estimated diet mixture proportions throughout the antler’s length for sample CMN39148 using uninformative priors, followed by the scaled posterior density versus proportion for each food source measured at three different intervals throughout the antler (the minimum length of the antler (i.e., the base), the middle length of the antler (i.e., the middle), and the maximum length of the antler (i.e., the tip). *Bottom row (left to right)*: Estimated diet mixture proportions throughout the antler’s length for sample CMN39148 using informative priors, followed by the scaled posterior density versus proportion for each food source measured at three different intervals throughout the antler (the minimum length of the antler (i.e., the base), the middle length of the antler (i.e., the middle), and the maximum length of the antler (i.e., the tip).


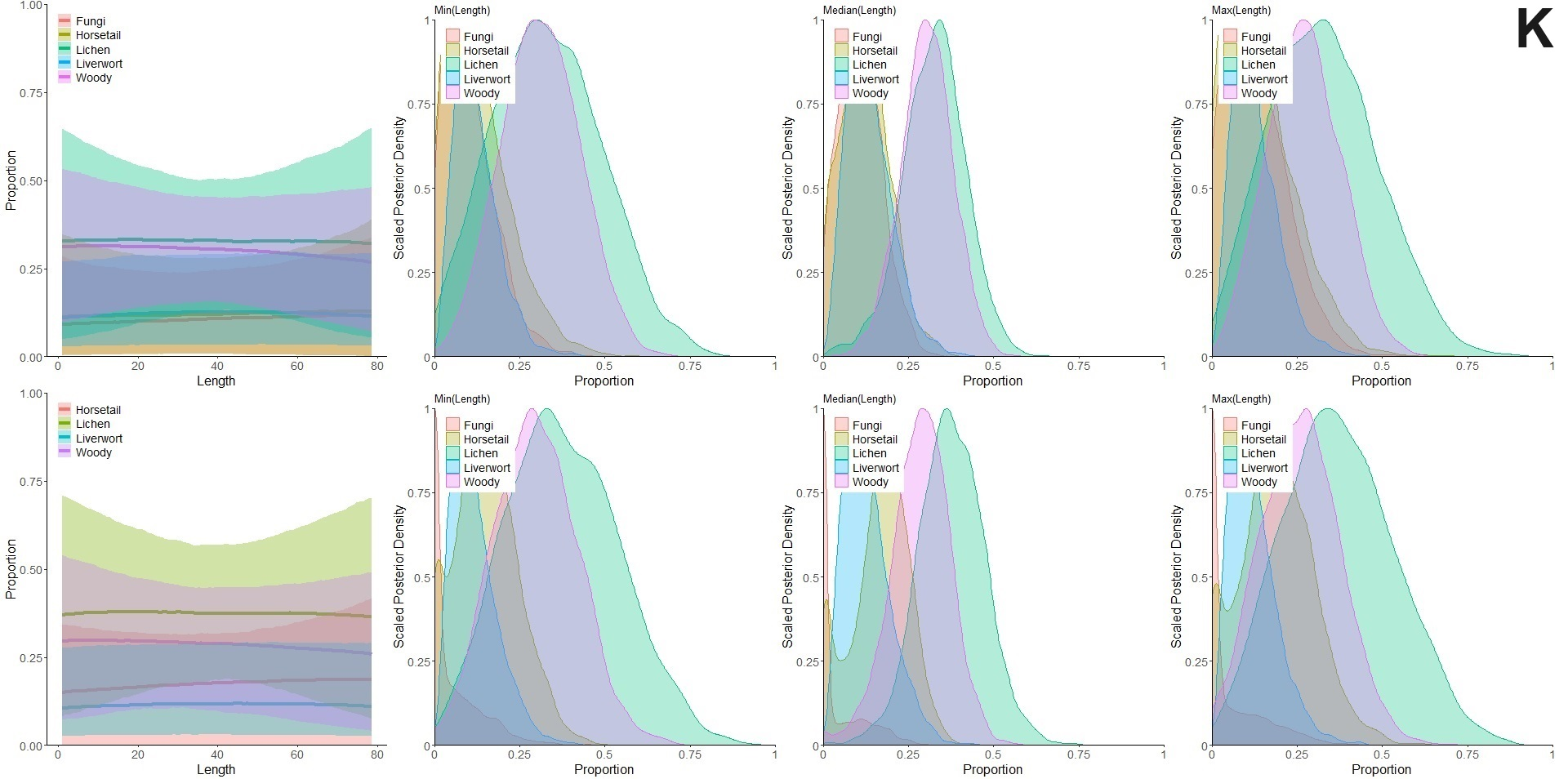


**Supplementary Figure 3K.** *Top row (left to right)*: Estimated diet mixture proportions throughout the antler’s length for sample CMN39149 using uninformative priors, followed by the scaled posterior density versus proportion for each food source measured at three different intervals throughout the antler (the minimum length of the antler (i.e., the base), the middle length of the antler (i.e., the middle), and the maximum length of the antler (i.e., the tip). *Bottom row (left to right)*: Estimated diet mixture proportions throughout the antler’s length for sample CMN39149 using informative priors, followed by the scaled posterior density versus proportion for each food source measured at three different intervals throughout the antler (the minimum length of the antler (i.e., the base), the middle length of the antler (i.e., the middle), and the maximum length of the antler (i.e., the tip).


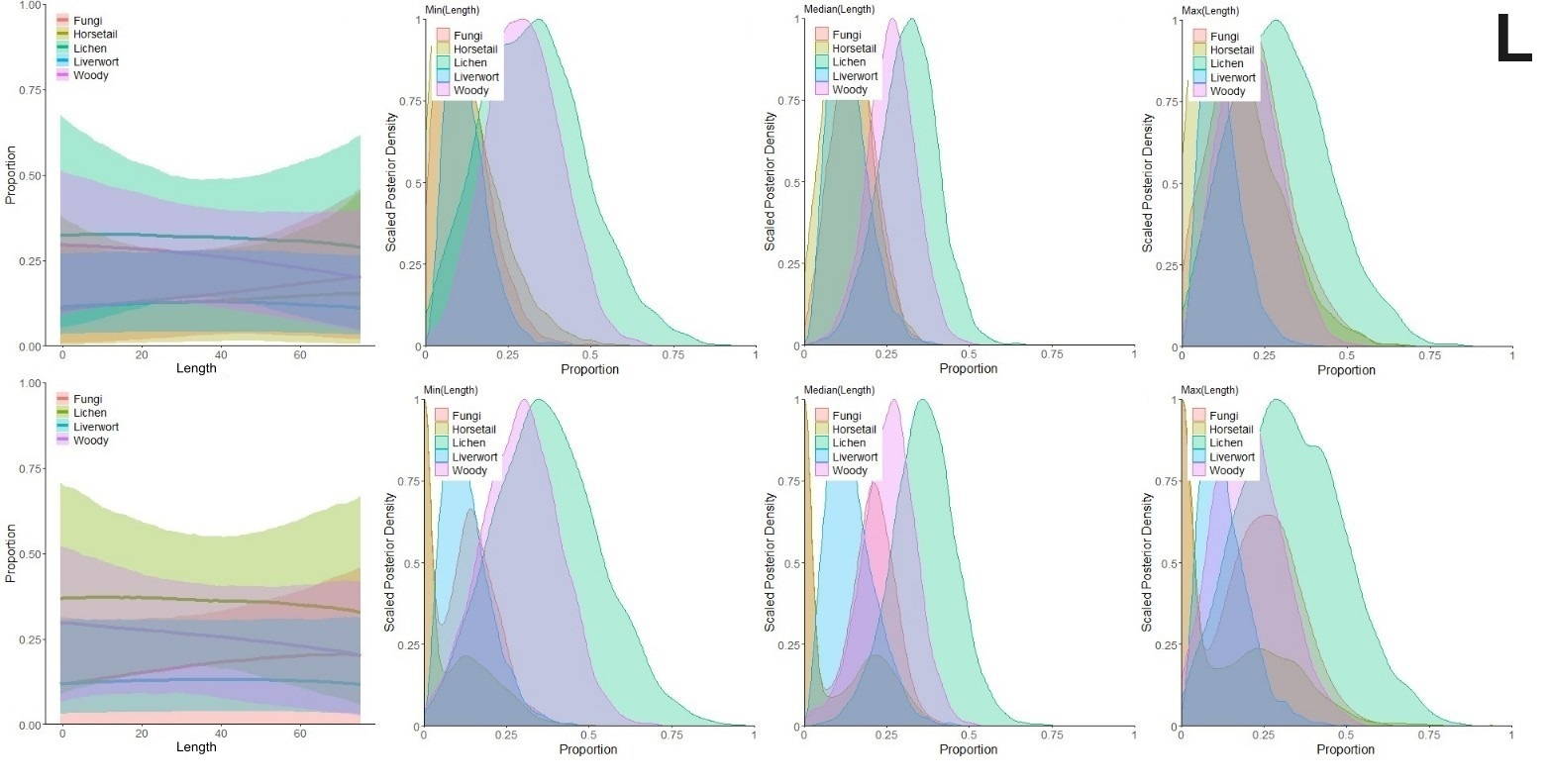


**Supplementary Figure 3L.** *Top row (left to right)*: Estimated diet mixture proportions throughout the antler’s length for sample CMN39151 using uninformative priors, followed by the scaled posterior density versus proportion for each food source measured at three different intervals throughout the antler (the minimum length of the antler (i.e., the base), the middle length of the antler (i.e., the middle), and the maximum length of the antler (i.e., the tip). *Bottom row (left to right)*: Estimated diet mixture proportions throughout the antler’s length for sample CMN39151 using informative priors, followed by the scaled posterior density versus proportion for each food source measured at three different intervals throughout the antler (the minimum length of the antler (i.e., the base), the middle length of the antler (i.e., the middle), and the maximum length of the antler (i.e., the tip).


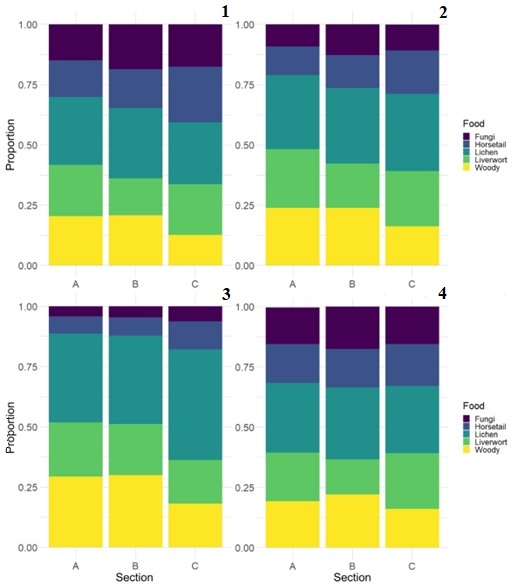


**Supplementary Figure 4A.** Dietary proportions estimated from antlers with all effects set to NULL. Diet proportions were found for the lower (A), the middle (B), and the upper (C) antler segment for each individual antler: **(1)** CMN39090; **(2)** CMN39102; **(3)** CMN39107; **(4)** CMN39108**.**


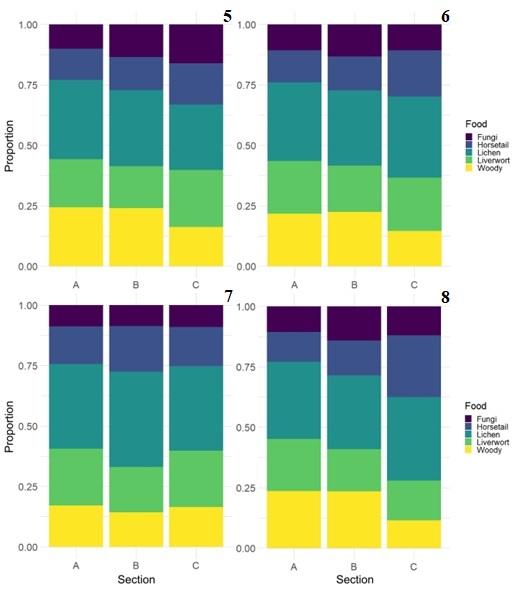


**Supplementary Figure 4B.** Dietary proportions estimated from antlers with all effects set to NULL. Diet proportions were found for the lower (A), the middle (B), and the upper (C) antler segment for each individual antler: **(5)** CMN39110; **(6)** CMN39132; **(7)** CMN39149; **(8)** CMN39151.


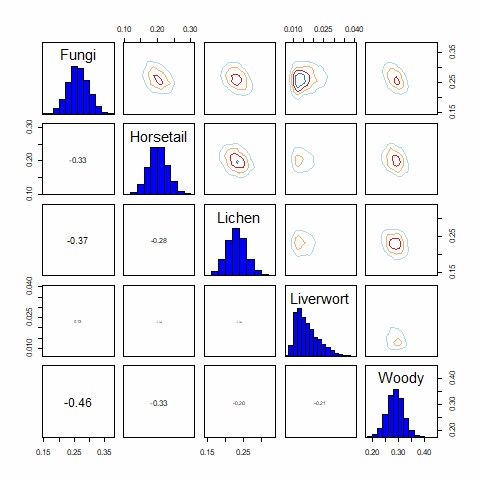


**Supplementary Figure 5A.** Matrix plot for Bayesian stable isotope mixing model using all antler tissue with uninformative priors and no effects.


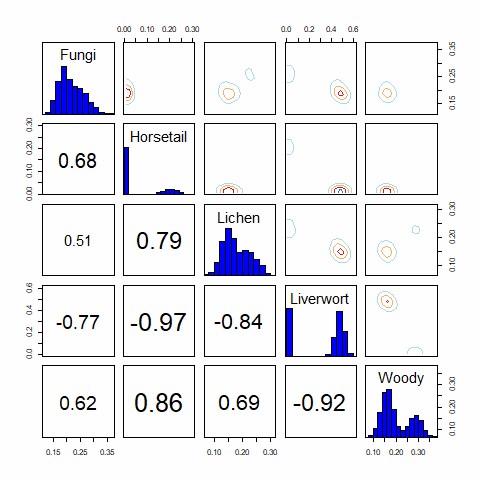


**Supplementary Figure 5B.** Matrix plot for Bayesian stable isotope mixing model using all antler tissue with uninformative priors and antler length as a continuous covariate.


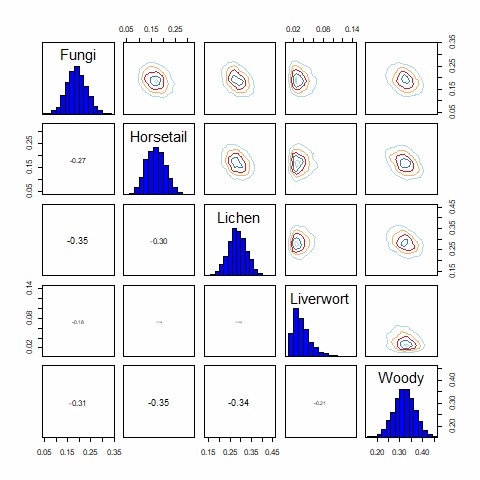


**Supplementary Figure 5C.** Matrix plot for Bayesian stable isotope mixing model using the lower segment of antler tissue with uninformative priors and no effects.


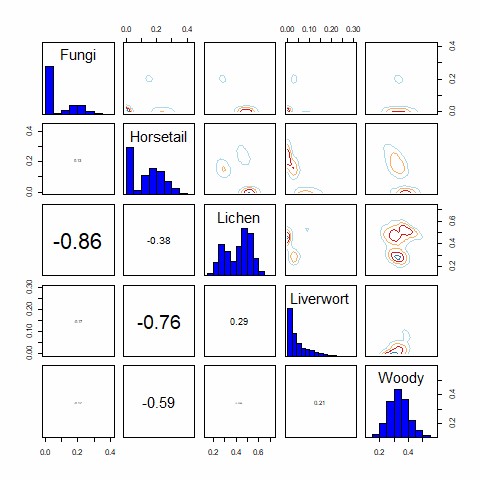


**Supplementary Figure 5D.** Matrix plot for Bayesian stable isotope mixing model using the lower segment of antler tissue with uninformative priors and antler length as a continuous covariate.


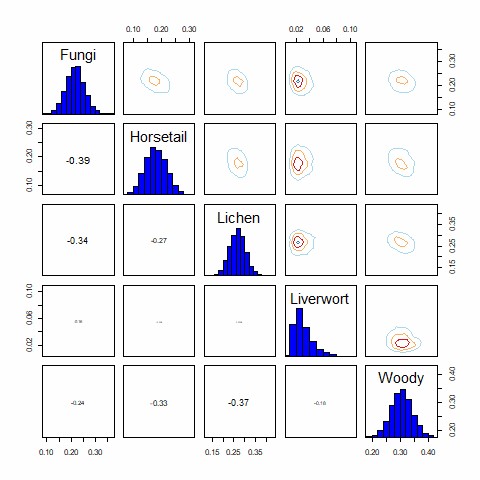


**Supplementary Figure 5E.** Matrix plot for Bayesian stable isotope mixing model using the middle segment of antler tissue with uninformative priors and no effects.


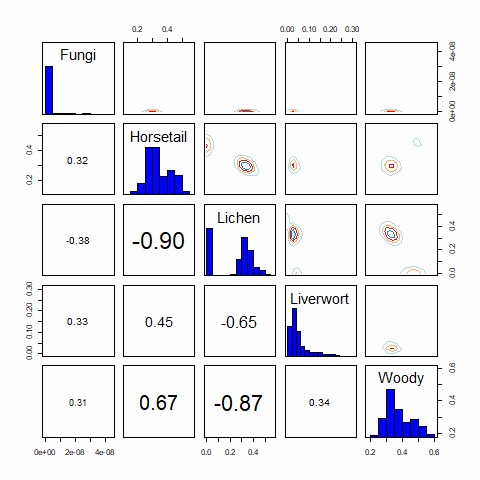


**Supplementary Figure 5F.** Matrix plot for Bayesian stable isotope mixing model using the middle segment of antler tissue with uninformative priors and antler length as a continuous covariate.


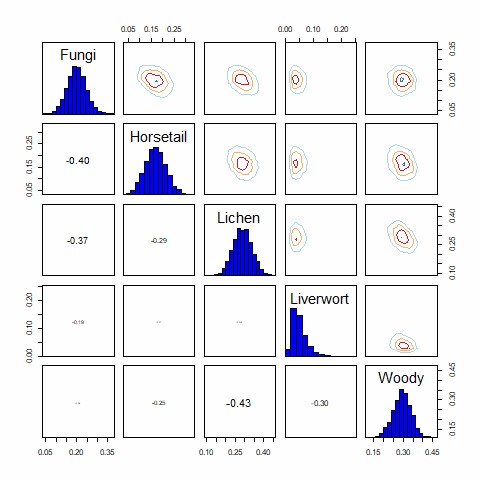


**Supplementary Figure 5G.** Matrix plot for Bayesian stable isotope mixing model using the upper segment of antler tissue with uninformative priors and no effects.


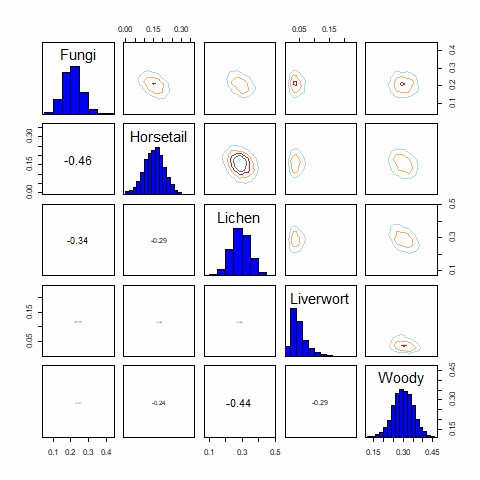


**Supplementary Figure 5H.** Matrix plot for Bayesian stable isotope mixing model using the upper segment of antler tissue with uninformative priors and antler length as a continuous covariate.


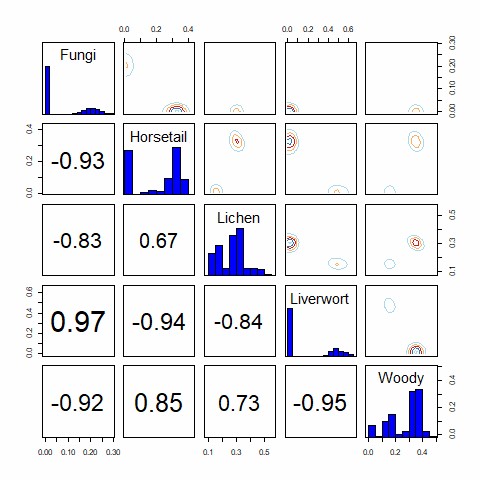


**Supplementary Figure 5I.** Matrix plot for Bayesian stable isotope mixing model using all antler tissue with informative priors and no effects.


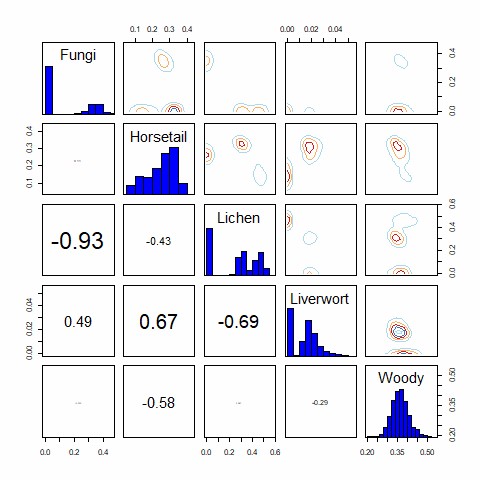


**Supplementary Figure 5J.** Matrix plot for Bayesian stable isotope mixing model using all antler tissue with informative priors and antler length as a continuous covariate.


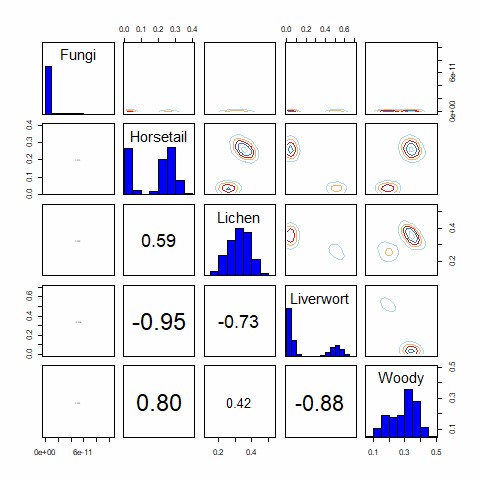


**Supplementary Figure 5K.** Matrix plot for Bayesian stable isotope mixing model using the lower segment of antler tissue with informative priors and no effects.


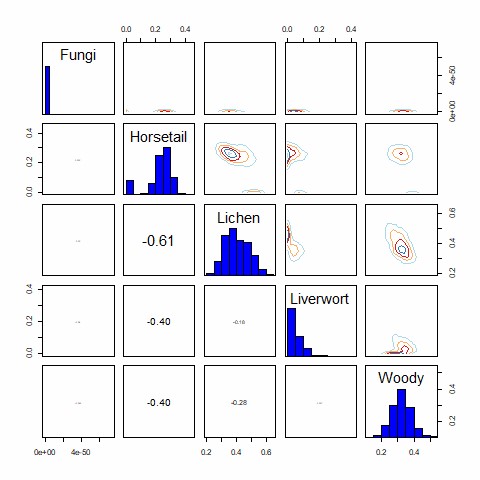


**Supplementary Figure 5L.** Matrix plot for Bayesian stable isotope mixing model using the lower segment of antler tissue with informative priors and antler length as a continuous covariate.


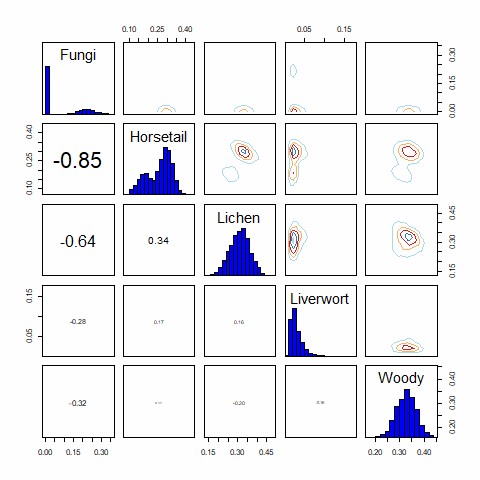


**Supplementary Figure 5M.** Matrix plot for Bayesian stable isotope mixing model using the middle segment of antler tissue with informative priors and no effects.


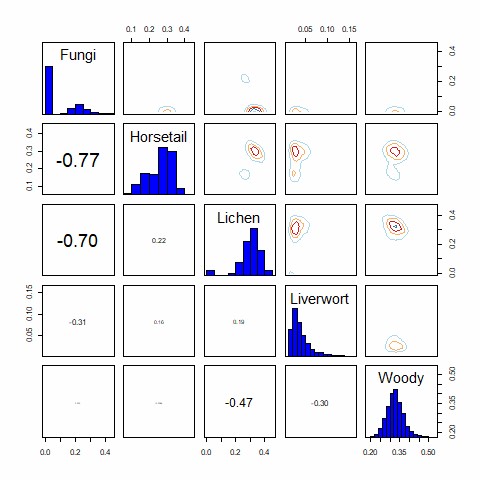


**Supplementary Figure 5N.** Matrix plot for Bayesian stable isotope mixing model using the middle segment of antler tissue with informative priors and antler length as a continuous covariate.


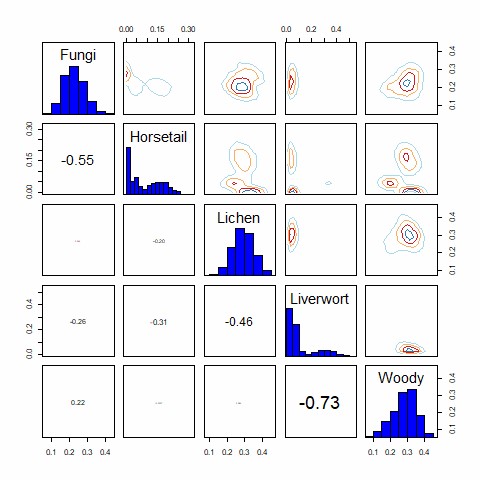


**Supplementary Figure 5O.** Matrix plot for Bayesian stable isotope mixing model using the upper segment of antler tissue with informative priors and no effects.


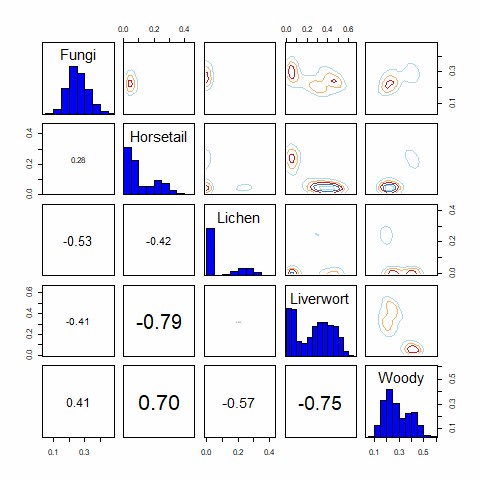


**Supplementary Figure 5P.** Matrix plot for Bayesian stable isotope mixing model using the upper segment of antler tissue with informative priors and antler length as a continuous covariate.
